# A dominant mutation in tomato DNA POLYMERASE DELTA 1 causes geminivirus DNA replication catastrophe

**DOI:** 10.1073/pnas.2531841123

**Published:** 2026-08-24

**Authors:** Deri Gustian, Chen-Hsin Yu, Fuh-Jyh Jan, Wilhelm Gruissem

**Affiliations:** ^a^Department of Plant Pathology, National Chung Hsing University, Taichung 40227, Taiwan; ^b^ Institute of Molecular Biology, Academia Sinica, Taipei 115, Taiwan; ^c^Biotechnology Center, National Chung Hsing University, Taichung 40227, Taiwan

**Keywords:** geminivirus, infection, DNA POLYMERASE DELTA 1, DNA replication, resistance

## Abstract

Geminiviruses pose a severe threat to grain and vegetable crops worldwide, often resulting in significant economic losses. In cultivated tomato (*Solanum lycopersicum*), *Ty* resistance alleles have been introduced from wild tomato relatives, providing partial to strong resistance to geminivirus infections. The *Ty-6* resistance locus from *Solanum chilense* was previously mapped to chromosome 10. It was recently shown to contain a mutant allele of the *DNA POLYMERASE DELTA 1* (*POLD1*) gene that provides resistance to Tomato yellow leaf curl virus (TYLCV) infections. However, the resistance mechanism remained unknown. Here, we report another *POLD1* allele at the *Ty-6* locus of *S. chilense* with an E622D mutation in the catalytic site of the POLD1 protein. *POLD1^E622D^* is maintained as a heterozygous dominant allele in *S. chilense* and the AVTO2225 breeding line. It provides full resistance to the severe TYLCV Thailand (TYLCTHV) strain. The E622D amino acid change does not alter the predicted structure of POLD1. Replication of the TYLCTHV genome in plants carrying the *POLD1^E622D^* allele is severely compromised by a high frequency of mutations that accumulate in viral DNA, which results in nonfunctional proteins that are essential for continuous viral replication. Ectopically expressing the *POLD1^E622D^* allele cDNA alone causes mutations in TYLCTHV genes in inoculated leaves. *S. chilense* and AVTO2225 plants carrying the *POLD1^E622D^* allele mount a hypersensitive response after TYLCTHV infection, indicating that the defective virus genome cannot suppress the plant defense. The dominant *POLD1^E622D^* allele is therefore an effective resistance gene that geminiviruses cannot overcome.

## Significance

The cultivated tomato is the most widely grown vegetable crop worldwide, but its production is severely impacted by geminivirus infections. Therefore, finding robust resistance genes against these infections would be of significant economic benefit. Recently, mutations in DNA POLYMERASE DELTA 1 (POLD1) have been associated with resistance to geminivirus infections. However, the mechanism by which these mutations confer resistance remains unknown. In tomato, mutant POLD1 alleles are located at the *Ty-6* resistance locus introgressed from the wild tomato *Solanum chilense*. The mutant POLD1 enzyme reported here introduces mutations into the viral genome during replication, thereby disrupting the function of essential viral proteins. Currently, the mutant POLD1-mediated inactivation of geminiviruses is a powerful resistance mechanism that plant geminiviruses cannot escape.

Geminiviruses (genus *Begomovirus*, family *Geminiviridae*) are among the most damaging groups of plant viruses worldwide and affect a wide range of important food and cash crops in tropical and subtropical regions (1–4). The viruses cause chlorosis and reduced photosynthesis, leaf curling, stunting, and severe yield losses, which often have devastating socioeconomic consequences, particularly for smallholder farmers (5–8). The impact of geminiviruses is amplified by the global spread of their whitefly vector *Bemisia tabaci*, driven by crop intensification, plant trade, insect resistance, and also climate change (9–13). Given the impact of geminiviruses on crop production, several resistance genes (R genes) and resistance mechanisms have been identified against viral infections. The R genes are often found in crop wild relatives and can limit virus replication, movement, or symptom development (14). They have been key targets in crop breeding programs to control geminivirus-associated diseases.

The tomato (*Solanum lycopersicum*) is one of the most important horticultural crops worldwide in terms of both nutritional and health contributions as well as economic value (15–18). It is traded as a fresh vegetable and in processed forms, and the global market is valued at over USD 200 billion (19, 20). However, the tomato is a major target of geminiviruses, especially the TYLCV disease, which can result in losses between 20 to 100 percent in severe cases (21–23). Several R genes, termed *Ty* genes, have been identified in wild relatives and introduced into commercial tomato varieties (24, 25). The *Solanum chilense Ty-1* and *Ty-3* genes are two alleles of the *RNA-DEPENDENT RNA POLYMERASE* (*RDR*) gene and enhance the transcriptional gene silencing of TYLCV (26–29). The *Ty-2* gene, originating from *Solanum habrochaites*, encodes an NBS-LRR protein that blocks geminivirus replication (30–32). The function of the *Ty-4* gene from *S. chilense* is unknown, but it has a weak effect and is additive when combined with other *Ty* genes (33, 34). The *Solanum peruvianum Ty-5* gene functions as a recessive resistance and encodes the translation regulator Pelota (35–37); however, the mechanism by which Pelota interferes with geminivirus replication remains unclear. The *Ty-6* gene provides resistance to multiple begomovirus species (24) and was introgressed into commercial tomato varieties from *S. chilense*. In cultivated tomato, the introgressed *Ty-6* gene was mapped to chromosome 10 (38) and was recently reported to encode DNA POLYMERASE δ 1 (POLD1), which has a mutation in its exonuclease domain (39). Previously, the dominant CMD2 R locus in cassava was identified and encodes genes for POLD1 mutant enzyme variants in several different cassava accessions that are resistant to ACMV infections (40). Thus, POLD1 emerges as an important R gene for resistance to geminivirus infections in crops. However, how the mutant POLD1 enzymes affect virus DNA replication and function remains unknown.

Geminiviruses replicate by a rolling circle mechanism but encode only few replication-related proteins and, therefore, rely on the host DNA replication machinery to amplify their DNA in the nuclei of infected plants (41, 42). The viral replication-associated protein Rep reprograms the host cell into an S phase-like state by interacting with the plant retinoblastoma-related protein (RBR) to release E2F transcription factors. These factors then activate host cell replication-related genes (43, 44). The viral Rep and replication enhancer REn proteins together are essential for efficient geminivirus DNA replication. They recruit host POLD1 to the viral genome origin of DNA replication and assemble the viral DNA replication complex to produce the virus single-strand DNA molecule (45–47). Therefore, mutations in POLD1 may compromise viral genome replication and interfere with viral functions. Here, we report that a single nonsynonymous mutation in a tomato *POLD1* allele located at the *Ty-6* locus provides resistance to infection with TYLCTHV, a severe bipartite genome geminivirus strain from Thailand (48). The mutation is located in the catalytic domain of POLD1, and the mutant enzyme introduces random nucleotide changes and insertions/deletions (INDELs) into the viral genome at high frequency, inactivating the virus. This DNA replication catastrophe is a novel geminivirus resistance mechanism that can now be evaluated for deployment in agricultural crops, particularly to benefit smallholder farmers.

## Results

### AVTO2225 and AVTO2226 Plants Contain Combinations of *Ty-1*, *Ty-2,* and *Ty-6* Resistance Markers.

We first genotyped the tomato AVTO2225 cultivar and AVTO2226 (*S. chilense* accession LA2779) for markers diagnostic of the *Ty-1*, *Ty-2,* and *Ty-6* resistance alleles (30, 38, 49). *SI Appendix*, Fig. S1 and Table S1 summarize that the AVTO2225 and AVTO2226 lines have mixed combinations of the *Ty1*, *Ty-2,* and *Ty-6* markers. PCR and DNA sequence analysis confirmed that the *Ty-1* CAPS marker was from the DFDGD-class γ-type *RDR* gene (29) (*SI Appendix*, Fig. S1 *A* and *B*), which is expressed at low levels in CLN1558A and at higher levels in AVTO2225 and AVTO2226 (*SI Appendix*, Fig. S1*C*). Most of the AVTO2225 and AVTO2226 plants also have the *Ty-2* marker (*SI Appendix*, Fig. S1*D*), which corresponds to the DNA sequence of the SCAR marker associated with an *NBS-LRR* gene (*SI Appendix*, Fig. S1*E*) (30). This gene is also expressed at low levels in CLN1558A and more highly in AVTO2225 and AVTO2226 (*SI Appendix*, Fig. S1*F*). The *Ty-6* resistance marker on tomato chromosome 10 shows 500 bp and 200 bp PCR products using the primers previously reported for mapping the *Ty-6* locus (38). The 200 bp PCR product of the *Ty-6* CAPS marker was not present in CLN1558A plants and detected only in six AVTO2225 plants, whereas it was present in all AVTO2226 plants (*SI Appendix*, Fig. S1*G*). This suggests that the *Ty-6* CAPS marker was introgressed from *S. chilense* LA2779 in AVTO2225, but AVTO2225 plants are heterozygous and segregating for the *Ty-6* CAPS marker. DNA sequence analysis revealed that the 500 bp PCR product belongs to a *CALMODULIN-2* gene on chromosome 10. However, the DNA sequence of the *Ty-6* CAPS marker could not be assigned to the same tomato gene on chromosome 10 (*SI Appendix*, Fig. S1*H*) in the available *S. lycopersicon* genome sequences. It also does not map to chromosome 10 in the *S. chilense* LA3111 and LA1972 sequenced genomes, but these accessions do not have the *Ty-6* resistance (50, 51). These results suggest that the chromosome region associated with *Ty-6* resistance in the *S. chilense* LA2779 accession and introgressed into AVTO2225 is polymorphic. As expected, the *Ty-1* and *Ty-2* markers were not detected in the wild-type CLN1558A plants (*SI Appendix*, Fig. S1*I*).

### AVTO2225 and AVTO2226 Plants with the *Ty-6* Marker Are Resistant to TYLCTHV Infections.

**Fig. 1.**
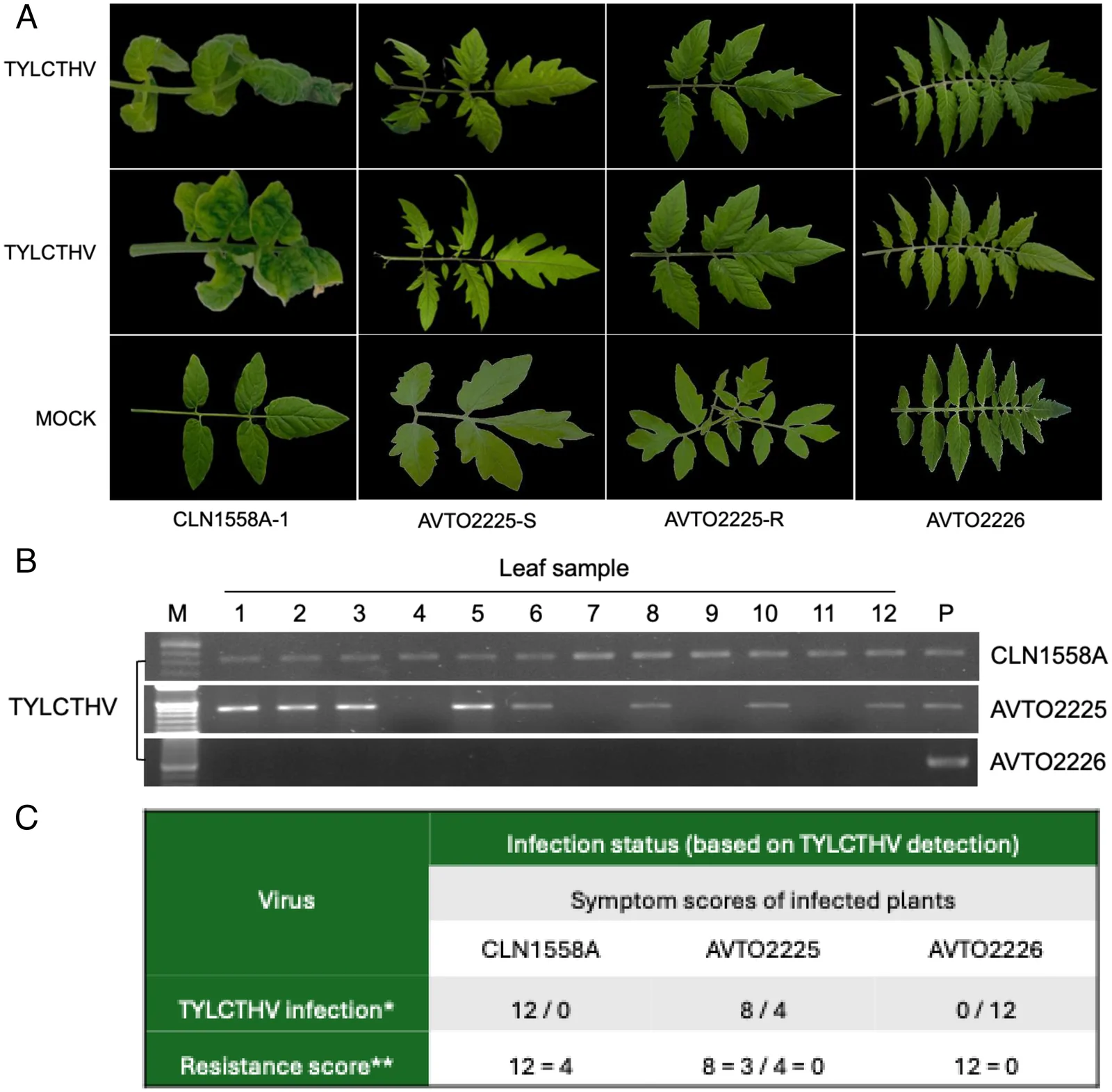
Symptom development, TYLCTHV DNA detection, and symptom scores in CLN1558A, AVTO2225, and AVTO226 (*S. chilense*) plants infected with TYLCTHV. (*A*) Symptoms at 30 dpi of CLN1558A-1 and 2, AVTO2225-S-1 and 2, AVTO2225-R-4 and 7, and AVTO2226-1 and 2 plants (*SI Appendix*, Table S1) after infection with TYLCTHV are shown as examples (see also *SI Appendix*, Fig. S1*J* for full details). (*B*) PCR detection of TYLCTHV DNA in systemic leaves collected from 12 plants of each CLN1558A, AVTO2225, and AVTO226 line at 30 dpi. AVTO2225-4, 7, 9, and 11 and all AVTO2226 genotypes have the *Ty-6* resistance allele (*SI Appendix*, Table S1). M = DNA marker; P = plasmid containing the infectious TYLCTHV clone as a PCR control. (*C*) CLN1558A, AVTO2225, and AVTO226 lines were scored for resistance to TYLCTHV infection at 30 dpi. * indicates 12 plants of CLN1558A and plants 1 to 12 each of AVTO2225 and AVTO2226 genotypes (*SI Appendix*, Table S1) were tested for resistance to infection with TYLCTHV. x/y, where x is the number of TYLCTHV-susceptible plants with symptoms and y is the number of TYLCTHV-resistant plants without symptoms. ** For the resistance score, the severity of symptoms caused by the TYLCTHV infections were assessed visually using a scale of 0 to 4 (explained in *Materials and Methods*). See *Materials and Methods*, *SI Appendix*, Figs. S1*J* and S2 for more details and the statistical analysis of the median symptom scores.

Next, we inoculated leaves of sets of 12 AVTO2225 and AVTO2226 clonal plants (derived from AVTO2225 plants 1 to 12 and AVTO2226 plants 1 to 12 listed in *SI Appendix*, Table S1), as well as 12 CLN1558A plants grown from seeds, with Agrobacteria carrying an infectious clone of the TYLCTHV bipartite genome consisting of the DNA-A segment and the DNA-B segment. As expected, at 30 dpi CLN1558A plants were infected with TYLCTHV as revealed by typical plant symptoms (Fig. 1*A* and *SI Appendix*, Figs. S1*J* and S2), detection of the TYLCTHV DNA in systemic leaves (Fig. 1*B* and *SI Appendix*, Figs. S2 and S3), and symptom scores (Fig. 1*C* and *SI Appendix*, Figs. S1*J* and S2). TYLCTHV produces strong infection symptoms and the highest symptom scores in CLN1558A, as indicated by severe leaf necrosis. AVTO2225-4, 7, 9, and 11 plants, which have the *Ty-6* CAPS marker (*SI Appendix*, Fig. S1*G*), did not exhibit symptoms on inoculated or other leaves when infected with TYLCTHV (Fig. 1*A* and *SI Appendix*, Figs. S1*J* and S2; hereafter called AVTO2225-R plants). AVTO2225 plants that do not have the *Ty-6* CAPS marker became infected with TYLCTHV, though the symptoms were milder and the symptom score was lower compared to CLN1558A (Fig. 1 *A* and *C* and *SI Appendix*, Figs. S1*J* and S2; hereafter called AVTO2225-S plants). This is expected, as all 12 AVTO2225 plants carry the *Ty-2* allele or a combination of the *Ty-1* and *Ty-2* alleles (*SI Appendix*, Table S1), which provide partial resistance to TYLCTHV (52). All 12 AVTO2226 plants have the *Ty-6* CAPS marker (*SI Appendix*, Fig. S1*G*), as well as the markers for the *Ty-2* allele or a combination of the *Ty-1* and *Ty-2* alleles (*SI Appendix*, Table S1). These plants were resistant to infection by TYLCTHV as indicated by the absence of symptoms on the plant leaves and a symptom score of zero (Fig. 1 *A* and *C* and *SI Appendix*, Figs. S1*J* and S2). PCR of DNA isolated from the second leaf below the shoot apical meristem confirmed the absence of TYLCTHV in all AVTO2226 plants and in the AVTO2225-R plants with the *Ty-6* marker (Fig. 1*B*), indicating that TYLCTHV cannot move systemically in the plants from the inoculated leaf. Quantification of TYLCTHV DNA levels in the inoculated leaves at 7, 14, and 21 dpi was consistent with the visible symptom development (*SI Appendix*, Fig. S3). TYLCTHV DNA levels rose in CLN1558A and AVTO2225-S leaves until 7 dpi and then persisted, indicating that the virus was replicating in the inoculated leaf and systemically moving in the plants (*SI Appendix*, Fig. S3 *A* and *B*). In contrast, TYLCTHV DNA levels in AVTO2225-R and AVTO2226 plants did not rise from the low levels at 7 dpi but instead declined, suggesting that the virus DNA was replicating initially in the resistant plants, but DNA replication did not continue (*SI Appendix*, Fig. S3 *B* and *C*).

### AVTO222-R and AVTO2226 Plants Carry a Heterozygous *POLD1* Mutant Allele.

**Fig. 2.**
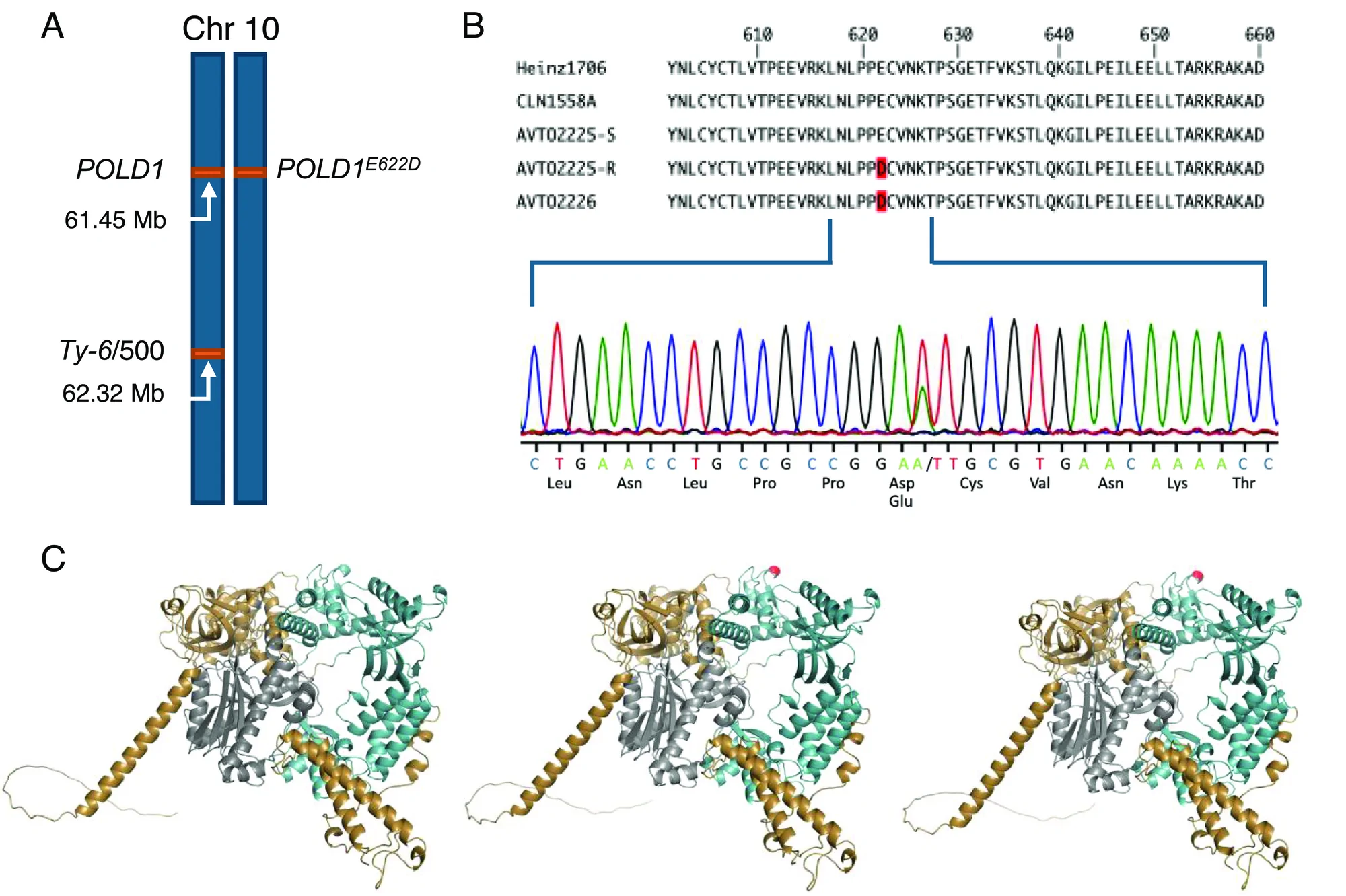
DNA sequence analysis of *POLD1* and *POLD1^E622D^* alleles and structural predictions of their proteins. (*A*) The position of the 500 bp PCR product obtained with the DNA primers for the *Ty-6* CAPS marker (38) is shown relative to the position of the *POLD1* allele on chromosome 10 in resistant AVTO2225 (AVTO2225-R) plants and in AVTO2226 plants. (*B*) The POLD1 protein sequences of AVTO2225-R and AVTO2226 plants exhibit an E to D amino acid substitution at position 622 (*Top*), which is corroborated by a single A/T nucleotide polymorphism in the DNA Sanger sequence trace of AVTO2226 *POLD1^E622D^* (*Bottom*; see *SI Appendix*, Fig. S3 for the DNA Sanger sequence trace of AVTO2225-R *POLD^E622D^*). The E622D mutation is linked to the *Ty-6* CAPS marker and responsible for resistance to TYLCTHV infection. (*C*) AlphaFold3 structural predictions of AVTO2225 POLD1 (*Left*), AVTO2225 POLD1^E622D^ (with the position of the E to D amino acid change indicated in red; center panel), and AVTO2226 POLD1^E622D^ (*Right*). The POLD1 catalytic domain is marked in blue, and the exonuclease domain is marked in gray. Additional POLD1 AlphaFold3 models are shown in *SI Appendix*, Fig. S5.

The 500 bp PCR product obtained with the primers for the *Ty-6* CAPS marker is located at 62.32 Mb on tomato chromosome 10, near the region containing the *POLD1* gene at 61.45 Mb (Fig. 2*A*). Previously, mutations in the cassava and tomato *POLD1* genes that confer resistance to begomoviruses were identified (39, 40). Therefore, we sequenced the *POLD1* gene in CLN1558A, AVTO2225-S, AVTO-2225-R, and AVTO2226 plants. When aligned with the POLD1 protein sequence of the Heinz 1706 reference genome (53, 54), the POLD1 protein sequences were largely identical and revealed only a small number of amino acid changes between the Heinz 1706 POLD1 reference protein and the CLN1558A, AVTO2225, and AVTO2226 POLD1 proteins (*SI Appendix*, Fig. S4*A*). Based on the Sanger DNA sequence traces, all nonsynonymous nucleotide changes for the amino acid polymorphisms are homozygous in CLN1558A, AVTO2225, and AVTO2226, except for the nonsynonymous nucleotide change that causes a change of glutamic acid (E) to aspartic acid (D) at position 622 of POLD1 and is heterozygous in the TYLCTHV-resistant AVTO2225-R and AVTO2226 plants (Fig. 2*B* and *SI Appendix*, Fig. S4*B*) but is homozygous for glutamic acid (E) in the AVTO2225-S plants and in CLN1558A. These results suggest that the *Ty-6* resistance to TYLCTHV in the AVTO2225-R and AVTO2226 plants is associated with a heterozygous dominant E622D tomato *POLD1* mutant allele (hereafter *POLD1^E622D^*), similar to the heterozygous dominant *POLD1* mutant alleles in the TME cassava cultivars that are resistant to African cassava begomoviruses (40). Not all of the other amino acid changes are shared by the AVTO2225 and AVTO2226 POLD1 and POLD1^E622D^ proteins (*SI Appendix*, Fig. S4*A*). Therefore, it is possible that the *S. chilense POLD1^E622D^* allele originally introduced into the ANSAL genotype used in the AVTO2225 breeding pedigree (*Materials and Methods*) originates from a different *S. chilense* accession than LA2779 (AVTO2226). A genome sequence is currently not available for the *S. chilense* LA2779.

To confirm the role of the heterozygous POLD1^E622D^ mutation in the AVTO2226 and AVTO2225-R plants (which have the *Ty-6* CAPS marker) in conferring resistance to TYLCTHV, we genotyped the F1 progeny of the susceptible CLN1558A and the selfed resistant AVTO2225-R-9 and AVTO2226-1 plants (*SI Appendix*, Table S1). *SI Appendix*, Fig. S5*A* shows that the *Ty-6* CAPS marker was absent in 40 CLN1558A F1 plants. The 40 F1 plants from each of the AVTO2225-R-9 and AVTO2226-1 parents segregated for the *Ty-6* CAPS marker associated with resistance. Inoculation with TYLCTHV revealed that only the F1 plants from the AVTO2225-9 and AVTO2226-1 parents that retained the *Ty-6* CAPS marker were resistant to infection and did not accumulate TYLCTHV DNA (*SI Appendix*, Fig. S5*B*). Sanger sequencing of the *POLD1* gene revealed that the E622D mutation is present only in the resistant AVTO2225 and AVTO2226 F1 plants that have the *Ty-6* CAPS marker, but not in the susceptible AVTO2225 and AVTO2226 plants (*SI Appendix*, Fig. S5*C*). The genetic segregation analysis confirmed that the *POLD1^E622D^* allele is linked to the *Ty-6* CAPS marker and heterozygous in the AVTO2225-R-9 and AVTO2226-1 parent plants and that the resistance to TYLCTHV is caused by the mutant POLD1^E622D^ enzyme. We could not identify F1 plants in which the *POLD1^E622D^* allele was homozygous (i.e., plants with only the *Ty-6* CAPS marker), suggesting that a homozygous *POLD1^E622D^* allele is likely deleterious during tomato gamete formation or embryo development.

### The Tomato POLD1^E622D^ Enzyme Mutates TYLCTHV DNA During Replication.

Using AlphaFold3, we predicted the structures of the CLN1558A, AVTO2225, and AVTO2226 POLD1 wild-type and POLD1^E622D^ mutant proteins. The models revealed that the E622D mutation and other amino acid polymorphisms do not alter the predicted tomato POLD1 structures (Fig. 2*C* and *SI Appendix*, Fig. S6*A*). Based on calculations using DDMut, the small change in free energy between POLD1 and POLD^E622D^ 1 (ΔΔ^Gstability wt>mut^ = 0.03 kcal/mol) is consistent with the identity of the structural models. Therefore, structural considerations that reduce possible interactions of POLD1^E622D^ with proteins in the TYLCTHV replication complex can likely be excluded as a resistance mechanism. Similar AlphaFold3 models were obtained for the recently reported POLD1^Y515H^ protein (39), showing that the mutation also does not change the structure of the protein (*SI Appendix*, Fig. S6*B*).

**Fig. 3.**
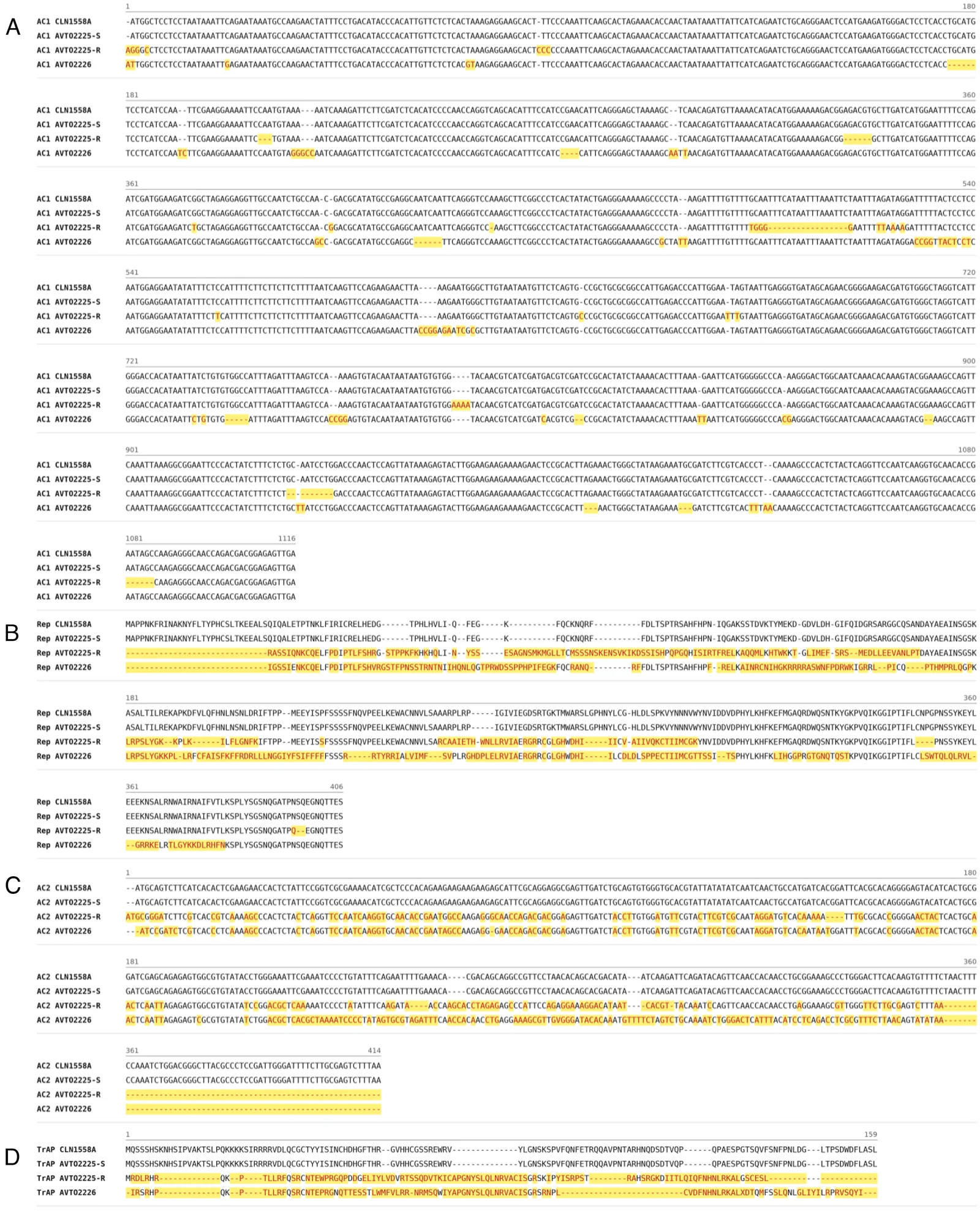
The *POLD1^E622D^* allele causes a virus DNA replication catastrophe in the resistant AVTO2225 and AVTO2226 plants. Examples of TYLCTHV *AC1* and *AC2* DNA and corresponding Rep and TrAP protein sequences, respectively, of the virus extracted from the infected leaves of CLN1558A plants, AVTO2225-S and AVTO2225-R plants, and AVTO2226 plants at 7 dpi. (*A*) The *AC1* DNA sequence reveals high frequency nucleotide changes and INDELs (highlighted in yellow) in TYLCTHV DNA extracted from inoculated AVTO2225-R and AVTO2226 leaves. The number of *AC1* mutations are more extensive in the AVTO2226 leaves. (*B*) The nucleotide changes and INDELs in the *AC1* DNA sequence scramble the corresponding Rep protein sequence, indicating that no functional Rep protein is produced. (*C*) The *AC2* DNA sequence reveals similar high frequency nucleotide changes (yellow) and INDELs in the TYLCTHV DNA extracted from inoculated AVTO2225-R and AVTO2226 leaves. (*D*) The nucleotide changes and INDELs in the *AC2* DNA sequence also scramble the corresponding TrAP protein sequence, indicating that no functional TrAP protein is produced. Results from sequencing TYLCTHV *AC1* and *AC2* genes in additional resistant AVTO2225-R and AVTO-2226 plants are shown in *SI Appendix*, Fig. S6.

The E622D mutation is located in the catalytic domain of the DNA polymerase in the predicted structure of the tomato POLD1 protein (Fig. 2*C*). Therefore, it is possible that the mutation affects the fidelity of the DNA polymerase. In the tomato plants heterozygous for the mutant enzyme, this could damage the integrity of the TYLCTHV genome through mutations introduced by POLD1^E622D^ during DNA replication and perpetuated by the wild-type POLD1 enzyme. To investigate this possibility, we extracted DNA from TYLCTHV-inoculated leaves after 7 d from CLN1558A, AVTO2226, AVTO2225-S, and AVTO2225-R plants, and generated independent clones of the *AC1* and *AC2* genes, which encode Rep and the Transcriptional Activator Protein (TrAP), respectively, that are required for efficient virus DNA replication. Sanger DNA sequencing revealed multiple early frameshifts, insertions, and deletions (INDELs), and random nucleotide changes at high frequency in *AC1* and *AC2* sequences generated during TYLCTHV replication in AVTO2226 and AVTO2225-R plants, but not in susceptible CLN1558 and AVTO2225-S plants (Fig. 3 *A* and *C*, Table 1, and *SI Appendix*, Fig. S7). Considering that plants were inoculated with an infectious TYLCTHV clone, the high frequency of mutations detected in the *AC1* and *AC2* genes in the presence of the *POLD^E622D^* allele far exceed the mutation frequency found in geminivirus populations existing as viral quasispecies in the wild (55). Collectively, these mutations would impair the function of the Rep and TrAP proteins (Fig. 3 *B* and *D*). AVTO2226 plants generate more mutations in the *AC1* and *AC2* genes than AVTO2225-R plants do (Table 1), which is currently difficult to explain. It is possible that other amino acid polymorphisms in AVTO2226 POLD1^E622D^, or mutations in other host proteins of the virus replication complex in *S. chilense* that have not yet been identified, exacerbate the mutagenicity of the enzyme. Regardless of the numbers, the high frequency of random DNA mutations generated by POLD1^E622D^ during TYLCTHV genome replication would affect virus gene functions and movement in tomato plants, resulting in resistance.

**Table 1.** Summary of *AC1* and *AC2* DNA sequence similarity differences caused by high-frequency mutations introduced by POLD^E622D^ in TYLCTHV DNA isolated from resistant AVTO2225-R and AVTO2226 plants relative to TYLCTHV DNA from susceptible CLN1558A and AVTO2225-S plants

TYLCTHV	*AC1* similarity differences (%)			Mean (%)	*AC2* similarity differences (%)			Mean (%)
	1	2	3		1	2	3	
CLN1558A-1	0	0	0	0	0	0	0	0
CLN1558A-2	0	0	0	0	0	0	0	0
CLN1558A-3	0	0	0	0	0	0	0	0
CLN1558A-4	0	0	0	0	0	0	0	0
CLN1558A-5	0	0	0	0	0	0	0	0
CLN1558A-6	0	0	0	0	0	0	0	0
CLN1558A-7	0	0	0	0	0	0	0	0
CLN1558A-8	0	0	0	0	0	0	0	0
CLN1558A-9	0	0	0	0	0	0	0	0
CLN1558A-10	0	0	0	0	0	0	0	0
CLN1558A-11	0	0	0	0	0	0	0	0
CLN1558A-12	0	0	0	0	0	0	0	0
AVTO2225-1-S	0	0	0	0	0	0	0	0
AVTO2225-2-S	0	0	0	0	0	0	0	0
AVTO2225-3-S	0	0	2	0.67	0	0	0	0
AVTO2225-4-R	5.90	5.51	7.71	6.51	8.75	10.35	4.53	7.87
AVTO2225-5-S	0	0	0	0	0	0	0	0
AVTO2225-6-S	0	0	0	0	0	0	0	0
AVTO2225-7-R	12.36	12.90	9.88	11.68	13.75	13.68	12.65	11.17
AVTO2225-8-S	0	0	0	0	0	0	0	0
AVTO2225-9-R	4.55	2.03	3.75	4.11	10.25	13.02	10.26	11.17
AVTO2225-10-S	0	0	0	0	0	0	0	0
AVTO2225-11-R	12.04	20.33	5.89	12.72	10.03	7.64	12.05	9.90
AVTO2225-12-S	0	0	0	0	0	0	0	0
AVTO2226-1	31.02	43.05	–	37.03	20.15	12.76	20.72	17.87
AVTO2226-2	9.58	4.56	27.41	13.88	24.11	19.11	18.75	20.65
AVTO2226-3	35.38	7.10	7.18	16.55	27.15	18.35	21.15	22.21
AVTO2226-4	12.09	9.40	7.89	9.79	17.55	19.32	15.44	17.43
AVTO2226-5	34.75	29.02	27.5	28.75	23.02	21.15	15.85	20.01
AVTO2226-6	47.13	24.31	12.76	28.06	18.26	12.05	14.72	15.01
AVTO2226-7	30.15	21.75	18.8	23.56	24.75	21.13	20.15	22.01
AVTO2226-8	18.76	31.06	34.72	28.18	14.26	13.53	19.75	15.85
AVTO2226-9	18.75	18.80	32.15	23.23	18.11	12.75	18.35	16.40
AVTO2226-10	17.44	13.65	30.15	20.41	20.15	19.75	17.35	19.08
AVTO2226-11	14.53	15.72	21.75	17.33	18.95	12.48	17.15	16.19
AVTO2226-12	14.75	25.72	21.05	20.51	15.15	18.33	16.43	16.64

### The *POLD1^E622D^* Allele Is Sufficient for Reducing Infectivity and Triggering a Hypersensitive Response (HR) to Begomovirus Infection.

To confirm that the TYLCTHV genome underwent functional changes during viral DNA replication, causing the virus to lose infectivity in AVTO2225-R and AVTO2226 plants with the heterozygous *POLD1^E622D^* alleles, we extracted saps containing TYLCTHV DNA from CLN1558A, AVTO2226, AVTO2225-S, and AVTO2225-R leaves 7 d after their inoculation with the virus. We used the leaf saps as an inoculant in a mechanical transmission assay with *Nicotiana benthamiana* and CLN1558A plants. Systemic leaves of *N. benthamiana* inoculated with saps from CLN1558A and AVTO2225-S leaves exhibited typical TYLCTHV infection symptoms, yet systemic leaves remained asymptomatic in plants inoculated with AVTO2225-R or AVTO2226 leaf saps (*SI Appendix*, Fig. S8*A*). PCR analysis confirmed that TYLCTHV DNA was present in symptomatic but not asymptomatic *N. benthamiana* systemic leaves (*SI Appendix*, Fig. S8*B*). All leaf saps contained TYLCTHV DNA but no Agrobacterium DNA or the T-DNA carrying the infectious TYLCTHV clone (*SI Appendix*, Fig. S8*C*). Similar results were obtained from the mechanical transmission assay with CLN1558A plants (*SI Appendix*, Fig. S8 *A* and *B*). PCR quantification of TYLCTHV DNA levels confirmed that virus DNA increased in CLN1558A leaves mechanically inoculated with leaf sap from infected CLN1558A and AVTO2225-S plants, but no significant increase in virus DNA was detected in leaves after inoculation with leaf sap from infected AVTO2225-R and AVTO2226 plants (*SI Appendix*, Fig. S8*D*).

**Fig. 4.**
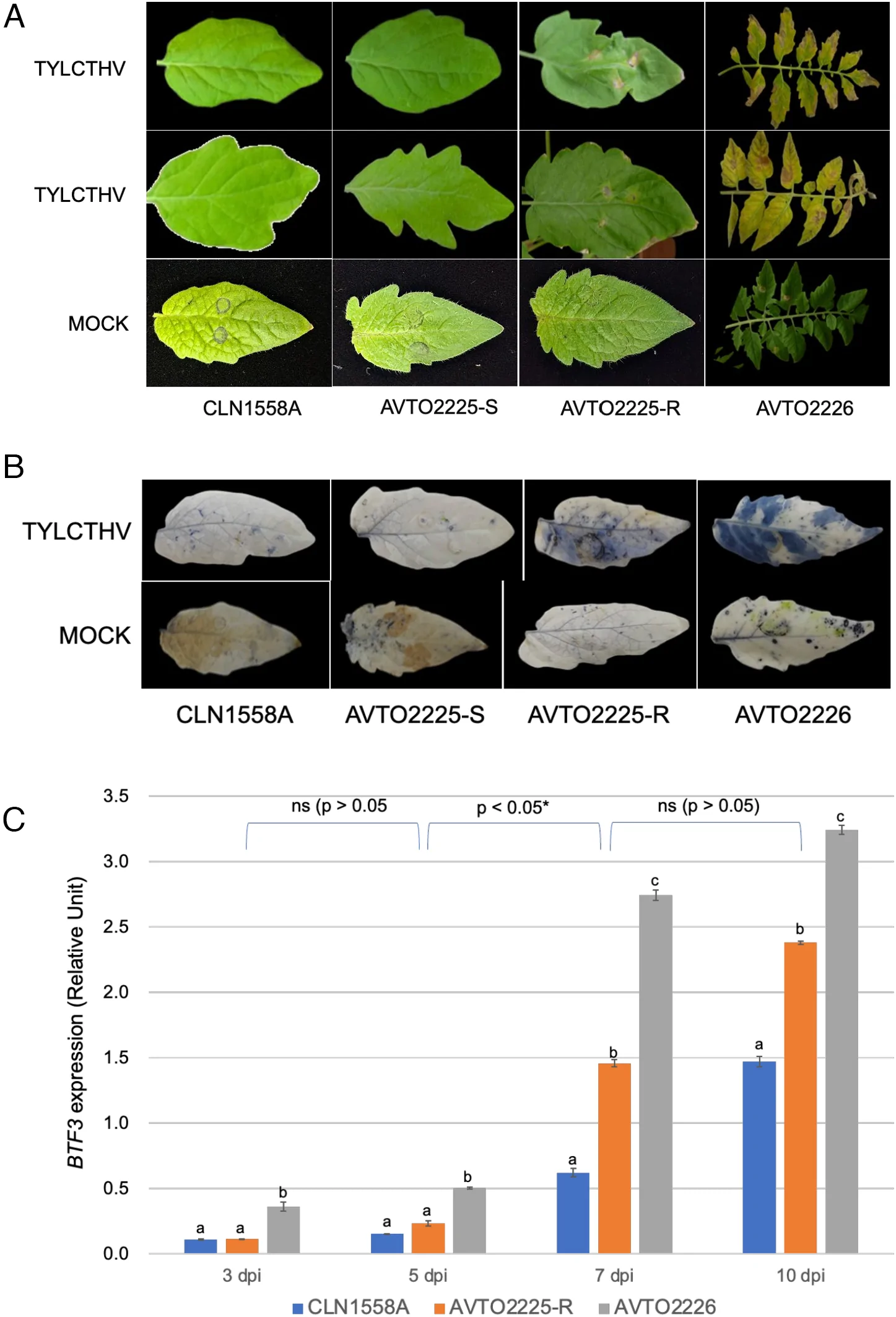
The *POLD1^E622D^* allele restores the HR to TYLCTHV infection in AVTO2225-R and AVTO2226 plants. (*A*) Examples of necrotic lesions resulting from a HR at 7 dpi on AVTO2225-R and AVTO2226 leaves after inoculation with TYLCTHV. Necrotic lesions are not visible on the leaves of CLN1558A and AVTO2225-S plants that are systemically infected with TYLCTHV. (*B*) NBT staining for H_2_O_2_ reveals a stronger ROS response in TYLCTHV-inoculated AVTO2225-R and AVTO2226 leaves than in CLN1558A or AVTO2225-S leaves. (*C*) Increased *BTF3* gene expression at 7 dpi correlates with the HR and increases ROS response in TYLCTHV-inoculated AVTO2225-R and AVTO2226 leaves but is reduced in inoculated CLN1558A leaves. *P* < 0.05* indicates a significant increase in *BTF*3 expression between 5 and 7 dpi. a, b, c = indicate significant differences between plants at *P* < 0.05; similar letters indicate no significant differences.

The begomovirus TrAP protein can act as an avirulence factor, triggering a HR (56, 57). This response can be modulated by other viral proteins (e.g. AC4, AV2), allowing the viral genome to replicate and spread in susceptible plants (58, 59). Seven days after inoculation with TYLCTHV, leaves of CLN1558A and AVTO2225-S plants did not exhibit HR symptoms, whereas HR symptoms were clearly detectable in inoculated leaves of AVTO2225-R plants and even stronger in AVTO2226 plants (Fig. 4 *A* and *B* and *SI Appendix*, Fig. S9 *A*–*D*). This indicates that the *POLD^E622D^* allele is required and sufficient to trigger an HR response to TYLCTHV, which initially replicates and produces mutated viral RNAs and/or proteins in the inoculated leaves. But how these molecules are recognized in the resistant AVTO2225-R and AVTO2226 plants to trigger HR currently remains unknown. Fig. 4*C* shows that the expression of tomato *BASIC TRANSCRIPTION FACTOR 3* (*BTF3*), which regulates the transcription of pathogenesis-related genes during the HR (60), is increased in TYLCTHV-inoculated leaves of AVTO2225-R and AVTO2226 plants at 7 and 10 dpi, but comparatively less in CLN1558A inoculated leaves. The high frequency of mutations detected in the *AC4* and *AV2* genes suggest that their corresponding proteins are also nonfunctional and therefore would not suppress HR in AVTO2225-R and AVTO2226 plants (*SI Appendix*, Fig. S9*E*), provided these proteins have similar functions in TYLCTHV as previously reported for other begomoviruses (58, 59).

To substantiate that the POLD1 mutant enzyme is required and sufficient to cause mutations into the TYLCTHV genome during replication, we transiently expressed cDNAs encoding the CLN1558A POLD1 and AVTO2226 POLD^E622D^ enzymes in *N. benthamiana* and CLN1558A leaves followed by inoculation with the TYLCTHV infectious clone (*SI Appendix*, Fig. S10). The assay revealed that strong infection symptoms developed at 14 dpi in systemic leaves of plants inoculated with the DNA expression vector containing the CLN1558A *POLD1* cDNA or the DNA vector alone (*SI Appendix*, Fig. S10*A*). However, symptoms of systemic leaves from plants inoculated with the DNA vector containing the *POLD1^E622D^* cDNA were comparatively milder (*SI Appendix*, Fig. S10*A*). TYLCTHV DNA could be detected in systemic leaves of all plants transiently expressing the cDNAs after inoculation with the virus (*SI Appendix*, Fig. S10*B*), indicating that even in the presence of the *POLD1^E622D^* allele TYLCTHV DNA can still move. Quantitation of TYLCTHV DNA revealed high levels in the systemic leaves at 14 dpi of plants expressing the CLN1558A *POLD1* cDNA, but DNA levels were significantly reduced in systemic leaves of plants expressing the *POLD1^E622D^* allele (*SI Appendix*, Fig. S10*C*). This indicates that the *POLD1^E622D^* allele, when transiently expressed, has a partial protective effect in *N. benthamiana* where TYLCTHV can activate the host cell *POLD1* genes for its replication. Similar results were obtained for the transient expression assays with CLN1558A plants (*SI Appendix*, Fig. S10 *D*–*F*). Sanger DNA sequencing of *AC1* and *AC2* genes from TYLCTHV DNA isolated from the infected CLN1558A leaves transiently expressing the *POLD1^E622D^* allele revealed high frequencies of mutations, but not from CLN1558A leaves expressing the POLD1 wild type allele (*SI Appendix*, Fig. S10*G*), confirming that POLD1^E622D^ is required and sufficient for introducing mutations in TYLCTHV DNA. CLN1558A and *N. benthamiana* leaves transiently expressing the *POLD1^E622D^* allele also show an increased HR after TYLCTHV infection (*SI Appendix*, Fig. S10*H*), as observed in other experiments.

## Discussion

The DNA replication catastrophe of TYLCTHV after the infection of plants with a mutant POLD1^E622D^ enzyme is a novel and effective resistance mechanism that would be difficult for DNA viruses to evade. Nonsynonymous POLD1 mutations that cause amino acid changes and provide resistance to geminiviruses have independently evolved in cassava (40) and in *S. chilense*. The *S. chilense* mutant *POLD1* alleles from different accessions were then introduced into commercial tomato cultivars for breeding TYLCV-resistant varieties (39). POLD1 mutant alleles can be identified in other agricultural crops as well, for which resistance to geminivirus infections has been reported (61). However, except for the *POLD^E622D^* allele identified here with the mutation in the catalytic domain of the enzyme, the mechanism(s) by which the other reported *POLD1* mutant alleles interfere with geminivirus DNA replication are currently unknown. Modeling of the tomato POLD1^E622D^ and POLD1^Y515H^ mutant proteins suggests that the amino acid substitutions do not alter their three-dimensional structure (Fig. 2*C* and *SI Appendix*, Fig. S5). Since the TYLCTHV genome can initially replicate in AVTO2225-R and AVTO2226 leaves after agroinoculation as well as *N. benthamiana* and CLN1558A leaves transiently expressing the *POLD1^E622D^* allele, the E622D mutation likely does not interfere with the recruitment and assembly of the DNA replication complex at the viral replication origin. Instead, high frequency mutations in the viral genome, as exemplified by *AC1* and *AC2*, are generated by POLD1^E622D^ during rolling-circle DNA replication to produce the single-stranded (ss) viral genome. To date, it has not been examined how the *S. chilense Ty-6 POLD1^Y515H^* mutation (39) and the dominant *POLD1* mutations in the CMD2-resistant farmer-preferred cassava landraces, such as TME3, TME7, or TME204 (40), confer resistance to geminiviruses. It is therefore unknown if they have a similar mutagenicity as POLD1^E622D^ during viral DNA replication or block virus infection and movement by other mechanisms.

Plant DNA polymerase α (POLA) and POLD1 are multisubunit enzyme complexes that mediate the replication of the geminivirus circular ssDNA genome in the nuclei of infected plants via double-stranded (ds) DNA intermediates (46). Rep interacts with the viral replication origin and host proteins, which is essential for efficient DNA replication (62). However, the mechanism by which Rep assembles the multisubunit host replication complex at the viral replication origin is still largely unknown. The POLA primase subunits PRIM1 and PRIM2 are also necessary for converting the ssDNA geminivirus genome into a dsDNA replication intermediate, as was recently shown for TYLCV (63). REn interacts with Rep, enhancing viral replication and infection efficiency via an unknown mechanism (64). Following agroinoculation of AVTO2225-R and AVTO2226 plants with TYLCTHV, the Rep protein, which is initially produced from the intact viral genome, can reprogram transcription in the host cell (65). This includes the activation of both alleles of the wild-type and POLD1^E622D^ enzymes, which assemble in the host DNA replication complex on the viral replication origin. However, our data suggest that after several rounds of TYLCTHV rolling-circle DNA replication, mutations accumulate in the resulting ssDNA genome introduced by POLD1^E622D^ in a DNA replication complex. Even if replication complexes could be assembled on new ssDNA mutant viral genomes, a replication complex containing a wild-type POLD1 would perpetuate mutations introduced by the POLD1^E622D^ enzyme in previous DNA replication cycles. Mutated viral ssDNA molecules may also be able to move to neighboring cells. However, given the numerous nucleotide changes and INDELs detected in the TYLCTHV *AC1* gene sequences after infection (Fig. 3*A* and *SI Appendix*, Fig. S7), it is unlikely that a mutant Rep protein (Fig. 3*B*), if translated, would still be functional for transcriptional reprogramming and viral replication in a new host cell.

TrAP is a multifunctional protein that acts both as a pathogenicity factor and a transcriptional activator to promote transcription by RNA polymerase II (66) as well as the activation of the viral coat protein gene promoter (67). TrAP suppresses transcriptional and posttranscriptional gene silencing (PTGS) by altering the expression of host genes involved in gene silencing through the interaction with JAZ8, a JASMONATE ZIM-DOMAIN protein that localizes to the nucleus together with TrAP (68), and by inhibiting host cell DNA methylation (69). Given the high frequency nucleotide changes and INDELs identified in TYLCTHV *AC2* gene sequences following AVTO2225-R and AVTO2226 leaf infections (Fig. 3*C* and *SI Appendix*, Fig. S7), it is improbable that a mutant TrAP protein could function to disrupt the host immune response. This scenario is consistent with the HR to the mutated TYLCTHV DNA that accumulates in AVTO2225-R and AVTO2226 plants (Fig. 4), as well as the high frequency of mutations in the TYLCTHV *AC4* and *AV2* genes (*SI Appendix*, Fig. S9*E*) whose proteins function as DNA methylation and HR suppressors in other begomoviruses (58, 59). It is also possible that the *Ty-1* resistance allele that is present in some of the AVTO2225-R and AVTO-2226 genotypes and the *Ty-2* resistance allele that is present in all AVTO2225-R and AVTO2226 lines contribute to HR in the presence, but not in the absence of the *Ty-6* allele.

Wild crop relatives often serve as excellent sources of resistance to pathogens because they exhibit greater genetic variation than domesticated crops, which have experienced genetic bottlenecks during domestication and selection processes (70). These wild crop relatives also coevolve with native pathogens, developing robust natural resistance mechanisms, which can be utilized for crop improvement (71). *S. chilense* has a wide geographic distribution in various environments in southern Peru and northern Chile, with genetically and phenotypically diverse populations (72). Some of these populations have coevolved natural resistance with local begomoviruses and their *B. tabaci* vectors. Accessions from coastal *S. chilense* populations have strong TYLCV resistance. For instance, the *Ty-1* and *Ty-3 RDR* resistance alleles found in these accessions have been widely utilized in tomato breeding (26). The two known *Ty-6* resistance alleles, *POLD1^E622D^* (this study) and *POLD1^Y515H^* (39), likely evolved independently in different *S. chilense* populations experiencing strong local TYLCV pressure. This situation is similar to that of cassava, where farmers over 30 years ago identified spontaneous resistant plants in otherwise CMD-infected fields. These plants were found to have different dominant mutant *POLD1* alleles associated with resistance (40). The *S. chilense POLD1^E622D^* mutant allele is likely maintained in local populations because it robustly protects against infection with even severe tomato begomovirus strains, although the *Ty-6 POLD1^E622D^* allele can only exist in a heterozygous state (Fig. 2). In a selfed F1 population, a certain percentage of *S. chilense* plants lost the *Ty-6 POLD1^E622D^* allele (*SI Appendix*, Fig. S4), but these plants likely retain other *Ty* genes that provide at least partial resistance to TYLCV infection (*SI Appendix*, Table S1). However, since *S. chilense* is an obligate outcrosser that does not easily self-fertilize (73), the heterozygous *Ty-6 POLD1^E622D^* allele is expected to remain prevalent in local breeding populations. Furthermore, the presence of the *Ty-6 POLD1^E622D^* allele would significantly reduce the pressure in a quasispecies TYLCV population to overcome the protection provided by other *Ty* resistance genes. Due to the high frequency of nucleotide changes and INDELs introduced by POLD1^E622D^ in the viral genome, the evolution of new TYLCV strains resistant to *Ty* genes in the context of the *Ty-6* resistance is improbable. It remains to be seen if and how the POLD1^E622D^ enzyme affects the nuclear genome during DNA replication in *S. chilense* and after its introduction into commercial tomato cultivars. Yeast models have been widely used to test whether mutant Pol δ causes elevated mutation rates (74). Mutations in the catalytic activity, proofreading function, and interaction with other replication proteins in human and animal POLD1 have been associated with reduced genome stability and various diseases, including cancer (75). The time at which the heterozygous *POLD1^E622D^* mutant allele evolved in *S. chilense* and how long it has been maintained in the population are currently unknown. It is likely that the wild-type POLD1 can correct nucleotide changes introduced by POLD1^E622D^ during genome DNA replication through extrinsic proofreading (76) or mismatch repair (77), for example. In this case, POLD1^E622D^ mutagenicity for the host genome DNA replication would be mitigated. The results from our study indicate that such DNA proofreading and repair mechanisms are not active or effective during the rolling circle DNA replication of TYLCTHV, thus making *POLD1^E622D^* a novel and effective begomovirus resistance gene.

## Materials and Methods

### Plant Material.

This research used *S. chilense* accession LA2779 (AVTO2226) maintained at the World Vegetable Center (WVC, Tainan, Taiwan) that carries a *Ty-6* resistance allele, *S. lycopersicum* AVTO2225 (a TYLCV resistant breeding line provided by WVC), and *S. lycopersicum* CLN1558A (*SI Appendix*, Fig. S11). WVC provided 20 *S. chilense* AVTO2226 (hereafter AVTO2226) and 20 *S. lycopersicum* AVTO2225 (hereafter AVTO2225) seedlings. Based on information from WVC, AVTO2225 has a genotype background of the commercially available ANSAL tomato hybrid (Seminis Vegetable Seeds, Oxnard, CA). AVTO2225 was derived via the 220407-6 breeding female parent and the BL1972F2-92-7-6 pedigree (22AU0865-8; *SI Appendix*, Fig. S12). According to the WVC, AVTO2225 contains a *Ty-6* resistance allele introgressed from *S. chilense* LA2779 (AVTO2226), but the status of other *Ty* resistance alleles was uncertain (*SI Appendix*, Fig. S12). The AVTO2225 and AVTO2226 seedlings were initially randomly labeled by WVC and relabeled for the purpose of this study (*SI Appendix*, Table S2). *S. lycopersicum* CLN1558A (hereafter CLN1558A) is a virus-susceptible Taiwan tomato variety. Seeds were provided by Tsai Wen-Hsin (Department of Plant Medicine, National Chiayi University, Taiwan). The tomato plants were grown in an insect-free greenhouse. AVTO2225 and CLN1558A plants were separated from AVTO2226 plants to avoid potential cross-fertilization. As a precaution, all plants were treated weekly with insecticides containing the active ingredient 3-(2-chloro-1,3-thiazol-5-ylmethyl)-5-methyl-1,3,5-oxadiazinan-4-ylidene(nitro)amine (Ramcides CropScience Pvt. Ltd., Puddukkotai, India).

### Plant Multiplication, Propagation, and Genetic Crosses.

To expand the original 20 AVTO2225 and 20 AVTO2226 plants obtained from the WVC for virus inoculation experiments, the plants were clonally propagated. The upper shoots were cut into segments, dipped into an auxin solution containing 1 mg/l indole-3-acetic acid, and planted in moist soil mixed with NPK 14-16-18 fertilizer (BVB SUBSTRATES, De Lier, The Netherlands). This process was repeated to obtain several clonal plants from each original plant. These clonal plants were grown until they reached the three-leaf stage, at which point they were used for the virus inoculation experiments. The remaining shoots of the original plants were grown to maturity for fruit production. Since *S. chilense* is an obligate outcrosser that does not readily self-fertilize (73), the pollen from the AVTO2226 plants was collected overnight and used for assisted self-fertilization. Eight of the 20 AVTO2226 plants produced fruit, and four of these plants had enough seeds for subsequent analysis of F1 plants (*SI Appendix*, Table S3). All CLN1558A plants were grown from seeds.

### DNA and RNA Extraction.

DNA and RNA were extracted using the methods described in ref. 78. The isolated DNA was used for genotyping of the *Ty* markers as well as TYLCTHV detection and DNA quantification of infected plants. RNA extraction was carried out with some modifications. One µL of DNAase (Promega, Madison, WI) was added to the RNA solution, which was then incubated at 37 °C for 1 to 2 h. The RNA was precipitated with 100% ethanol at −80 °C for 30 min and resuspended in 45 µL distilled water.

### Genotyping and Sequencing of *Ty* Markers.

All DNA primers that were used for genotyping are listed in *SI Appendix*, Table S4. The 20 AVTO2225 and 20 AVTO2226 plants obtained from WVC were first genotyped because their status for the *Ty-1*, *Ty-2,* and *Ty-6* alleles was uncertain. Genomic DNA was used for PCR with published DNA primers for the *Ty-1* CAPS, *Ty-2* SCAR, and *Ty-6* CAPS markers [(26, 30, 38); *SI Appendix*, Table S4]. The resulting PCR products were separated by agarose gel electrophoresis. The DNA bands in the agarose gels were excised, placed into 1.5 mL Eppendorf tubes, and purified using a miniprep DNA cleanup kit (GeneMark, Taiwan). The DNA fragments were cloned into the cloning vector using the TOPO TA kit (Invitrogen, Thermo Fisher, Taiwan) following the protocol described by ref. 79.

The DNA vector constructs were transformed into *Escherichia coli* DH5α and plated on Luria-Bertani (LB) broth containing 1.5% agar and 50 μg/mL kanamycin (Bio Basic, New York). Plates were incubated at 37 °C overnight. Bacterial colonies were picked and cultured overnight in LB medium containing 50 μg/mL kanamycin. Plasmids were extracted using a miniprep plasmid extraction kit (GeneMark, Taipei, Taiwan) and used for Sanger DNA sequencing at the National Chung Hsing University (NCHU) Biotechnology Center. The DNA sequences were analyzed using BLAST (National Center for Biotechnology Information, U.S.A.).

### TYLCTHV Infection Assays.

TYLCTHV was first isolated from tomato fields in Taiwan (48, 80). An infectious clone of the bipartite TYLCTHV genome consisting of the DNA-A and DNA-B segments was prepared as described (81). Leaf inoculations were performed by agroinfiltration with the infectious TYLCTHV clone that consisted of two *Agrobacterium tumefaciens* GV3101 strains transfected with either the DNA-A segment or the DNA-B segment of the TYLCTHV bipartite genome. The Agrobacterium strains were cultured overnight at 28 °C in 5 mL of LB media supplemented with the appropriate 50 mg/mL kanamycin. Unless otherwise indicated, acetosyringone (Bio Basic, New York) was added to the Agrobacterium cultures. The original cultures were subcultured by transferring 1 mL of the overnight Agrobacterium culture to fresh LB medium supplemented with antibiotics. The Agrobacterium subcultures were incubated at 28 °C until the density reached an OD_600_ of 1.0 (Multiskan FC, Thermo Fisher Scientific, MA) and then centrifuged at 6,000 rpm using a JA25.5 rotor (Beckman Coulter, CA). The pellets were resuspended in 10 mL agroinfiltration buffer (82). The Agrobacterium suspensions were kept in the dark for 2 h at room temperature before equal aliquots of the two suspensions were mixed and injected into leaves using a syringe without a needle (TOP, Kaohsiung, Taiwan). Samples from systemic or inoculated leaves were collected at various time points for detection or quantification of TYLCTHV DNA.

### Mechanical TYLCTHV Inoculation.

Leaf saps for mechanical inoculation were extracted at 7 dpi from 2 gm of CLN1558A, AVTO2225-S, AVTO2225-R, and AVTO2226 leaves that were inoculated with Agrobacteria containing an TYLCTHV infectious clone. The leaf samples were ground in 20 mL of 10 mM potassium phosphate buffer solution (pH 7.0). Leaf saps were analyzed for the presence of the Agrobacterium T-DNA plasmid using the *Kan^R^* gene as a marker, the T-DNA carrying the TYLCTHV infectious clones using the *Hyg^R^* gene as a marker, and TYLCTHV DNA. *N. benthamiana* and CLN1558A leaves were rubbed with 0.5 mL of the leaf saps containing the virus inoculant and carborundum powder (400 to 500 mesh, Tosa Emery, Kochi, Japan). After 30 s, the mechanically inoculated leaves were washed with distilled water. Segments of leaflets of the inoculated CLN1558A leaves were collected by placing the leaflet between the cap and tube of an Eppendorf 1.5 mL tube and closing the lid to capture the leaf segment, which was immediately frozen in liquid nitrogen. Leaf segments were collected at 9, 11, 13, 15, 17, 19, and 21 dpi. DNA was extracted from the leaf segments and analyzed for TYLCTHV DNA levels using qPCR and the tomato *ACTIN* gene (GenBank accession BT013707) for normalization.

### PCR Detection of TYLCTHV Infection and Sanger Sequencing of Viral Genes.

TYLCTHV infection was detected by PCR using DNA extracted from leaves and TYLCTHV-specific primers (*SI Appendix*, Table S4). The PCR reaction mixture contained 0.25 U ProTaq-polymerase (PROTECH, Taipei, Taiwan) and 0.05 mM dNTPs (PROTECH, Taipei, Taiwan) in a total volume of 50 µL. DNA extracted from uninfected tomato leaves was used as a negative control. The PCR cycles were run at 95 °C for 3 min, followed by 40 cycles of 95 °C for 10 s and 58 °C for 30 s using a LifePro PCR instrument (Hangzhou, China). The tomato *PHYTOENE DESATURASE* gene (GenBank accession DQ339100) was used as a PCR internal control.

The viral *AC1*, *AC2*, *AC4,* and *AV2* genes were amplified by PCR with DNA isolated from infected leaves at 7 dpi using specific primers (*SI Appendix*, Table S4). The PCR fragments were cloned using the TOPO TA kit (Invitrogen, Thermo Fisher, Taiwan) following the protocol described by ref. 79. At least three independent clones of *AC1* and *AC2* genes from 12 plants of each CLN1558A, AVTO2225, and AVTO2226 were sequenced using the Sanger method. Independent clones of *AC*4 and *AV2* genes were also sequenced from CLN1558A, AVTO2225, and AVTO2226 plants.

### TYLCTHV Infection Symptom Scoring.

Symptoms on susceptible tomato plants were visible at 30 dpi when symptom scoring was performed on a scale of 0 to 4 as described by ref. 83: 0 = no symptoms; 1 = slight leaf yellowing (mild symptoms); 2 = leaf yellowing and curling (moderate symptoms); 3 = leaf yellowing, curling, and cupping (severe symptoms); 4 = severe stunting, leaf curling, cupping, and plant growth arrest (very severe symptoms). Symptom scores were obtained for 12 plants of each AVTO2225, AVTO2226, and CLN1558A in two independent infection experiments, with three replications in the second infection experiment. For statistical analysis, symptom scores were transformed to a scale of 1 to 5 and the significance of the data was evaluated using the two-sided Mann–Whitney *U* statistical test.

### TYLCTHV DNA Quantification.

Samples of leaf segments inoculated with the virus were collected at 3, 7, 14, and 21 dpi from plants 1 to 12 of CLN1558A, AVTO2225, and AVTO2226 (*SI Appendix*, Table S3). DNA was extracted and analyzed for TYLCTHV DNA accumulation using qPCR, as described by ref. 78, with DNA primers specific for TYLCTHV (*SI Appendix*, Table S4). The tomato *ACTIN* gene (GenBank accession BT013707) was used for normalization.

### *POLD1* Amplification and Sanger DNA Sequencing.

*POLD1* cDNA gene amplification was carried out using RNA extracted from AVTO2225, AVTO2226, and CLN1558A leaf samples. To simplify the amplification process, the 3 kb *POLD1* cDNA was amplified in four DNA segments (1,180 bp, 520 bp, 833 bp, and 725 bp) using specific primers (*SI Appendix*, Table S4) and cloned into the TOPO-TA vector. The *POLD1* cDNA fragments were sequenced using Sanger DNA sequencing at the NCHU Biotechnology Center. DNA sequence alignment was conducted using the CLUSTALW multiple sequence alignment program. Following base calling, the Sanger DNA sequence traces were manually inspected for double nucleotide peaks, indicating heterozygous nucleotide positions.

### *BTF3* Gene Expression Analysis.

Reverse transcription quantitative PCR (RT-qPCR) was performed according to the method described by ref. 78 to analyze *BTF3* gene expression. RNA was extracted from agroinfiltrated leaves at 3, 5, 7, and 10 dpi. RT-qPCR was performed using an iQ-SYBR Green Supermix reagent kit (Bio-Rad Laboratories, Taiwan) in a CFX Connect Real-Time System (Bio-Rad Laboratories, Taiwan). Tomato *ACTIN* gene expression (GenBank accession BT013707) was used for normalization.

### Necrotic Lesion and Reactive Oxygen Species (ROS) Quantification.

Necrotic lesions on leaves were counted manually. Hydrogen peroxide (H_2_O_2_) accumulation in TYLCTHV-inoculated leaves was detected by histochemical staining with nitroblue tetrazolium (NBT) as described by ref. 84. Leaves collected at 7 dpi were soaked overnight in a solution containing 0.1% (w/v) NBT (PROTECH, Taipei, Taiwan) in 50 mM phosphate-buffered saline (PB) buffer (pH 7.5) in the dark. Chlorophyll was removed by incubating the leaves overnight in 70% ethanol at room temperature. The stained leaves were photographed using a Nikon D750 Fx digital camera.

### Transient *POLD1* and *POLD1^E622^*^D^ Expression for TYLCTHV Replication Assays.

Full-length cDNAs of CLN1558A *POLD1* and AVTO2226 *POLD1^E622D^* were amplified by PCR using specific primers (*SI Appendix*, Table S4). The amplified cDNAs were fused to a FLAG-tag at their C termini using specific primers (*SI Appendix*, Table S4). The cDNAs were cloned into the Gateway entry vector pENTR/D-TOPO (Invitrogen, Thermo Fisher, Taiwan). The pENTR/D-TOPO vectors containing the *POLD1* WT and *POLD1^E622D^* cDNAs were recombined into the pK2GW7 Gateway expression vector as described (84) to generate transient gene expression plasmids. The plasmids were transformed into *A. tumefaciens* GV3101. The pK2GW7-*POLD1* and pK2GW7-*POLD1^E622D^* DNA constructs were delivered to *N. benthamiana* and CLN1558A leaves via agroinfiltration. After 48 h, the infiltrated leaves were inoculated with a TYLCTHV infectious clone by agroinfiltration. Systemic leaves of *N. benthamiana* were collected at 14 dpi, and systemic leaves CLN1558A at 30 dpi. The leaf samples were used for DNA extraction and quantification of TYLCTHV DNA levels using qPCR. The tomato *ACTIN* gene (GenBank accession BT013707) was used for normalization.

### AlphaFold3 Modeling.

The POLD1 protein structure predictions of AVTO2225, the AVTO2225-R E622D mutant, and AVTO2226 were carried out using the AlphaFold server, employing the AlphaFold3 algorithm with default parameters and with the random seed set to 100.

While AlphaFold3 has greatly improved protein structure prediction, it can still be limited in accurately modeling the structural effects of single amino acid mutations. To further assess the impact of the E622D mutation and other amino acid variants between the POLD1 proteins encoded in the AVTO2225-S, AVTO2225-R, and AVTO2226 genomes, we employed additional structure-based computational tools, including DynaMut2 (85) and DDMut (86), which calculate changes in free energy (ΔΔG) upon mutation for protein stability and potential structural alterations estimation. Across all these methods with their default parameters, the results indicated that the POLD1 E622D mutation and other amino acid variants between AVTO2225 and AVTO2226 had minimal or no effect on the predicted overall protein structure.

### Statistical Analysis.

Unless otherwise noted, each sample in the experiments described in this article had at least three replications. All data except the TYLCTHV symptom score data were analyzed using ANOVA Single Factor tests, and statistical significance was calculated using Microsoft Excel. A difference was considered statistically significant at *P* < 0.05.

## Supplementary Material

Appendix 01 (PDF)

## Data Availability

Study data are included in the article, *SI Appendix*, and in the Open Science Framework (https://osf.io) repository (87).
