# Supplementary material for: A dominant mutation in tomato DNA POLYMERASE DELTA 1 causes geminivirus DNA replication catastrophe
Source: Proc Natl Acad Sci U S A. 2026 Aug 24;123(35):e2531841123. doi: 10.1073/pnas.2531841123 (PMC13535188; doi:10.1073/pnas.2531841123)
Supplement: Supplementary file 1 [file pnas.2531841123.sapp.pdf]

Supplementary Data

A dominant mutation in tomato DNA POLYMERASE DELTA 1 causes geminivirus DNA replication catastrophe

Deri Gustian, Chen-Hsin Yu, Fuh-Jyh Jan and Wilhelm Gruissem

Supplementary Figures 1 to 10 and Supplementary Table1 provided in this Supplementary Material are discussed in more detail in the Results section of the manuscript. Supplementary Figures 11 and 12, and Supplementary Tables 2, 3 and 4, are described in more detail in the Methods section of the manuscript.

a

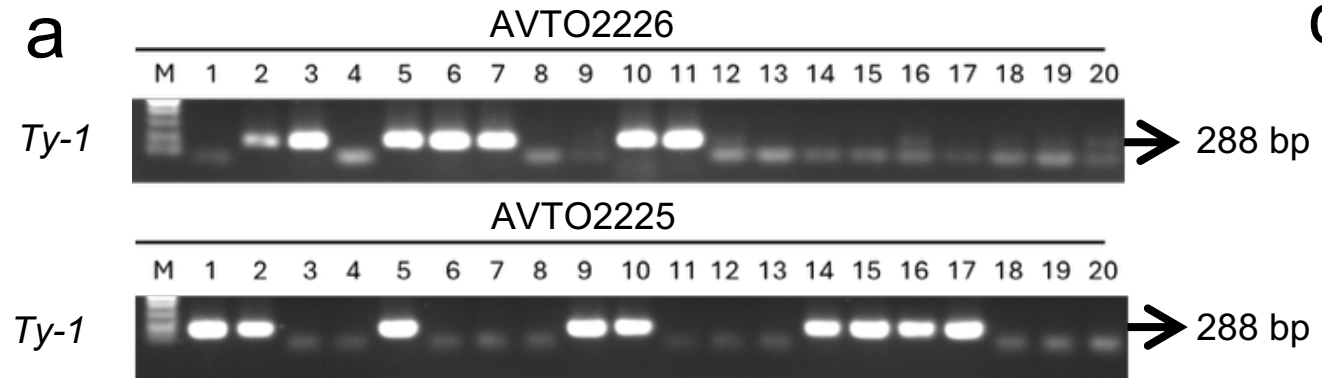

c

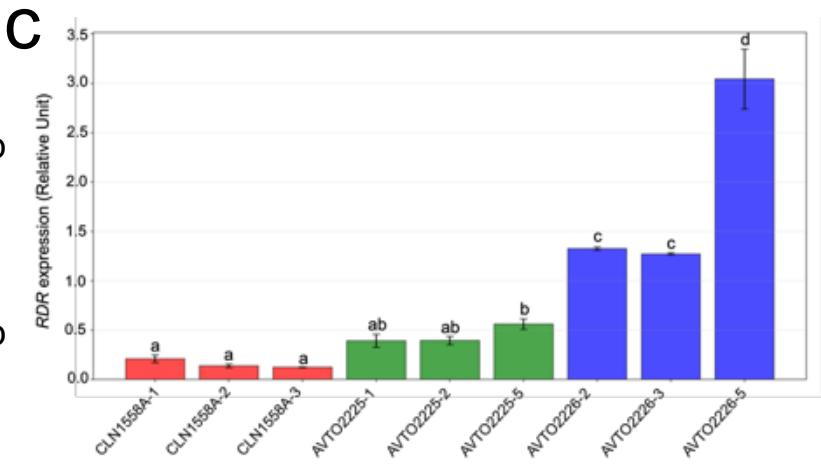

b

CATGTAAACAAGAAAAGCAGAGAGACTCTTCTTGATAGGACGACGTGATATTATTTTGAGCATTTCTAATGACCCTTT
TTCACCAATAGAGCTCAGCCTCCTTCTAATGCCAGTACACGGTGGTTTTTGCCCCTGCTCCTTGCAGATTCTATCAA
GCATCGTCTCTACAGAATATGGTAAAGGTGCATCCAGAACA

Description	Scientific Name	Max Score	Total Score	Query Cover	E value	Per. ident	Acc. Len	Accession
PREDICTED: Solanum lycopersicum RNA-dependent RNA polymerase (TY-1/TY-3), mRNA	Solanum lycopersicum	490	490	94%	5.00E-134	100	3129	XM_010323869.4
Solanum lycopersicum cultivar Ty1/3 genotype SL 105 RNA-dependent RNA polymerase 3 (RdRp) gene, partial cds	Solanum lycopersicum	466	466	89%	8.00E-127	100	252	OR762176.1
Solanum cheesmaniae LA0166 DNA, chromosome 6, nearly complete sequence	Solanum cheesmaniae	484	484	94%	2.00E-132	99.62	51870238	AP038855.1
PREDICTED: Solanum pennellii probable RNA-dependent RNA polymerase 3 (LOC107021779), mRNA	Solanum pennellii	420	420	83%	7.00E-113	99.14	3110	XM_015222485.1
Solanum lycopersicum cultivar Ty1/3 genotype SL 159 (AVTO1315) (CLN3241Q) RNA-dependent RNA polymerase (RdRp) gene, partial cds	Solanum lycopersicum	405	405	80%	2.00E-108	99.11	264	OR762175.1
PREDICTED: Solanum tuberosum probable RNA-dependent RNA polymerase 5 (LOC102579148), transcript variant X1, mRNA	Solanum tuberosum	322	322	79%	2.00E-83	92.79	3215	XM_006338799.2
PREDICTED: Solanum tuberosum probable RNA-dependent RNA polymerase 5 (LOC102579148), transcript variant X2, mRNA	Solanum tuberosum	322	322	79%	2.00E-83	92.79	2632	XM_015308307.1
PREDICTED: Solanum stenotomum probable RNA-dependent RNA polymerase 3 (LOC125874389), mRNA	Solanum stenotomum	322	322	79%	2.00E-83	92.79	3118	XM_049555268.1
PREDICTED: Solanum verrucosum probable RNA-dependent RNA polymerase 3 (LOC125819273), mRNA	Solanum verrucosum	316	316	79%	9.00E-82	92.34	3095	XM_049498741.1

d

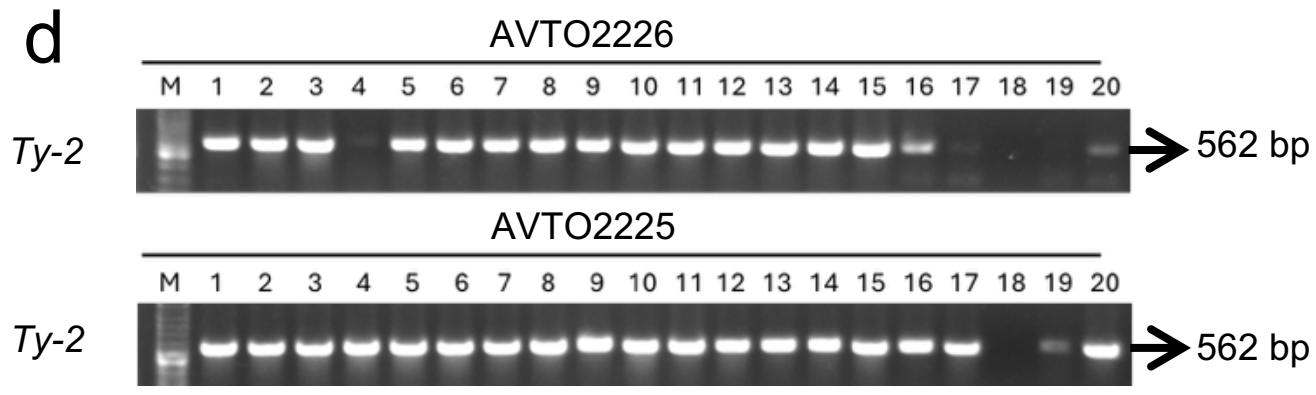

f

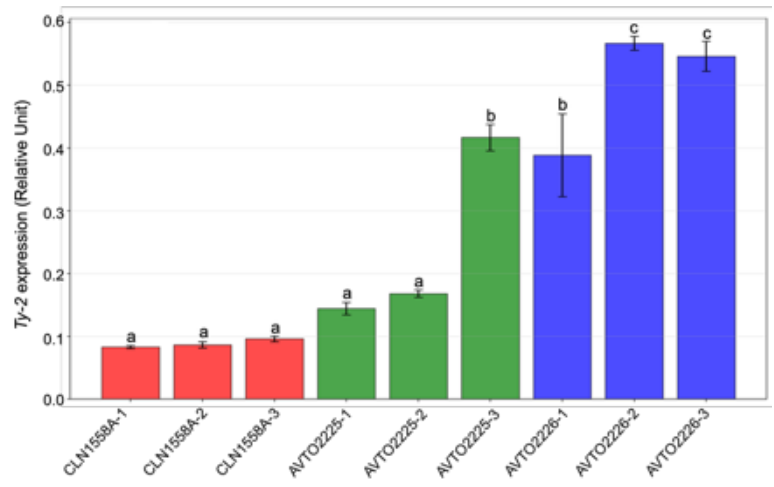

e

GCTCTGAACTGTGACTCAGGTATTATGTGATTTTTTCGGAGGCTGTGTTTTCTTTGATTTGATGATTGCGGATGCATCCAT
TTTCATTCTGAGCTCTTAGGTCCTTCCCTTGACAATTTCTTGTTAATATTCAAGAAAATATGAGAACATTAGCACAATCTAG
GCATCTAGAAACTTGTATTAGAGATTTAAACCATATTTGCGAAGACTTTGGTCCCACTAGAGGGGGTGTGCAGCATACAG
TAATCTGACCCAAAGATTGAGAGAGCAAGTGTCAGCAAGGGAGAGAGAAGTGCAAAAGAGAACATCACATCAAAGGGCG

Description	Scientific Name	Max Score	Total Score	Query Cover	E value	Per. ident	Acc. Len	Accession
Solanum chilense cultivar LA2779 marker TG0302 genomic sequence	Solanum chilense	749	749	90%	0	98.81	739	KF887338.1
Solanum chilense isolate LA2779 T0302 RFLP marker genomic sequence	Solanum chilense	791	791	96%	0	98.44	789	EU046612.1
Solanum lycopersicum cultivar Gh13 marker TG0302 genomic sequence	Solanum lycopersicum	721	721	90%	0	97.63	742	KF887341.1
Solanum lycopersicum isolate M82-1-8 T0302 RFLP marker genomic sequence	Solanum lycopersicum	763	763	96%	0	97.34	792	EU046613.1
Solanum cheesmaniae LA0166 DNA, chromosome 11, nearly complete sequence	Solanum cheesmaniae	743	743	96%	0	96.47	61380607	AP038860.1
Solanum lycopersicum isolate breeding line H24 T0302 RFLP marker genomic sequence	Solanum lycopersicum	448	848	96%	5.00E-121	94.83	909	EU046610.1
Solanum habrochaites cultivar LA1777 marker TG0302 genomic sequence	Solanum habrochaites	448	801	91%	5.00E-121	94.83	861	KF887340.1
Solanum habrochaites cultivar La0386 marker TG0302 genomic sequence	Solanum habrochaites	448	801	91%	5.00E-121	94.83	861	KF887339.1

g

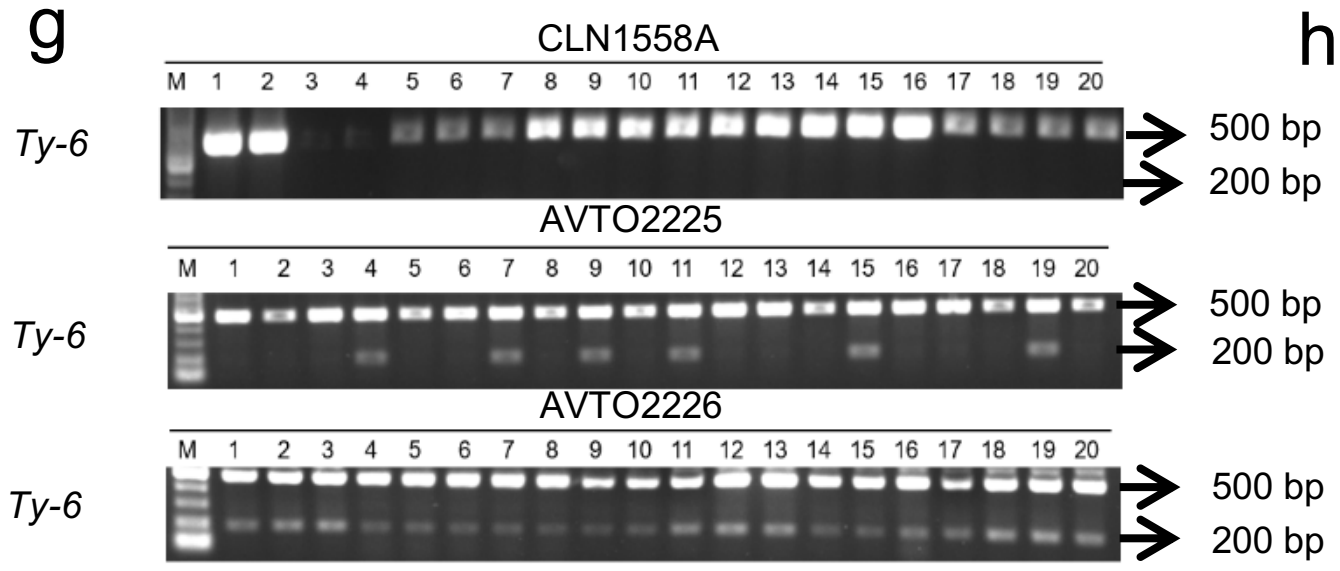

i

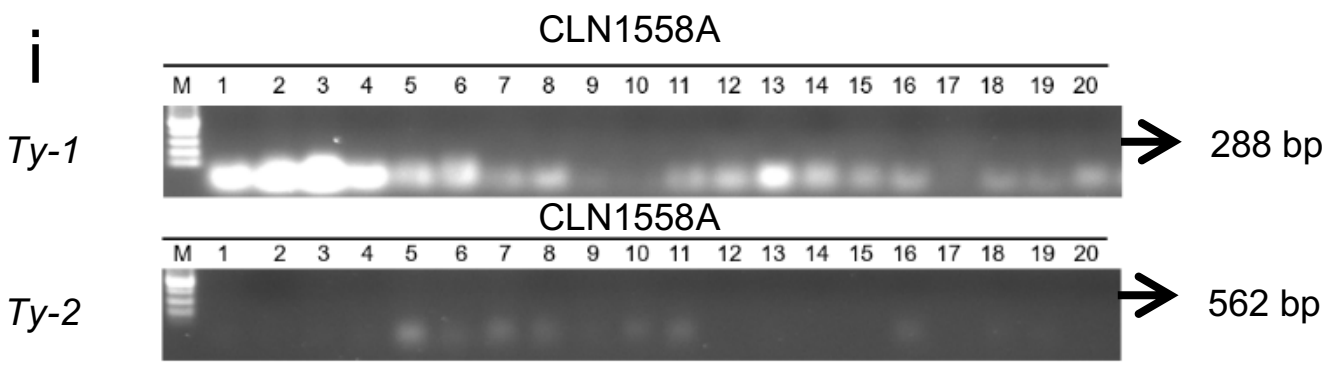

h

6.1
GACGAATTCGTCGATTCAGCGGTTTAGGA
GGATGATGAGGTTTACGTCGGGCCGGAGA
TCGGAATCCTGGGCCGCCGATGAGATCGA
TGCAGTGCTTTCAGAGTTGGTACAGATCG
GAAAGTGGGTCCCAATATAAGAAATTGAAT
TCCAGTGAAGGAGCGCGCGGAAGTCATTT
CTATGATGGTTAAAATTTCGTTAATCCAAC
GTGGCAGTAAAATTTACTCAATATTTCTCTA
GTTGAGCAATTTCTTTTTTCAATTTTTATTG
GTTAACTATTATGCGAGAATTATAATGTAAA
TTTAAATGTATCACTCTCGAAAAAAAATCTA
TTAATTCTCTTTTATGAACATAAACGTAACG
ATATCATAGTAAGGTTTATGGTTCAAAATC
ATAATAGAAGAAATATTTTCTACTTCGTACA
TACT

6.2
GAAGTTTTGATTACAGAAGCAGAAGAAGA
ATTAGTAGCAGGGTATCAAACCGAATATT
CTGGTATCAAATTTGGTTTATTTTACATTG
CTTCATATCTGAATCTACTAGTTTCTTCAT
TATTTGTAACAGTTCTTCACTTGGTTTGGA
ATGGAATCGAGGTC

Description	Scientific Name	Max Score	Total Score	Query Cover	E value	Per. ident	Acc. Len	Accession
Solanum cheesmaniae LA0166 DNA, chromosome 10, nearly complete sequence	Solanum cheesmaniae	725	725	98%	0	96.61	65485824	AP038859.1
PREDICTED: Solanum pennellii uncharacterized LOC107032519 (LOC107032519), mRNA	Solanum pennellii	627	627	91%	6.00E-175	94.42	1283	XM_015234122.2
PREDICTED: Solanum lycopersicum disease resistance protein Ty-6 (TY-6), mRNA	Solanum lycopersicum	582	582	77%	1.00E-161	97.13	1224	XM_004249390.5
Solanum lycopersicum cDNA, clone: LEFL1014BG03, HTC in leaf	Solanum lycopersicum	577	577	75%	6.00E-160	97.37	1196	AK320906.1
PREDICTED: Solanum stenotomum uncharacterized LOC125866280 (LOC125866280), mRNA	Solanum stenotomum	370	370	75%	1.00E-97	86.25	1314	XM_049546617.1
PREDICTED: Solanum verrucosum uncharacterized LOC125807095 (LOC125807095), mRNA	Solanum verrucosum	355	355	74%	3.00E-93	85.83	1204	XM_049486864.1
PREDICTED: Solanum dulcamara uncharacterized LOC129876339 (LOC129876339), mRNA	Solanum dulcamara	239	239	37%	3.00E-58	92.81	1225	XM_055951749.1
PREDICTED: Solanum tuberosum uncharacterized protein (LOC107061365), mRNA	Solanum tuberosum	220	220	30%	1.00E-52	96.32	918	XM_015310516.1

j

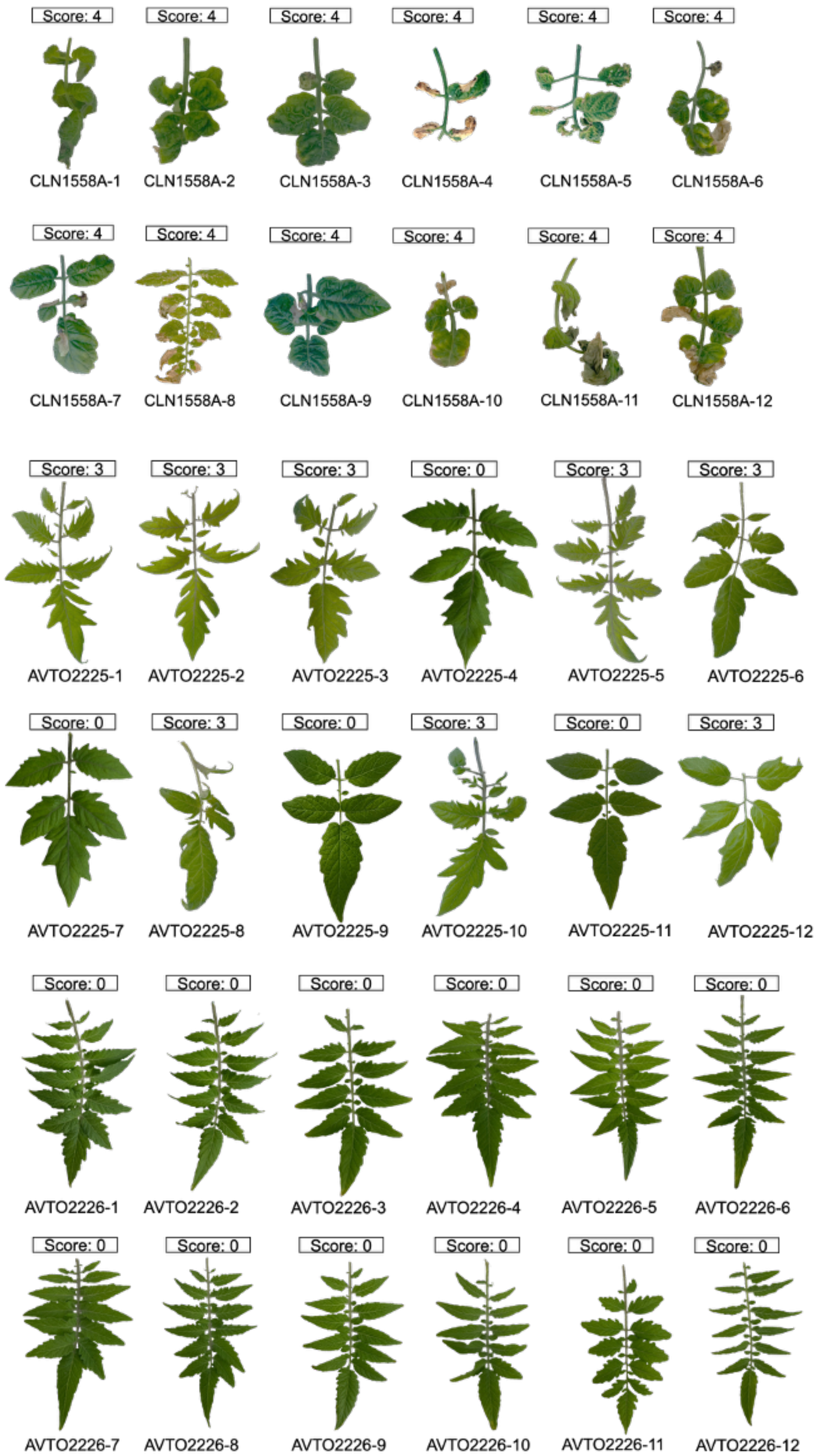

Tomato lines	Median symptom score*	Mean
CLN1558A	5^{a}	5
AVTO2225	4^{b}	3
AVTO2226	1^{c}	1

*Score scale 0-4 was transformed to 1-5 for statistical testing

Figure S1

The presence of *Ty-1*, *Ty-2* and *Ty-6* resistance alleles in CLN1558A, AVTO2225 and AVTO2226 plants and TYLCTHV infection assay (see Materials and Methods for details). **a**. The PCR results show that the AVTO2225 and AVTO2226 plants are from a mixed population that carries the *Ty-1* resistance allele of the *RDR* gene (1,2), as determined by the 288 bp *Ty-1* marker PCR product. **b**. Sanger DNA sequencing and BLAST analyses confirm that the 288 bp *Ty-1* CAPS marker PCR product is derived from the *RDR Ty-1* allele. **c**. The relative expression of *RDR* is shown in three randomly selected infected CLN1558A plants, as well as in three randomly selected infected AVTO2225 and AVTO2226 plants each that carry the *Ty-1* allele. a,b,c = indicate significance at $p < 0.05$. **d**. PCR results reveal that most AVTO2225 and AVTO2226 plants carry the 562 bp *Ty-2* co-dominant SCAR marker PCR product associated with the *NBS-LRR* gene (3), which confers resistance to begomovirus infections. **e**. Sanger DNA sequencing and BLAST analyses confirm that the 562 bp PCR product represents the *Ty-2* SCAR marker associated with the *NBS-LRR* gene. **f**. Relative expression of the *NBS-LRR* gene in three randomly selected CLN1558, AVTO2225 and AVTO2226 plants each. a,b,c = indicate significance at $p < 0.05$. **g**. PCR results confirm that all AVTO2226 plants, but not CLN1558A plants, have a 200 bp PCR product associated with *Ty-6* resistance (the CAPS marker reported in 4) and that AVTO2225 plants are a mixed population carrying this CAPS marker. **h**. Sanger DNA sequencing and BLAST analyses show that the 500 bp PCR product and the 200 bp *Ty-6* CAPS marker PCR product in AVTO2226 plants and the resistant AVTO2225 plants have distinct DNA sequences (sequences 6.1 and 6.2, respectively), and that only the 200 bp *Ty-6* CAPS marker PCR product is associated with resistance to TYLCTHV infection (Table S1). The 500 bp PCR product is similar to a sequence on the *S. lycopersicum* chromosome 10 at position 62.32 Mb. **i**. PCR results confirm that CLN1558A does not carry the *Ty-1* and *Ty-2* resistance markers. Table S1 summarizes the *Ty* genotypes of the AVTO2225 and AVTO2226 plants used in this study. **j**. Images and symptom scores of TYLCTHV-infected leaves of CLN1558A plants and clonal plants from the original AVTO2225 and AVTO2226 lines. AVTO2225 and AVTO2226 genotypes that have the Ty-6 allele (Table S1) are resistant to TYLCTHV infection. For the statistical analysis of the symptom scores using the Mann-Whitney U test the score scale 0-4 established for tomato was transformed to a score scale 1-5. The Table summarizes the median symptom scores of systemic leaves and their means from the twelve plants of each tomato line. a, b, c = indicate that the median symptom scores are statistically significantly different between CLN1558 and AVTO2225 (U=144, p=6.24x10^{-6} for the two-sided test, p=3.12x10^{-6} for the one-sided test) and between AVTO2225 and AVTO2226 (U=120, p=0.00079 for the two-sided test, p=0.0004 for the one-sided test).

Table S1 Summary of *Ty* resistance alleles present in AVTO2225 and AVTO2226 lines based on PCR results using the primers for the *Ty-1* and *Ty-6* CAPS and *Ty-2* SCAR markers (see Table S4), and resistance of the genotypes to TYLCTHV infection (# = not determined).

AVTO2225	*Ty* genotype	Resistance to TYLCTHV	AVTO2226	*Ty* genotype	Resistance to TYLCTHV
1	*Ty-1 Ty-2*	No	1	*Ty-2 Ty-6*	Yes
2	*Ty-1 Ty-2*	No	2	*Ty-1 Ty-2 Ty-6*	Yes
3	*Ty-2*	No	3	*Ty-1 Ty-2 Ty-6*	Yes
4	*Ty-2 Ty-6*	Yes	4	*Ty-2 Ty-6*	Yes
5	*Ty-1 Ty-2*	No	5	*Ty-1 Ty-2 Ty-6*	Yes
6	*Ty-2*	No	6	*Ty-1 Ty-2 Ty-6*	Yes
7	*Ty-2 Ty-6*	Yes	7	*Ty-1 Ty-2 TY-6*	Yes
8	*Ty-2*	No	8	*Ty-2 Ty-6*	Yes
9	*Ty-1 Ty-2 Ty-6*	Yes	9	*Ty-2 Ty-6*	Yes
10	*Ty-1 Ty-2*	No	10	*Ty-1 Ty-2 Ty-6*	Yes
11	*Ty-2 Ty-6*	Yes	11	*Ty-1 Ty-2 Ty-6*	Yes
12	*Ty-2*	No	12	*Ty-2 Ty-6*	Yes
13	*Ty-2*	#	13	*Ty-2 Ty-6*	Yes
14	*Ty-1 Ty-2*	#	14	*Ty-2 Ty-6*	Yes
15	*Ty-1 Ty-2 Ty-6*	#	15	*Ty-2 Ty-6*	Yes
16	*Ty-1 Ty-2*	#	16	*Ty-2 Ty-6*	Yes
17	*Ty-1 Ty-2*	#	17	*Ty-2 Ty-6*	Yes
18	*Ty-2*	#	18	*Ty-2 Ty-6*	Yes
19	*Ty-2 Ty-6*	#	19	*Ty-2 Ty-6*	Yes
20	*Ty-2*	#	20	*Ty-2 Ty-6*	Yes

.

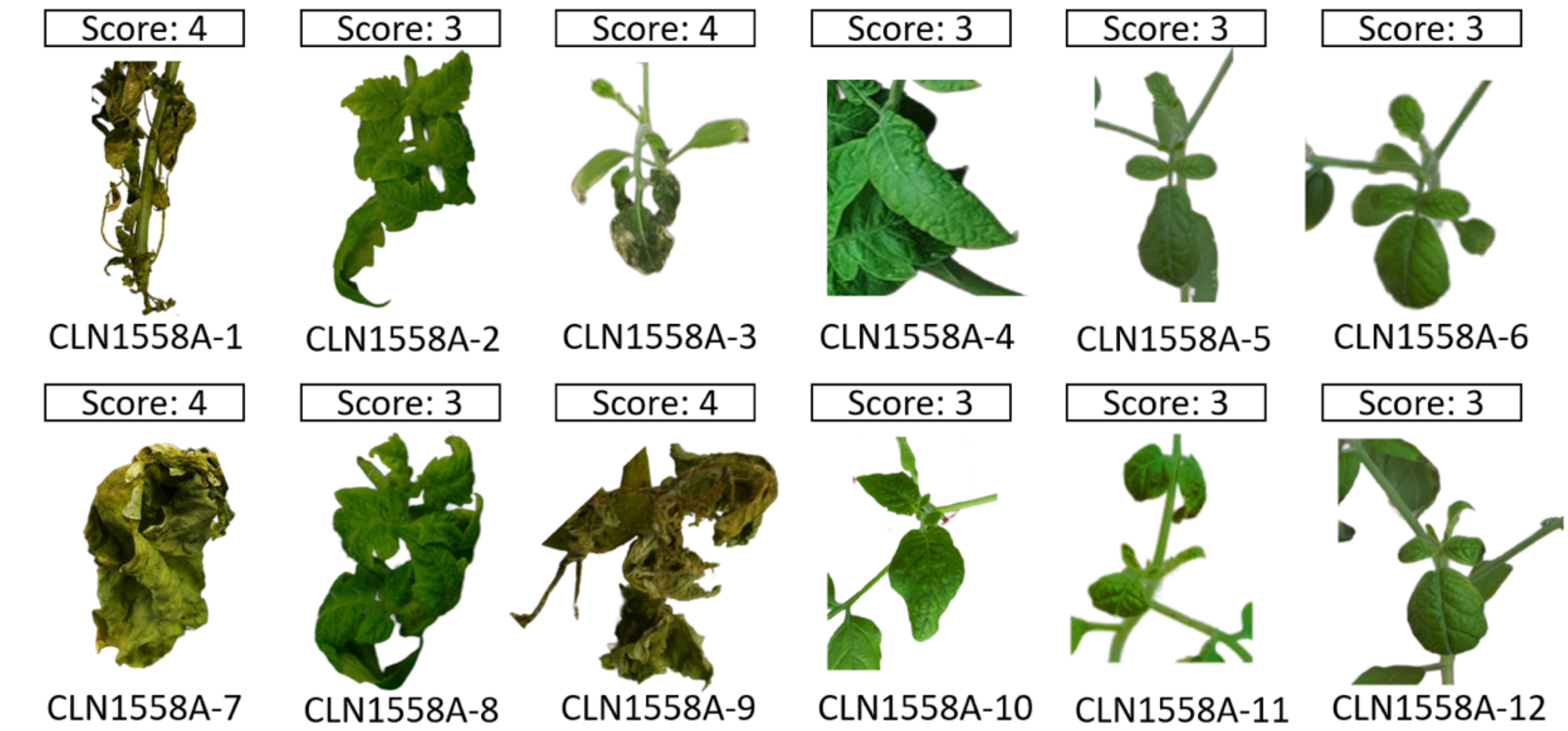
Score: 4
Score: 3
Score: 4
Score: 3
Score: 3
Score: 3
CLN1558A-1
CLN1558A-2
CLN1558A-3
CLN1558A-4
CLN1558A-5
CLN1558A-6
Score: 4
Score: 3
Score: 4
Score: 3
Score: 3
Score: 3
CLN1558A-7
CLN1558A-8
CLN1558A-9
CLN1558A-10
CLN1558A-11
CLN1558A-12

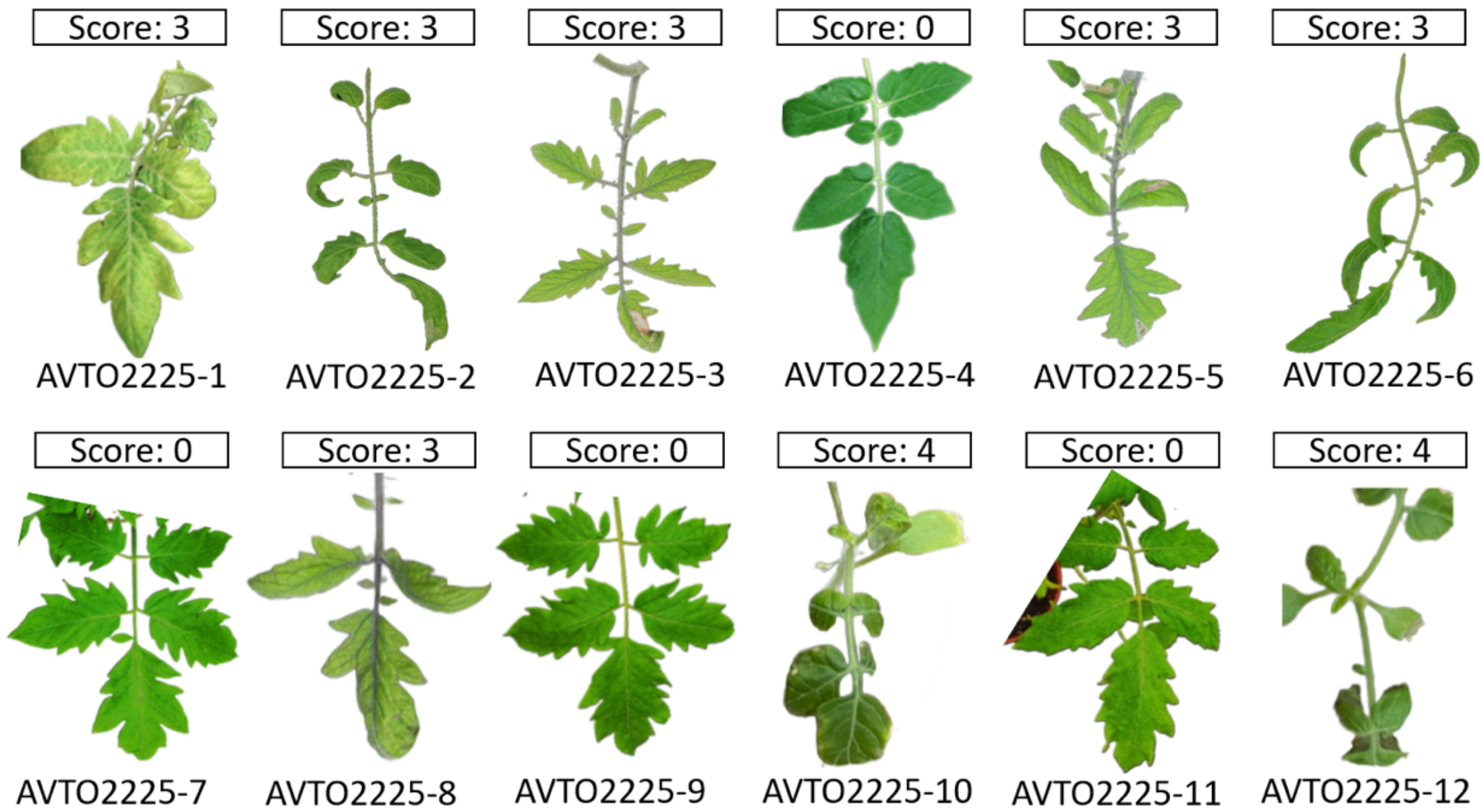
Score: 3
Score: 3
Score: 3
Score: 0
Score: 3
Score: 3
AVTO2225-1
AVTO2225-2
AVTO2225-3
AVTO2225-4
AVTO2225-5
AVTO2225-6
Score: 0
Score: 3
Score: 0
Score: 4
Score: 0
Score: 4
AVTO2225-7
AVTO2225-8
AVTO2225-9
AVTO2225-10
AVTO2225-11
AVTO2225-12

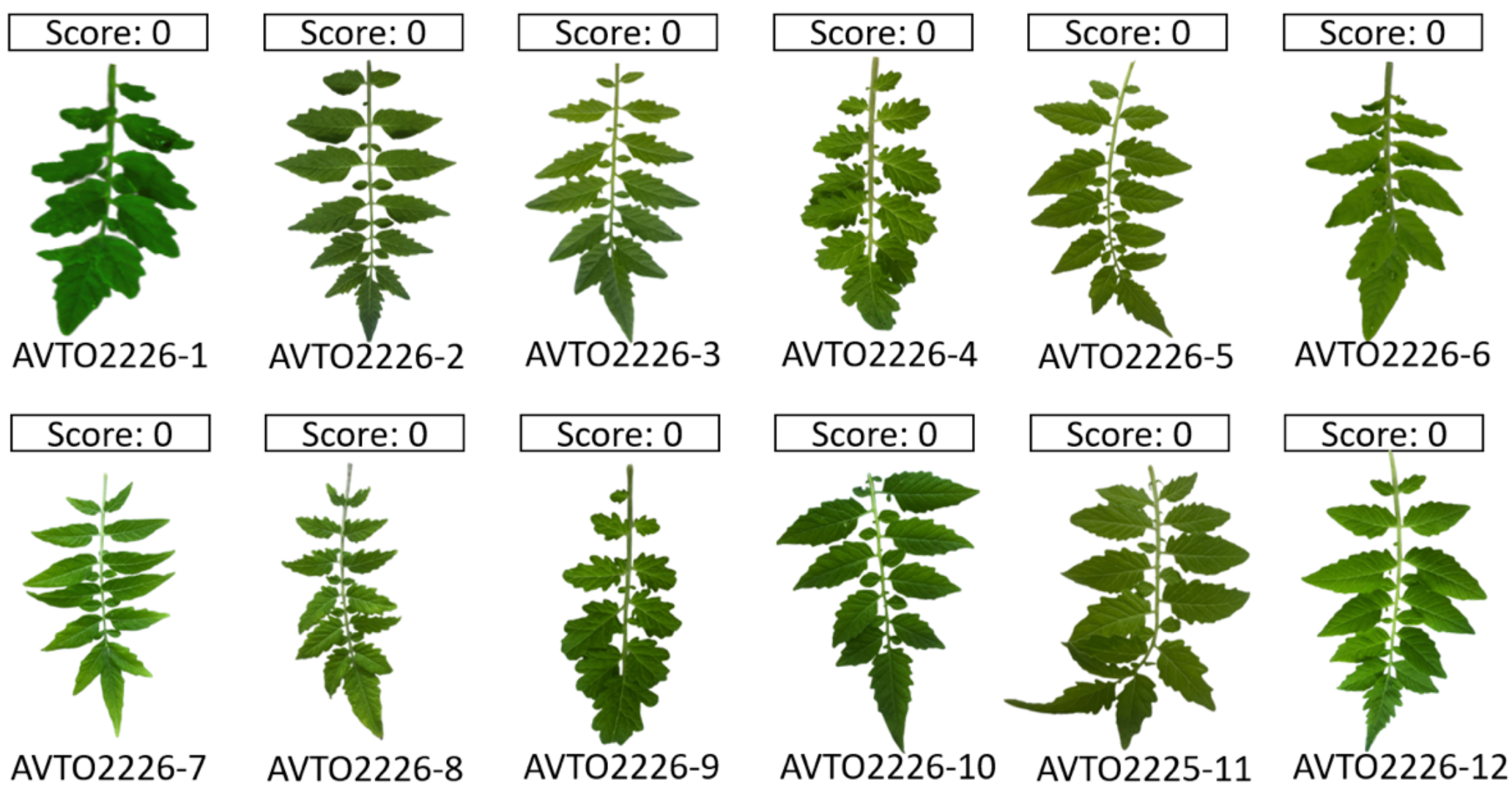
Score: 0
Score: 0
Score: 0
Score: 0
Score: 0
Score: 0
AVTO2226-1
AVTO2226-2
AVTO2226-3
AVTO2226-4
AVTO2226-5
AVTO2226-6
Score: 0
Score: 0
Score: 0
Score: 0
Score: 0
Score: 0
AVTO2226-7
AVTO2226-8
AVTO2226-9
AVTO2226-10
AVTO2225-11
AVTO2226-12

Tomato lines	Median symptom score*	Mean
CLN1558A[#]	5.0[a]	4.69
AVTO2225[#]	4.0[b]	3.19
AVTO2226[#]	1.0[c]	1.00

[#] n = 3, three replicates of the 12 CLN1558A plants and three replicate plants each of the 12 AVTO2225 and AVTO2226 genotypes (see Table S1) were infected with TYLCTHV. * The score scale 0-4 was transformed to 1-5 for statstical testing using the two-sided Mann-Whitney U statistical test. a, b, c = indicate that the median symptom scores are statistically significantly different between CLN1558 and AVTO2225 (U=1014.5, $p=7.26\times10^{-6}$ for the two-sided test, $p=3.63\times10^{-6}$ for the one-sided test) and between AVTO2225 and AVTO2226 (U=1008, $p=7.16\times10^{-6}$ for the two-sided test, $p=3.58\times10^{-6}$ for the one-sided test).

Figure S2

Examples of infection symptoms on leaves and symptom scores after TYLCTHV inoculation of susceptible and resistant genotypes of CLN1558A, AVTO2225 and AVTO2226 genotypes at 30 dpi (see Table S1) of a second independent infection experiment. All CLN1558A plants, which have no *Ty* resistance alleles, show leaf curling and wilting symptoms on systemic leaves. Most of the AVTO2225 plants show leaf curling symptoms on systemic leaves except AVTO2225-4, 7, 9 and 11 genotypes, which have the *Ty-6* resistance allele. The *Ty-1* and/or *Ty-2* resistance alleles are not sufficient to provide resistance to the severe TYLCTHV strain. All AVTO2226 plants have the *Ty-6* resistance allele (in addition to the *Ty-1* and/or *Ty-2* resistance alleles) and do not show leaf symptoms. The Table summarizes the median symptom scores and their mean of systemic leaves from the 36 plants (three replicates) of each tomato line. The significance of the differences between the CLN1558A, AVTO2225 and AVTO2226 median symptom scores after TYLCTHV infection was determined using the two-sided Mann-Whitney U statistical test.

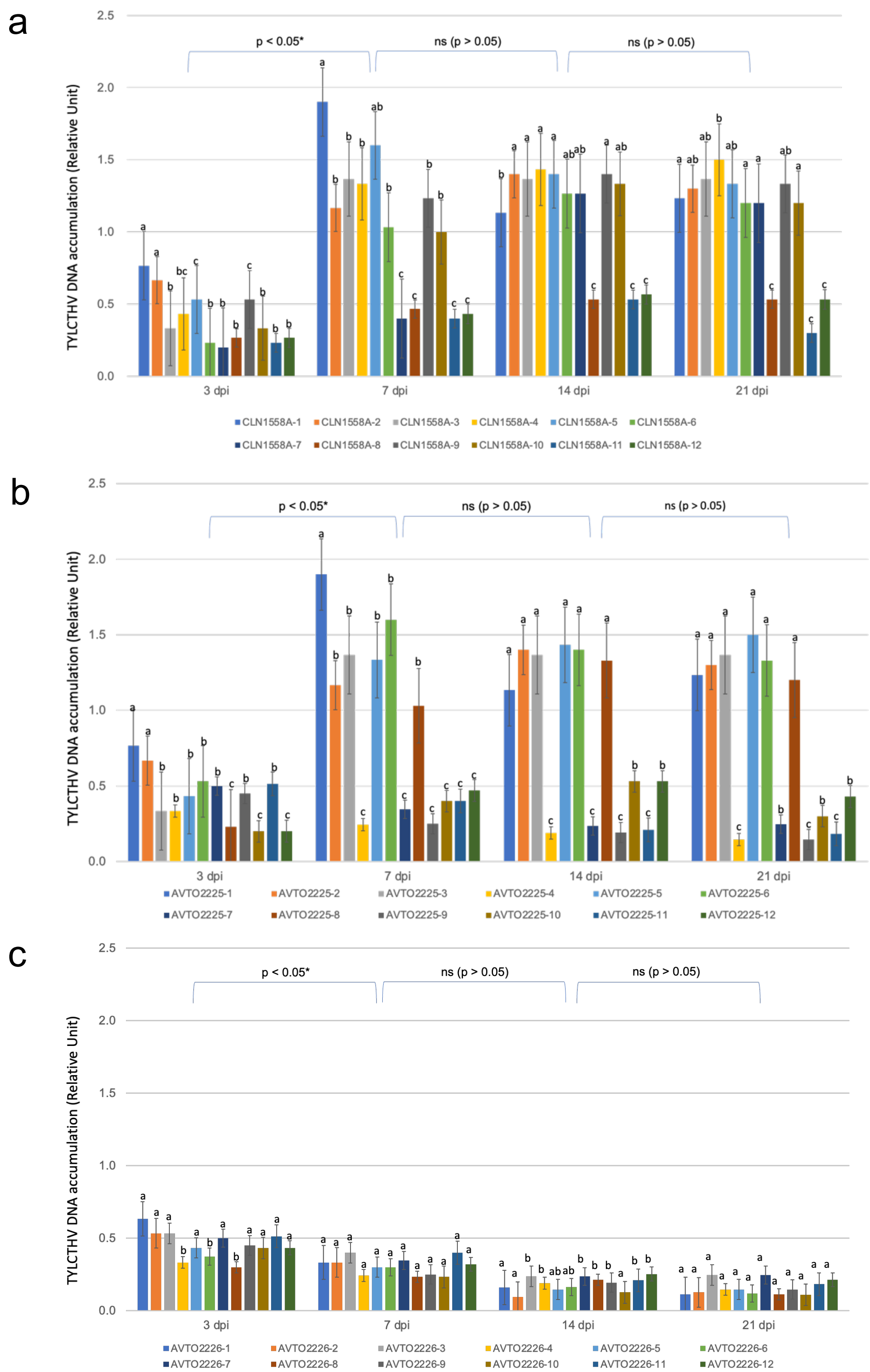

Figure S3

TYLCTHV cannot continue to replicate in inoculated AVTO2225 and AVTO2226 leaves with the *Ty-6* allele that confers resistance (see Table S1). **a**. TYLCTHV DNA accumulates quickly in the inoculated leaves seven days post inoculation (dpi) and

remains at high levels at 14 and 21 dpi in most of the CLN1558A plants. TYLCTHV DNA accumulation in the group of CLN1558A plants is signficantly higher between 3 and 7 dpi ($p < 0.05$*) but not significantly different between 7, 14, and 21 dpi (ns, $p > 0.05$), consistent with systemic movement of TYLCTHV in the susceptible tomato cultivar. **b**. TYLCTHV DNA accumulates quickly in inoculated leaves at 7 dpi and remains at high levels at 14 and 21 dpi in most of the AVTO2225 plants except for AVTO2225 plants 4, 7, 9 and 11 with the *Ty-6* resistance allele. TYLCTHV DNA accumulation in the group of AVTO2225 plants is signficantly higher between 3 and 7 dpi ($p < 0.05$*) but not significantly different between 7, 14, and 21 dpi (ns, $p > 0.05$). **c**. TYLCTHV DNA accumulates in inoculated leaves of AVTO2226 plants at 3 dpi to similar levels as in CLN1558A and AVTO2225 plants but then DNA levels decrease at 7 dpi and remain low at 14 and 21 dpi. The TYLCTHV DNA levels in the group of AVTO2226 plants that all have the *Ty-6* allele are signficantly lower at 7 dpi than 3 dpi ($p < 0.05$*) but not significantly different between 7, 14, and 21 dpi (ns, $p > 0.05$). a,b,c = indicate significance at $p < 0.05$; similar letters indicate no significant differences. Note that at 3 dpi signals for the Hyg^R gene in the T-DNA carrying the infectious clone of the TYLCTHV A and B DNA genomes can be detected in the leaf samples, indicating that the TYLCTHV DNA accumulation quantified using qPCR is likely a combination of DNA amplification from both the infectious clone and the replicating TYLCTHV DNA at this time point. Signals for the Hyg^R gene of the T-DNA infectious clone were not detected in leaf samples at 7 dpi (see Fig. S8).

a

```
Heinz1706      MNSGNSKKRSAAATPSPQQQQKHHAAMEEEDLDEDVFLEETLLQYEEDSQVLRELEESNS  60
CLN1558A       MNSGNSKKRSAAATPSPQQQQKHHAAMEEEDLDEDVFLEETLLQYEEDSQVLRELEESNS
AVT02225-S     MNSGNSKKRSAAATPSPQQQQKHHAAMEEEDLDEDVFLEETLLQYEEDSQVLRELEESNS
AVT02225-R     MNSGNSKKRSAAATPSPQQQQKHHAAMEEEDLDEDVFLEETLLQYEEDSQVLRELEESNS
AVT02226       MNSGNSKKRSAAATPSPQQQQKHHAAMEEEDLDEDVFLEETLLQYEEDSQVLRELEESNS
               ************************************************************

Heinz1706      IKERLQKWKRPSLSQAYIAQTQSISFQQLEIDYIIAESHKELLPNSSGQAAILRIFGVTK  120
CLN1558A       IKERLQKWKRPSLSQAYIAQTQSISFQQLEIDYIIAESHKELLPNSSGQAAILRIFGVTK
AVT02225-S     IKERLQKWKRPSLSQAYIAQTQSISFQQLEIDYIIAESHKELLPNSSGQAAILRIFGVTK
AVT02225-S     IKERLQKWKRPSLSQAYIAQTQSISFQQLEIDYIIAESHKELLPNSSGQAAILRIFGVTK
AVT02226       IKERLQKWKRPSLSQAYIAQTQSISFQQLEIDYIIAESHKELLPNSSGQAAILRIFGVTK
               ************************************************************

Heinz1706      EGNSVCCHVHGFEPYFYISCPPGMGPDDISRFHQILEGRMREANRNSNAPKFVRRIEVVQ  180
CLN1558A       EGNSVCCHVHGFEPYFYISCPPGMGPDDISRFHQILEGRMREANRNSNAPKFVRRIEVVQ
AVT02225-S     EGNSVCCHVHGFEPYFYISCPPGMGPDDISRFHQILEGRMREANRNSNAPKFVRRIEVVQ
AVT02225-R     EGNSVCCHVHGFEPYFYISCPPGMGPDDISRFHQILEGRMREANRNSNAPKFVRRIEVVQ
AVT02226       EGNSVCCHVHGFEPYFYISCPPGMGPDDISRFHQILEGRMREANRNSNAPKFVRRIEVVQ
               ************************************************************

Heinz1706      KRSIMYYQQQASQPFLKIVVALPTMVTSCRGILDRGIQIDGLGMKSFLTYESNVLFALRF  240
CLN1558A       KRSIMYYQQQASQPFLKIVVALPTMVTSCRGILDRGIQIDGLGMKSFLTYESNVLFALRF
AVT02225-S     KRSIMYYQQQASQPFLKIVVALPTMVTSCRGILDRGIQIDGLGMKSFLTYESNVLFALRF
AVT02225-R     KRSIMYYQQQASQPFLKIVVALPTMVTSCRGILDRGIQIDGLGMKSFLTYESNVLFALRF
AVT02226       KRSIMYYQQQASQPFLKIVVALPTMVTSCRGILDRGIQIDGLGMKSFLTYESNVLFALRF
               ************************************************************

Heinz1706      MIDCNIVGGNWIEVPVGKYKKAARNLSYCQLEFDCLYSDLISHSPEGEFSKMAPFRILSF  300
CLN1558A       MIDCNIVGGNWIEVPVGKYKKAARNLSYCQLEFDCLYSDLISHSPEGEFSKMAPFRILSF
AVT02225-S     MIDCNIVGGNWIEVPVGKYKKAARNLSYCQLEFDCLYSDLISHSPEGEFSKMAPFRILSF
AVT02225-R     MIDCNIVGGNWIEVPVGKYKKAARNLSYCQLEFDCLYSDLISHSPEGEFSKMAPFRILSF
AVT02226       MIDCNIVGGNWIEVPVGKYKKAARNLSYCQLEFDCLYSDLISHSPEGEFSKMAPFRILSF
               ************************************************************

Heinz1706      DIECAGRKGHFPEPTQDPVIQVANLVTLQGQDQPLIRNVMTLKSCSPIVGVDIMSFDTEK  360
CLN1558A       DIECAGRKGHFPEPTQDPVIQVANLVTLQGQDQPLIRNVMTLKSCSPIVGVDIMSFDTEK
AVT02225-S     DIECAGRKGHFPEPTQDPVIQVANLVTLQGQDQPLIRNVMTLKSCSPIVGVDIMSFDTEK
AVT02225-R     DIECAGRKGHFPEPTQDPVIQVANLVTLQGQDQPLIRNVMTLKSCSPIVGVDIMSFDTEK
AVT02226       DIECAGRKGHFPEPTQDPVIQVANLVTLQGQDQPLIRNVMTLKSCSPIVGVDIMSFDTEK
               ************************************************************

Heinz1706      EVLRAWRDLIREVDPDIIIGYNICNFDLPYLIRRAEVLGMAEFPVLGRIRNSRVRVKDAT  420
CLN1558A       EVLRAWRDLIREVDPDIIIGYNICNFDLPYLIRRAEVLGMAEFPVLGRIRNSRVRVKDAT
AVT02225-S     EVLRAWRDLIREVDPDIIIGYNICNFDLPYLIRRAEVLGMAEFPVLGRIRNSRVRVKDAT
AVT02225-R     EVLRAWRDLIREVDPDIIIGYNICNFDLPYLIRRAEVLGMAEFPVLGRIRNSRVRVKDAT
AVT02226       EVLRAWRDLIREVDPDIIIGYNICNFDLPYLIRRAEVLGMAEFPVLGRIRNSRVRVKDAT
               ************************************************************

Heinz1706      FSSRQHGTRESKEITIEGRVQFDLFQAVQRDYKLSSYSLNSVSAHFLNEQKEDVHHSIIS  480
CLN1558A       FSSRQHGTRESKEITIEGRVQFDLFQAVQRDYKLSSYSLNSVSAHFLNEQKEDVHHSIIS
AVT02225-S     FSSRQHGTRESKEITIEGRVQFDLFQAVQRDYKLSSYSLNSVSAHFLNEQKEDVHHSIIS
AVT02225-R     FSSRQHGTRESKEITIEGRVQFDLFQAVQRDYKLSSYSLNSVSAHFLNEQKEDVHHSIIS
AVT02226       FSSRQHGTRESKEITIEGRVQFDLFQAVQRDYKLSSYSLNSVSAHFLNEQKEDVHDSIIS
               *******************************************************.****

Heinz1706      DLQNGNAETRRRLAVYCLKDAYLPQRLLDKLMFIYNYVEMARVTGVPISFLLSRGQSIKV  540
CLN1558A       DLQNGNAETRRRLAVYCLKDAYLPQRLLDKLMFIYNYVEMARVTGVPISFLLSRGQSIKV
AVT02225-S     DLQNGNAETRRRLAVYCLKDAYLPQRLLDKLMFIYNYVEMARVTGVPISFLLSRGQSIKV
AVT02225-R     DLQNGNAETRRRLAVYCLKDAYLPQRLLDKLMFIYNYVEMARVTGVPISFLLSRGQSIKV
AVT02226       DLQNGNAETRRRLAVYCLKDAYLPQRLLDKLMFIYNYVEMARVTGVPISFLLSRGQSIKV
               ************************************************************

Heinz1706      LSQLLRKAKQKDLVIPNAKQVGSEQGTFEGATVLEAQAGFYEKPIATLDFASLYPSIMMA  600
CLN1558A       LSQLLRKAKQKDLVIPNAKQVGSEQGTFEGATVLEAQAGFYEKPIATLDFASL-PSIMMA
AVT02225-S     LSQLLRKAKQKDLVIPNAKQAGSEQGTFEGATVLEARAGFYEKPIATLDFASL-PSIMMA
AVT02225-R     LSQLLRKAKQKDLVIPNAKQAGSEQGTFEGATVLEARAGFYEKPIATLDFASLYPSIMMA
AVT02226       LSQLLRKAKQKDLVIPNAKQVGSEQGTFEGATVLEAQAGFYEKPIATLDFASLYPSIMMA
               ********************.***************:**************** ******

Heinz1706      YNLCYCTLVTPEEVRKLNLPPECVNKTPSGETFVKSTLQKGILPEILEELLTARKRAKAD  660
CLN1558A       YNLCYCTLVTPEEVRKLNLPPECVNKTPSGETFVKSTLQKGILPEILEELLTARKRAKAD
AVT02225-S     YNLCYCTLVTPEEVRKLNLPPECVNKTPSGETFVKSTLQKGILPEILEELLTARKRAKAD
AVT02225-R     YNLCYCTLVTPEEVRKLNLPPDCVNKTPSGETFVKSTLQKGILPEILEELLTARKRAKAD
AVT02226       YNLCYCTLVTPEEVRKLNLPPDCVNKTPSGETFVKSTLQKGILPEILEELLTARKRAKAD
               *********************:**************************************
```

```
Heinz1706                LKEATDPRVKAVLDGRQLALKISANSVYGFTGATVGQLPCLEISSSVTSYGRQMIEHTKK  720
CLN1558A                 LK-ATDPRVKAVLDGRQLALKISANSVYGFTGATVGQLPCLEISSSVTSYGRQMIEHTKK
AVTO2225-S               LKEATDPRVKAVLDGRQLALKISANSVYGFTGATVGQLPCLEISSSVTSYGRQMIEHTKK
AVTO2225-R               LKEATDPRVKAVLDGRQLALKISANSVYGFTGATVGQLPCLEISSSVTSYGRQMIEHTKK
AVTO2226                 LKEATDPRVKAVLDGRQLALKISANSVYGFTGATVGQLPCLEISSSVTSYGRQMIEHTKK
                         ** *********************************************************

Heinz1706                LVEDKFTVLGGYEHNAEVIYGDTDSVMVQFGVPTVEEAMKLGREAADYISGTFIKPIKLE  780
CLN1558A                 LVEDKFTVLGGYEHNAEVIYGDTDSVMVQFGVPTVEEAMKLGREAADYISGTFIKPIKLE
AVTO2225-S               LVEDKFTVLGGYEHNAEVIYGDTDSVMVQFGVPTVEEAMKLGREAADYISGTFIKPIKLE
AVTO2225-R               LVEDKFTVLGGYEHNAEVIYGDTDSVMVQFGVPTVEEAMKLGREAADYISGTFIKPIKLE
AVTO2226                 LVEDKFTVLGGYEHNAEVIYGDTDSVMVQHGVPTVEEAMKLGREAADYISGTFIKPIKLE
                         *****************************.******************************

Heinz1706                FEKIYYPYLLISKKRYAGLLWTNPDKHDKMDAKGIETVRRDNCLLVKNLVTECLYKILVD  840
CLN1558A                 FEKIYYPYLLISKKRYAGLLWTNPDKHDKMDAKGIETVRRDNCLLVKNLVTECLYKILVD
AVTO2225-S               FEKIYYPYLLISKKRYAGLLWTNPDKHDKMDAKGIETVRRDNCLLVKNLVTECLYKILVD
AVTO2225-R               FEKIYYPYLLISKKRYAGLLWTNPDKHDKMDAKGIETVRRDNCLLVKNLVTECLYKILVD
AVTO2226                 FEKIYYPYLLISKERYAGLLWTNPDKHDKMDAKGIETVRRDNCLLVKNLVTECLYKILVD
                         *************:**********************************************

Heinz1706                RDVPGAVQYVKNTISDLLLNRMDLSLLVITHGLTKTGDDYEVKAAHVELAERMRKRDAAT  900
CLN1558A                 RDVPGAVQYVKNTISDLLLNRMDLSLLVITHGLTKTGDDYEVKAAHVELAERMRKRDAAT
AVTO2225-S               RDVPGAVQYVKNTISDLLLNRMDLSLLVITKGLTKTGDDYEVKTAHVELAERMRKRDAAT
AVTO2225-R               RDVPGAVQYVKNTISDLLLNRMDLSLLVITKGLTKTGDDYEVKTAHVELAERMRKRDAAT
AVTO2226                 RDVPGAVQYVKNTISDLLLNRMDLSLLVITKGLTKTGDDYEVKAAHVELAERMRKRDAAT
                         ******************************:************:****************

Heinz1706                APSIGDRVPYVIIKAAKGAKAYEKSEDPIYVLENNIPIDPQYYLENQISKPLLRIFEPIL  960
CLN1558A                 APSIGDRVPYVIIKAAKGAKAYEKSEDPIYVLENNIPIDPQYYLENQISKPLLRIFEPIL
AVTO2225-S               APSIGDRVPYVIIKAAKGAKAYEKSEDPIYVLENNIPIDPQYYLENQISKPLLRIFEPIL
AVTO2225-R               APSIGDRVPYVIIKAAKGAKAYEKSEDPIYVLENNIPIDPQYYLENQISKPLLRIFEPIL
AVTO2226                 APSIGDRVPYVIIKAAKGAKAYEKSEDPIYVLENNIPIDPQYYLENQISKPLLRIFEPIL
                         ************************************************************

Heinz1706                KNASKELLHGDHTRSISVPTPSNSGIMRFAKKQLTCIGCKTPLSGSDRTICKHCKGREAE  1020
CLN1558A                 KNASKELLHGDHTRSISVPTPSNSGIMRFAKKQLTCIGCKTPLSGSDRTICKHCKGREAE
AVTO2225-S               KNASKELLHGDHTRSIAVPTPSNSGIMRFAKKQLTCIGCKTPLSGSDRTICKHCKGREAE
AVTO2225-R               KNASKELLHGDHTRSIAVPTPSNSGIMRFAKKQLTCIGCKTPLSGSDRTICKHCKGREAE
AVTO2226                 KNASKELLHGDHTRSISVPTPSNSGIMRFAKKQLTCIGCKTPLSGSDRTICKHCKGREAE
                         ****************:*******************************************

Heinz1706                LYCRSVFNVAELENLFGKLWTQCQECQGSLHQDVLCTSRDC-IFYRRKKAQKDMAEAKTQ  1080
CLN1558A                 LYCRSVANVAELENLFGKLWTQCQECQGSLHQDVLCTSRDCPIFYRRKKAQKDMAEAKTQ
AVTO2225-S               LYCRTVANVAELENLFGKLWTQCQECQGSLHQDVLCTSRDCPIFYRRKKAQKDMAEAKTQ
AVTO2225-R               LYCRTVANVAELENLFGKLWTQCQECQGSLHQDVLCTSRDCPIFYRRKKAQKDMAEAKTQ
AVTO2226                 LYCRSVANVAELENLFGKLWTQCQECQGSLHQDVLCTSRDCPIFYRRKKAQKDMAEAKTQ
                         ****:*:********************************** ******************

Heinz1706                LDRWNF                                                        1086
CLN1558A                 LDRWNF
AVTO2225-S               LDRWNF
AVTO2225-R               LDRWNF
AVTO2226                 LDRWNF
                         ******
```

b

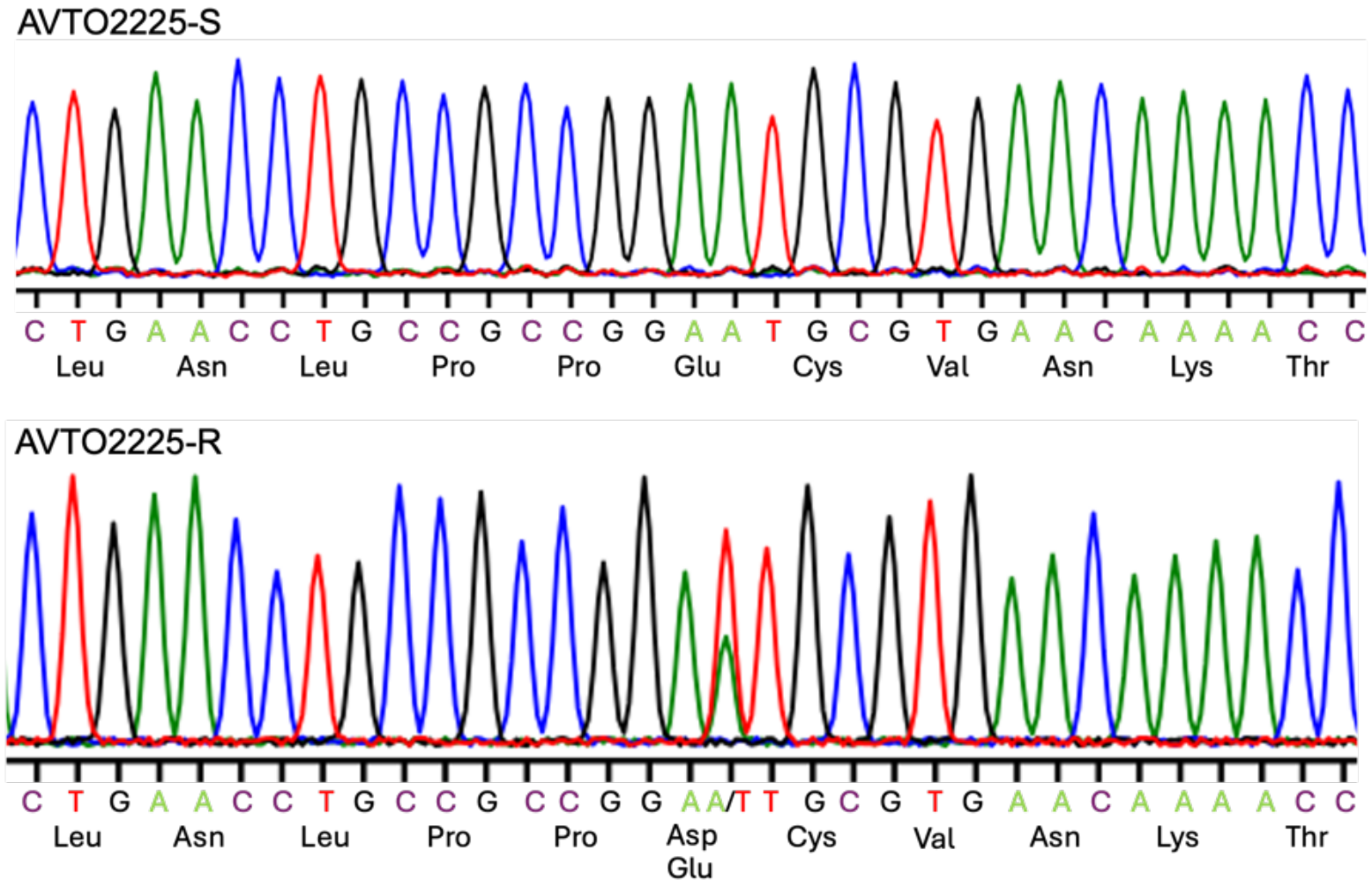

Figure S4

Amino acid sequence alignments confirm the presence of heterozygous alleles for POLD1 wild type and POLDE622D mutant proteins in AVTO2225-R and AVTO2226 plants. **a**. Amino acid sequence alignment of POLD1 proteins from the Heinz 1706 genotype (5, 6) and the CLN1558A, AVTO2225-S, AVTO2225-R and AVTO2226 plants. The red highlight at amino acid position 622 marks the E and D amino acid change in the AVTO2225-R and AVTO2226 POLD1 proteins encoded by the heterozygous *POLD1*^{E622D} alleles associated with resistance to TYLCTHV infections. All other POLD1 amino acid sequence polymorphisms between the CLN1558A, AVTO2225 and AVTO2226 genomes, highlighted in yellow, are homozygous. **b**. Sanger DNA sequence traces after base calling bracketing the heterozygous non-synonymus A/T nucleotide polymorphism in AVTO2225-R plants that have the *Ty-6 POLD*^{E622D} allele but that is not present in AVTO2225-S plants.

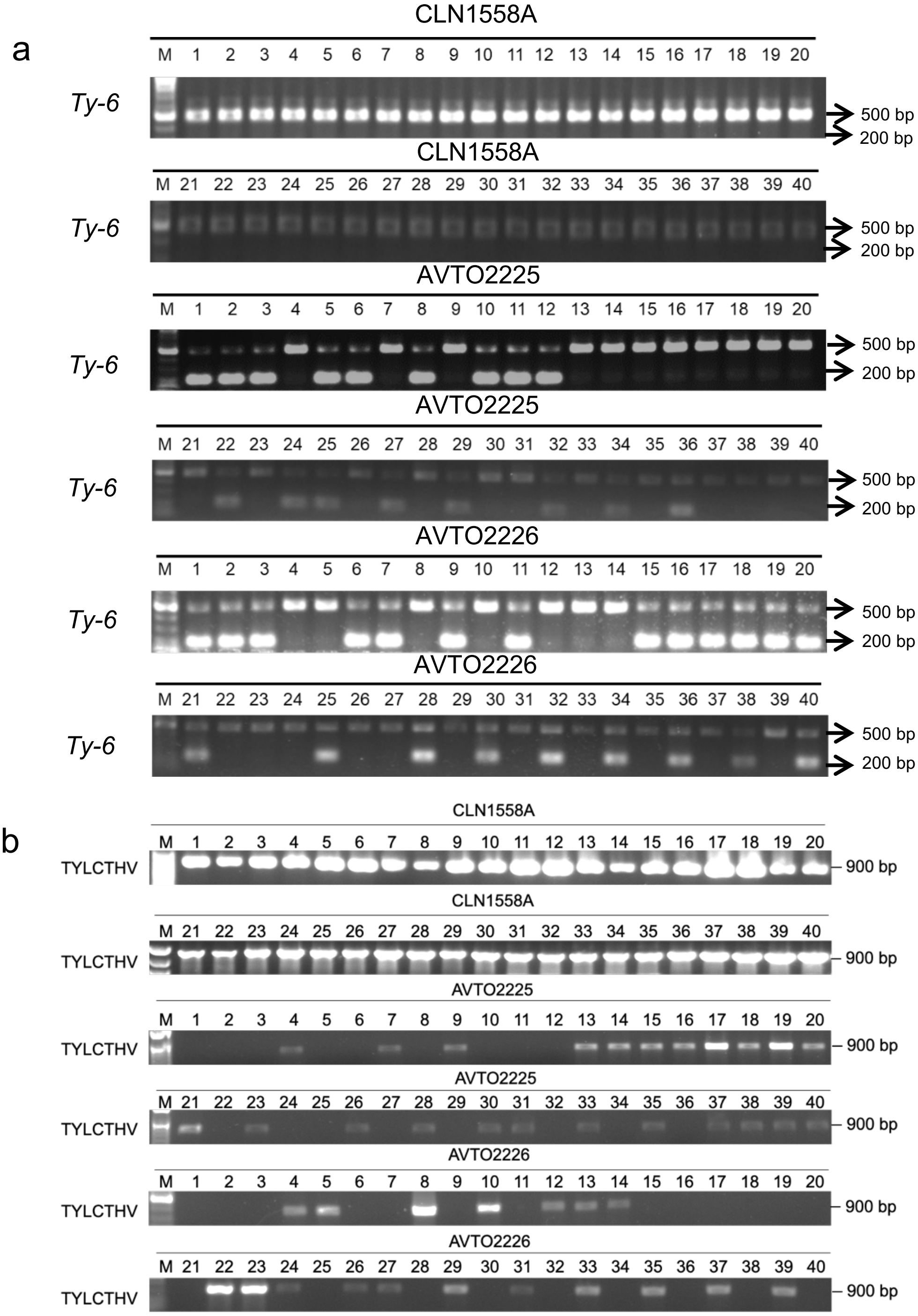

c

```
F1 AVTO2225-1  M A Y N L C Y C T L V T P P E V R K L N L P P D C V N K T P S G E T F V
F1 AVTO2225-2  M A Y N L C Y C T L V T P P E V R K L N L P P D C V N K T P S G E T F V
F1 AVTO2225-3  M A Y N L C Y C T L V T P P E V R K L N L P P D C V N K T P S G E T F V
F1 AVTO2225-4  M A Y N L C Y C T L V T P P E V R K L N L P P E C V N K T P S G E T F V
F1 AVTO2225-7  M A Y N L C Y C T L V T P P E V R K L N L P P E C V N K T P S G E T F V
F1 AVTO2225-9  M A Y N L C Y C T L V T P P E V R K L N L P P E C V N K T P S G E T F V

F1 AVTO2226-1  M A Y N L C Y C T L V T P P E V R K L N L P P D C V N K T P S G E T F V
F1 AVTO2226-2  M A Y N L C Y C T L V T P P E V R K L N L P P D C V N K T P S G E T F V
F1 AVTO2226-3  M A Y N L C Y C T L V T P P E V R K L N L P P D C V N K T P S G E T F V
F1 AVTO2226-4  M A Y N L C Y C T L V T P P E V R K L N L P P E C V N K T P S G E T F V
F1 AVTO2226-5  M A Y N L C Y C T L V T P P E V R K L N L P P E C V N K T P S G E T F V
F1 AVTO2226-8  M A Y N L C Y C T L V T P P E V R K L N L P P E C V N K T P S G E T F V
```

Figure S5

The *POLD*^{E622D} allele, which is associated with the *Ty-6* CAPS marker, is dominant and linked to resistance against TYLCTHV infection. **a**. The population of 40 CLN1558A F1 plants carries only the 500 bp PCR product obtained with the DNA primers for the *Ty-6* CAPS marker reported in (4), which is not linked to resistance against TYLCTHV infection. The population of 40 AVTO2225 F1 plants, which are the offspring of a self-fertilized AVTO2225-R-9 plant, segregates for the *Ty-6* CAPS marker PCR product that is linked to the *POLD*^{E622D} allele and resistance against TYLCTHV infection. The population of 40 AVTO2226 F1 plants obtained from an assisted self-cross of a AVTO2226-1 parent segregates for the *Ty-6* CAPS marker linked to the *POLD*^{E622D} allele and associated with TYLCTHV resistance. **b**. TYLCTHV DNA was detected in systemic leaves from all inoculated CLN1558A F1 plants. TYLCTHV DNA was also detected in systemic leaves of AVTO2225 and AVTO2226 F1 plants that lack the *Ty-6* CAPS marker, but not in AVTO2225 and AVTO2226 plants with the *Ty-6* CAPS marker linked to the *POLD*^{E622D} allele. **c**. AVTO2225 and AVTO2226 F1 plants selected for the *Ty-6* CAPS marker associated with TYLCTHV resistance (see panels in **a**.) have the heterozygous mutant allele encoding POLD1^{E622D} (highlighted in red), whereas plants lacking the *Ty-6* CAPS marker have only the wild-type gene encoding POLD1. No plants with only the *Ty-6* CAPS marker that is genetically linked to the *POLD1*^{E622D} allele were identified in the AVTO2225-R and AVTO2226 F1 populations, indicating that plants homozygous for the *POLD1*^{E622D} allele are not viable.

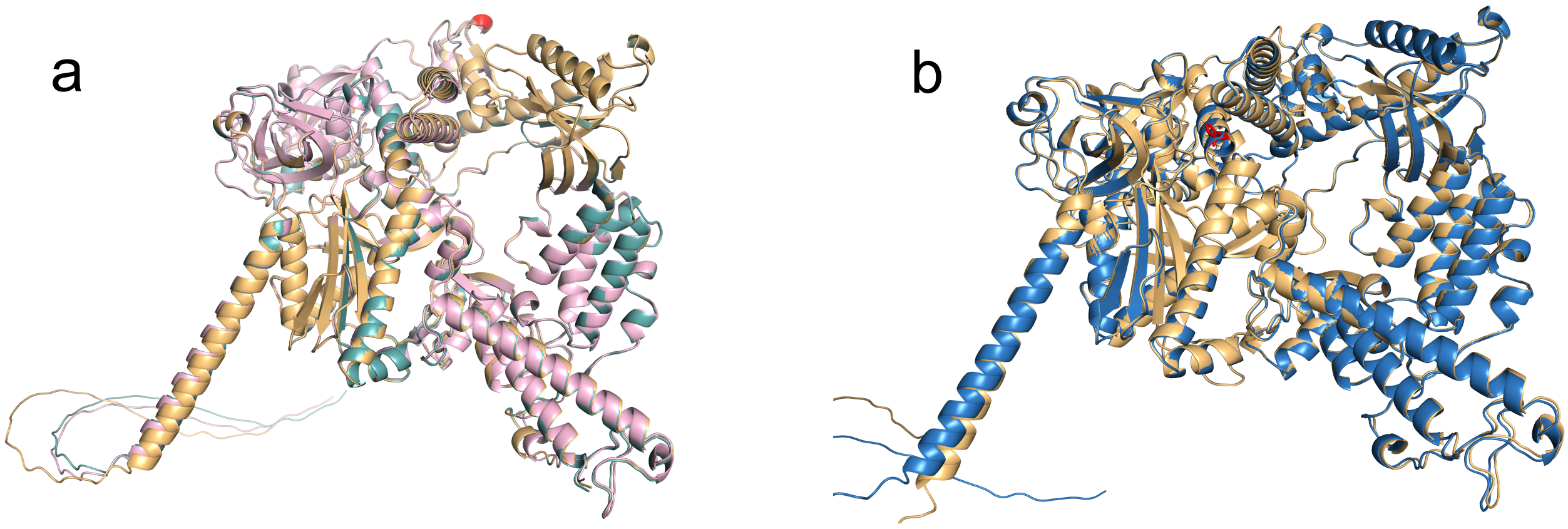

Figure S6

AlphaFold3 structural predictions of tomato POLD1. **a**. Superimposition of the predicted structural models of AVTO2225 POLD1 and POLD1^{E622D} proteins. The amino acid change in POLD1^{E622D} is shown in red. **b**. Super-imposition of the predicted structural models of Fla. 8696 POLD1 and POLD1^{Y515H} reported in (7). The amino acid change in POLD1^{Y515H} is shown in red.

Figure S7 can be found at the end of this file on page 29

Comparison of additional *AC1* and *AC2* DNA sequences obtained from TYLCTHV DNA isolated from AVTO2225-R and AVTO2226 TYLCTHV-inoculated leaves at 7 dpi. **a**. Comparison of *AC1* DNA sequences from TYLCTHV DNA isolated from AVTO2225-R-7, 9 and 11 plants. **b**. Comparison of *AC2* DNA sequences from TYLCTHV DNA isolated from AVTO2225-R-7, 9 and 11 plants. **c**. Comparison of *AC1* DNA sequences from TYLCTHV DNA isolated from AVTO2226 plants **d.** Comparison of *AC2* DNA sequences from TYLCTHV DNA isolated from AVTO2226 plants. * indicates no nucleotide changes *in AC1* and *AC2* DNA sequences from TYLCTHV DNA isolated from the plants.

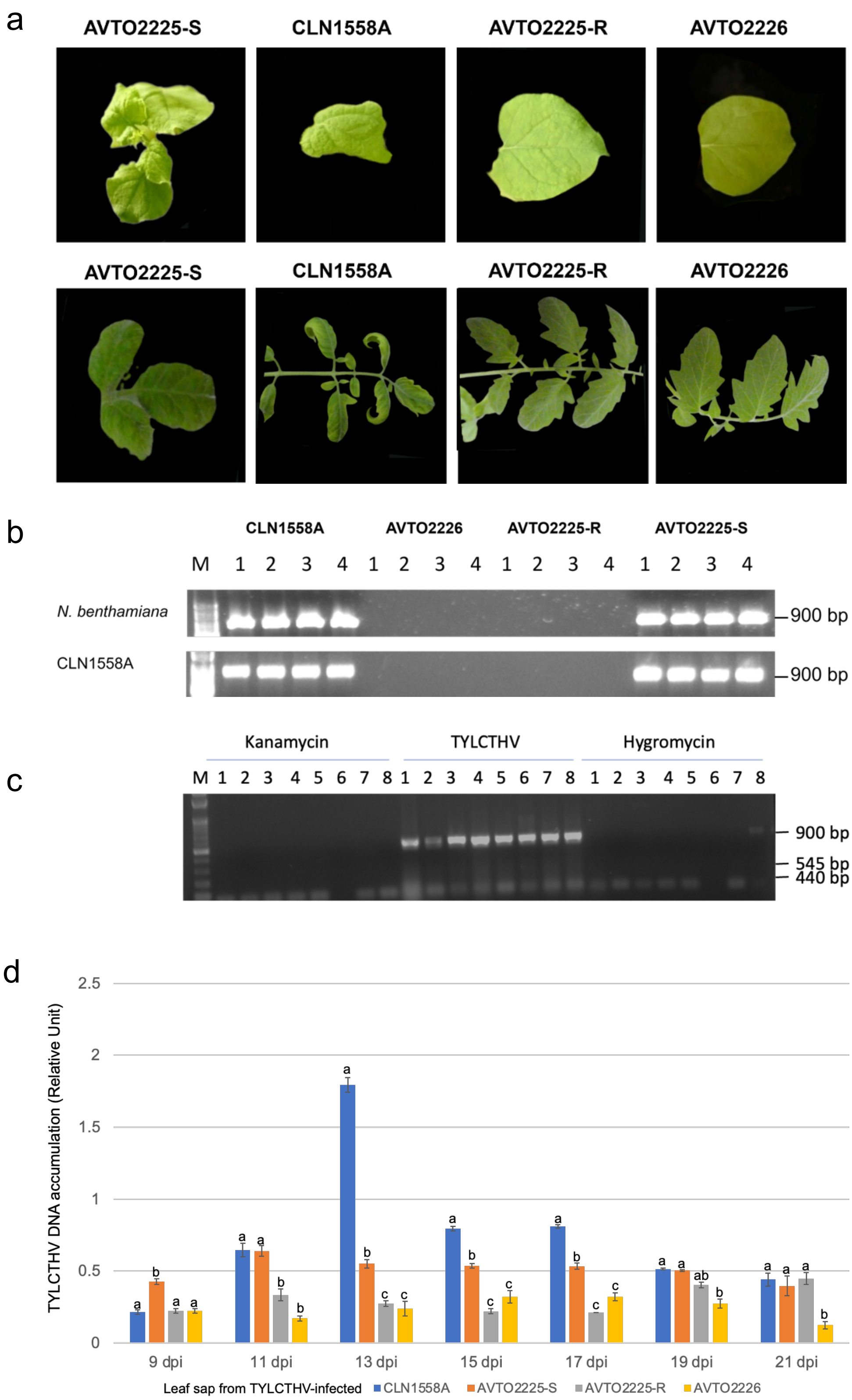

Figure S8

Mechanical inoculation of *N. benthamiana* and CLN1558A leaves with leaf sap from CLN1558A, AVTO2225-S, AVTO2225-R or AVTO2226 leaves infected with TYLCTHV. **a**. Examples of TYLCTHV symptoms on systemic *N. benthamiana* leaves at 15 dpi (top

panels) and systemic CLN1558A leaves at 30 dpi (bottom panels). Inoculation with leaf sap from TYLCTHV-infected AVTO2225-R or AVTO2226 leaves does not produce symptoms. **b**. PCR detection of TYLCTHV DNA in systemic *N. benthamiana* and systemic CLN1558A leaves after mechanical inoculation of four independent plants with leaf sap from CLN1558A, AVTO2226, AVTO2225-R or AVTO2225-S leaves infected with TYLCTHV. No TYLCTH DNA can be detected in systemic leaves of plants inoculated with leaf sap from TYLCTHV-infected AVTO2225-R or AVTO2226 leaves. **c**. Detection of PCR signals diagnostic for the presence of Agrobacterium T-DNA plasmid (Kan^R gene, 438 bp), T-DNA (Hyg^R gene, 545 bp) as well as TYLCTHV (900 bp) in leaf saps from leaves infected with TYLCTHV obtained at 7 dpi. Leaf saps were from the following plants (see Table S1): lanes 1 and 2, AVTO2225-S-1 and 2; lanes 3 and 4, CLN1558A-1 and 2; lanes 5 and 6, AVTO2225-R-4 and 7; lanes 7 and 8, AVTO2226-1 and 2. The leaf saps did not contain detectable levels of the Agrobacterium T-DNA plasmid or T-DNA carrying the infectious TYLCTHV clone. **d**. TYLCTHV DNA accumulation in CLN1558A leaf segments collected from the inoculated leaves at 9, 11, 13, 15, 17, 19 and 21 dpi after mechanical inoculation with leaf sap from TYLCTHV-infected CLN1558A, AVTO2225-S, AVTO2225-R or AVTO2226 leaves. a, b, c = indicate significant differences of TYLCTHV DNA amounts at each dpi between CLN1558A leaves inoculated with leaf sap from TYLCTHV-infected CLN1558A, AVTO2225-S, AVTO2225-R or AVTO2226 plants ($p < 0.05$); the same letter indicates no difference ($p > 0.05$). The higher TYLCTHV DNA accumulation is not significantly different between CLN1558A leaves inoculated with leaf sap from TYLCTHV-infected CLN1558A or AVTO2225-S plants ($p > 0.05$), but TYLCTHV DNA levels are significantly lower in CLN1558A leaves inoculated with leaf sap from AVTO2225-R ($p = 0.04$) or leaf sap from AVTO2226 ($p = 0.02$) compared to CLN1558A leaves inoculated with leaf sap from TYLCTHV-infected CLN1558A.

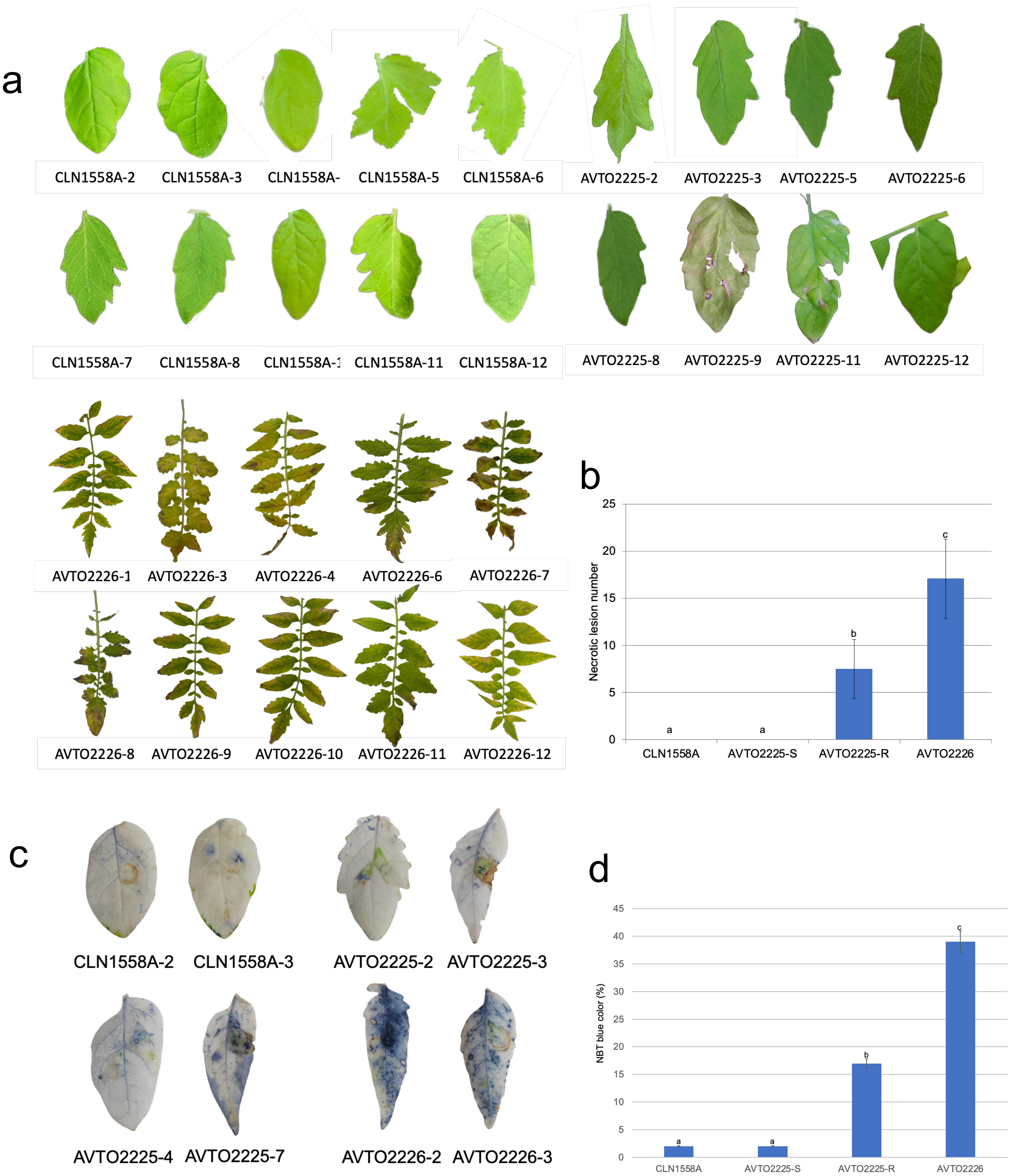

Figure S9

The presence of the heterozygous *Ty-6* *POLD1*^{E622D} allele is associated with a hypersensitive response (HR) in leaves after inoculation with TYLCTHV (see Table S1). **a**. CLN1558A does not have the *Ty-6* allele and leaves do not show necrotic lesions after TYLCTHV infection. Except AVTO2225-9 and 11, the other six AVTO2225 plants do not have the *Ty-6* allele and do not show necrotic lesions. All AVTO2226 plants have the Ty-6 allele and show necrotic lesions after TYLCTHV infection. **b**. The number of

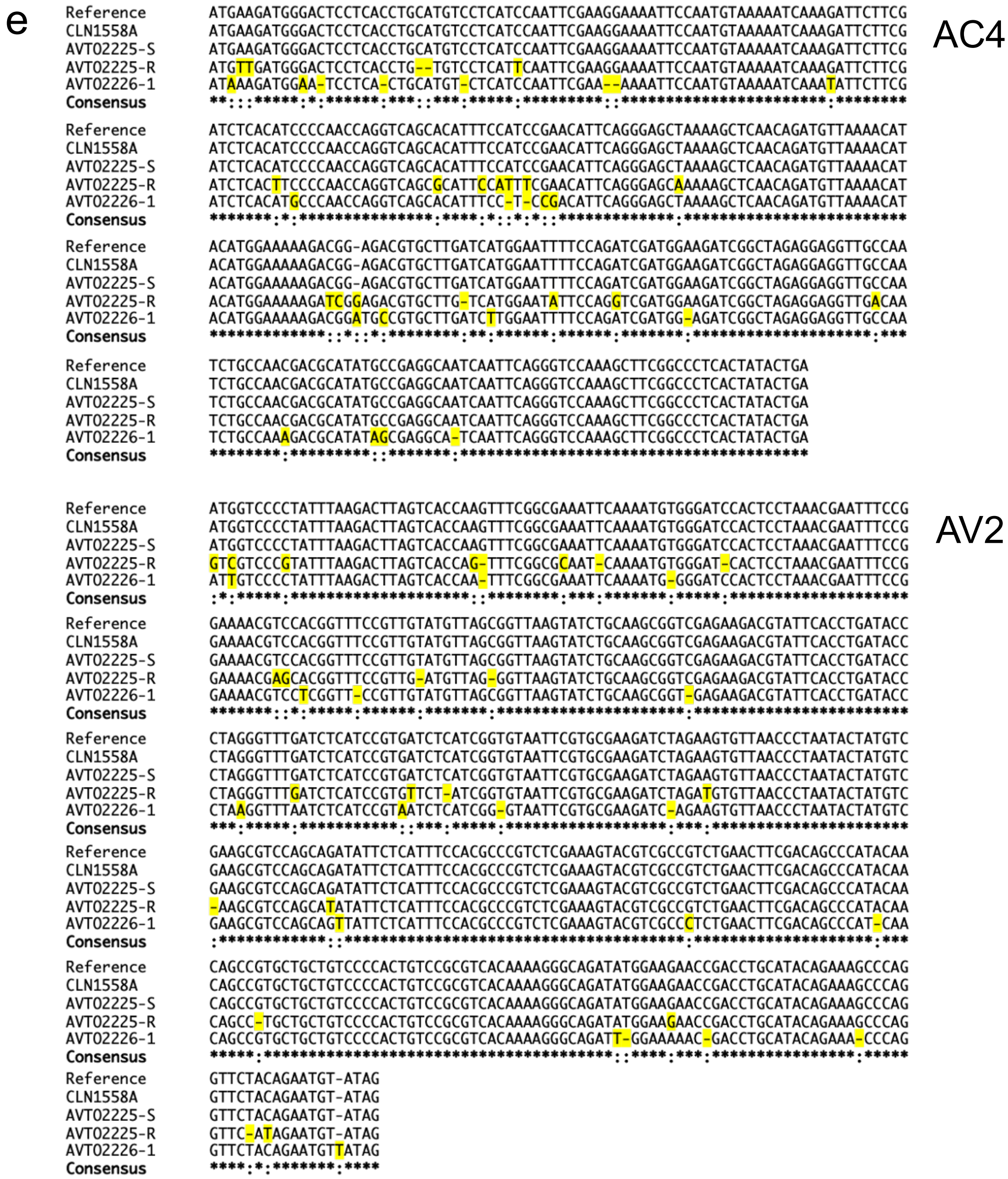

Figure S9, continued

necrotic lesions is significantly increased in AVTO2225-R and AVTO2226 genotypes that have the *Ty-6* allele ($p < 0.05$). **c**. Examples of leaves stained with NBT to confirm the presence of H_2O_2 ROS molecules produced during the HR. AVTO2225-R and AVTO2226 genotypes with the *Ty-6* allele display a stronger blue staining. **d**. H_2O_2 accumulation is significantly higher in AVTO2225-R and AVTO2226 genotypes ($p < 0.05$). **e**. The *AC4* and *AV2* genes that encode TYLCTHV HR suppressors (13,14) accumulate mutations in AVTO2225-R and AVTO2226 genotypes with the *Ty-6* *POLD1*^{E622D} allele, indicating that the AC4 and AV2 proteins are not functional.

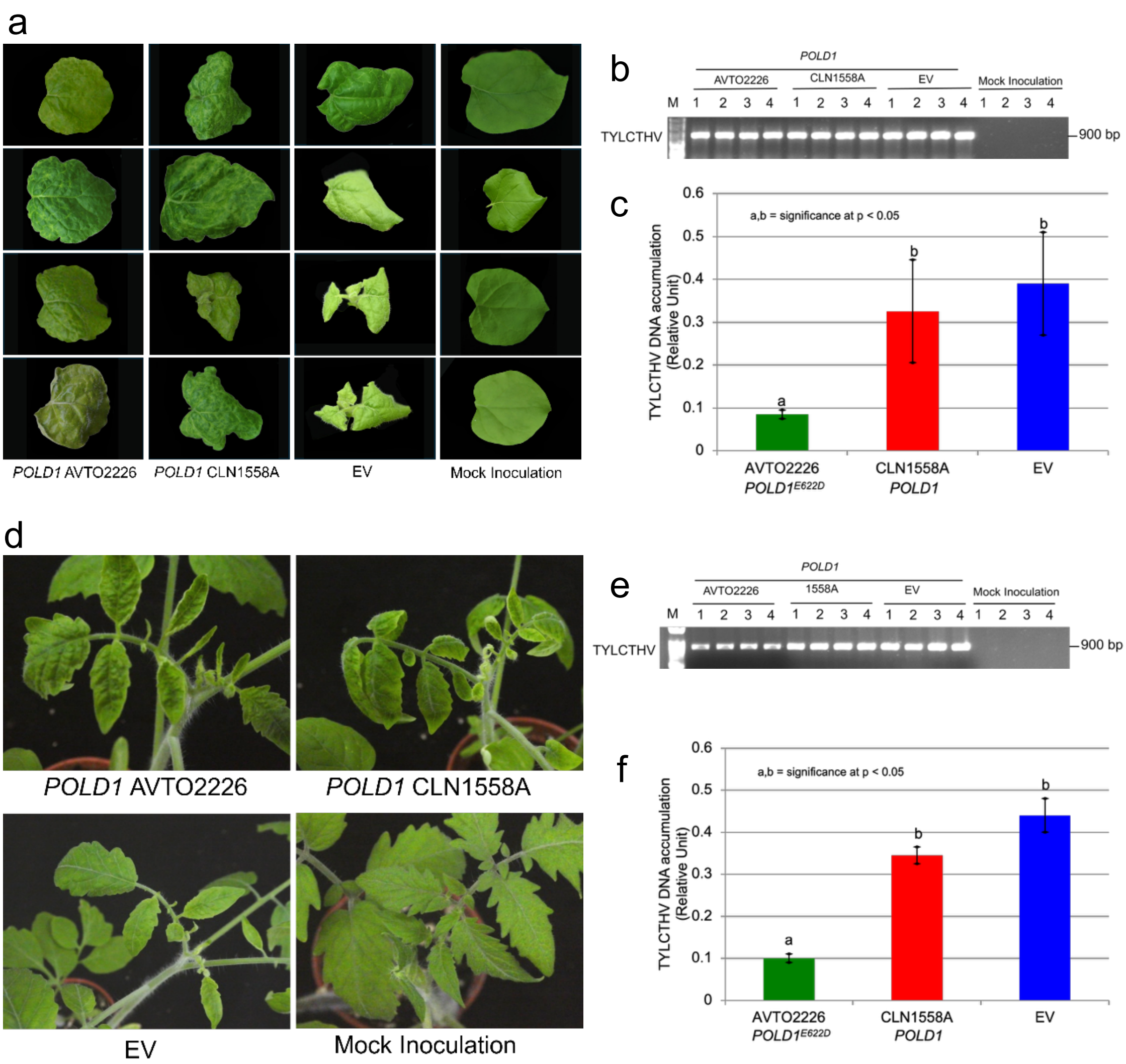

Figure S10

The effect of transient *POLD1* and *POLD1*^{E622D} cDNA expression in *N. benthamiana* and CLN1558A leaves on symptom development after inoculation with TYLCTHV. **a**. Examples of symptoms at 14 dpi on systemic leaves of *N. benthamiana* plants transiently expressing AVTO2226 *POLD1*^{E622D} or CLN1558A *POLD1* cDNAs after infection with TYLCTHV. EV = Examples of leaves agroinfiltrated with the DNA vector as a control. **b**. PCR detection of TYLCTHV in systemic leaves of *N. benthamiana* plants expresing *POLD1*^{E622D} or *POLD1* cDNAs or the DNA vector as a control (EV). **c**. TYLCTHV DNA accumulation in systemic leaves of *N. benthamiana* plants agroinfiltrated with *POLD1*^{E622D} and *POLD1* cDNAs or the DNA vector as a control (EV). **d**. Examples of symptoms on systemic leaves of CLN1558A plants transiently expressing *POLD1*^{E622D} or *POLD1* after infection with TYLCTHV at 30 dpi. EV = Examples of leaves agroinfiltrated with the DNA vector as a control. **e**. PCR detection of TYLCTHV in systemic leaves of CLN1558A plants agroinfiltrated with *POLD1*^{E622D} and *POLD1* cDNAs or the DNA vector as a control (EV). **f**. TYLCTHV DNA accumulation in systemic leaves of CLN1558A plants agroinfiltrated with *POLD1*^{E622D}

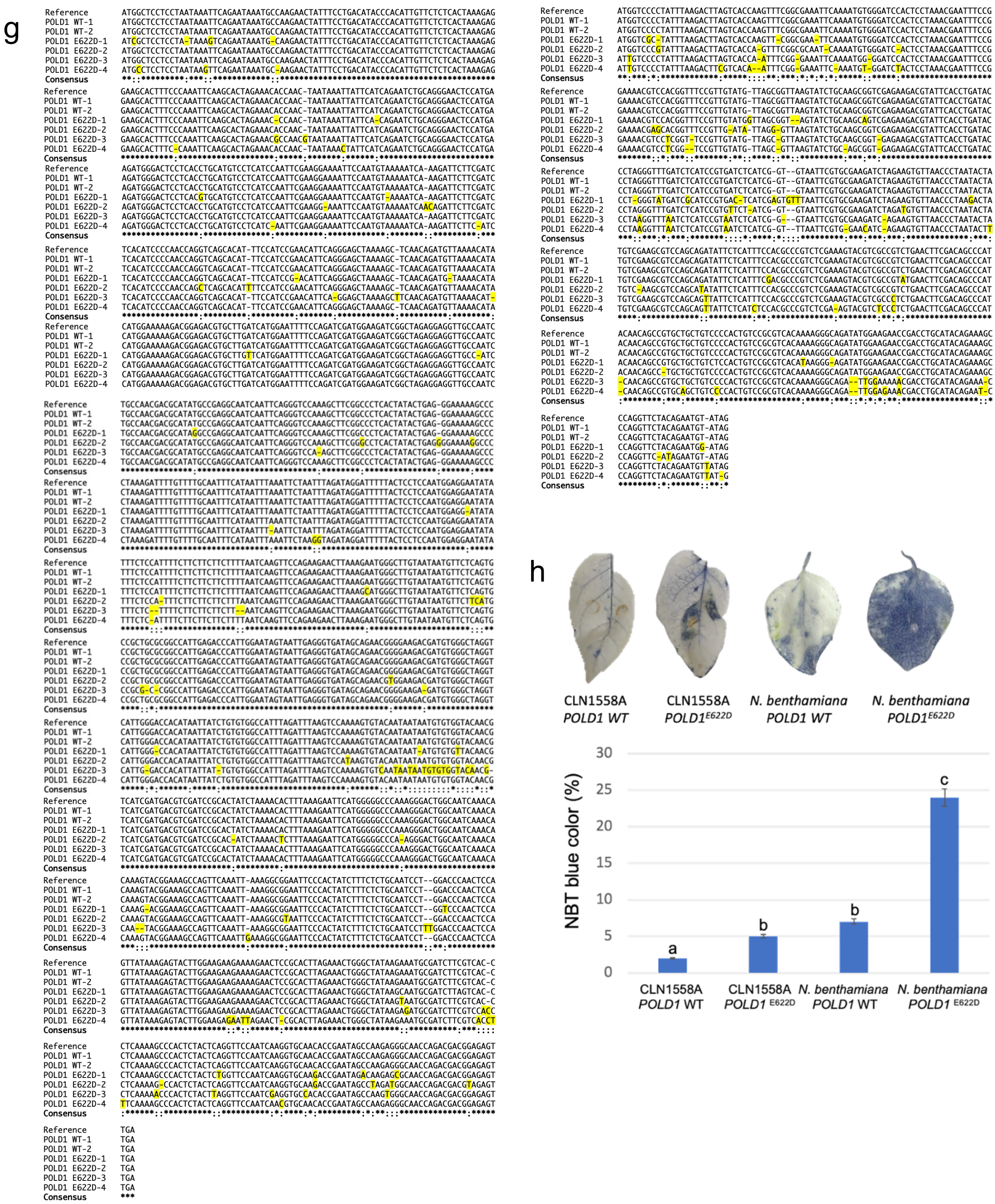

Figure S10, continued

and *POLD1* cDNAs or the DNA vector as a control (EV). **g**. Examples of *AC1* (left panel) and *AC2* (right panel) DNA sequences from TYLCTHV DNA isolated from CLN1558A leaves expressing either the *POLD1* or the $POLD1^{E622D}$ mutant allele. **h**. CLN1558A and *N. benthamiana* leaves transiently expressing the $POLD1^{E622D}$ mutant allele show an enhanced ROS response after inoculation with TYLCTHV.

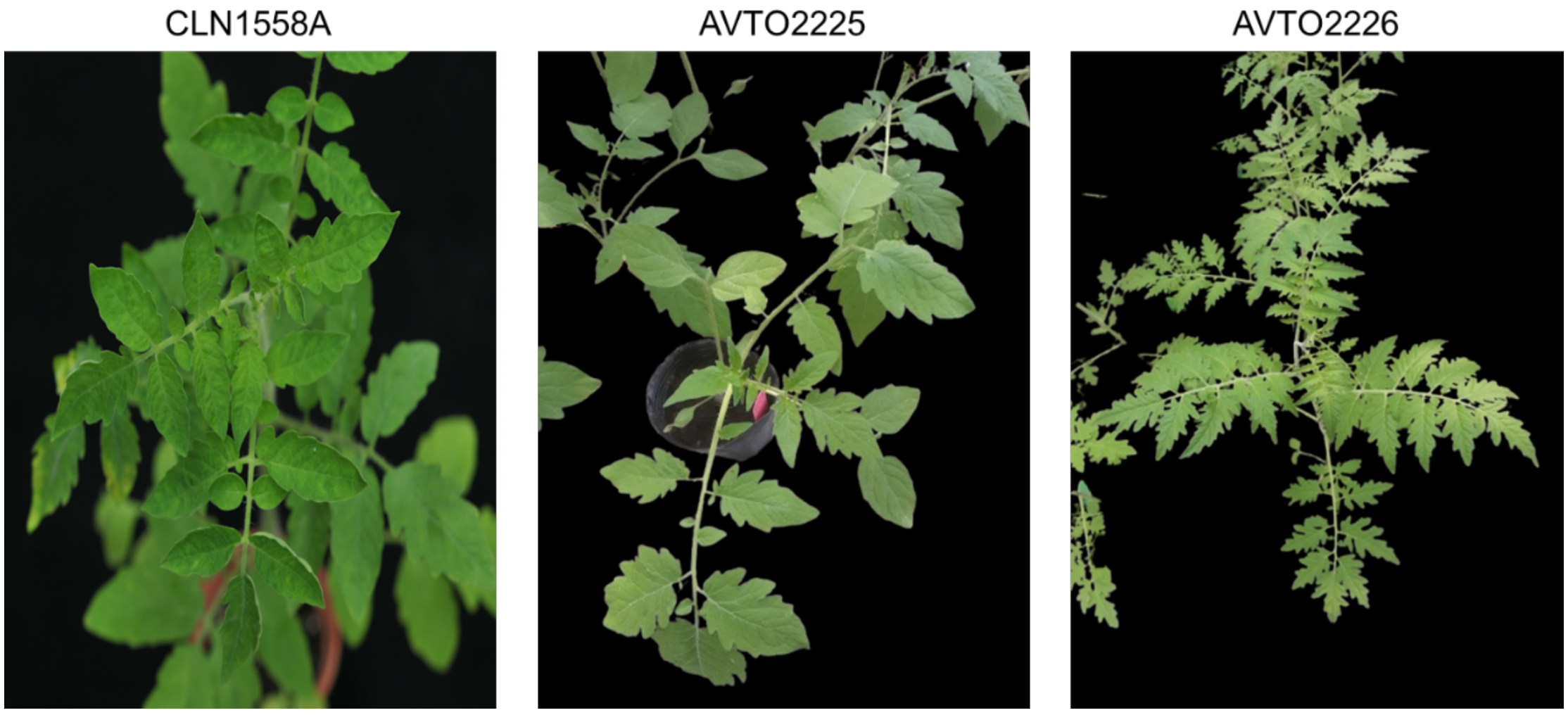

Figure S11

Leaf morphology of CLN1558A, AVTO2225, and AVTO2226 tomato plants used in this study.

Name	AVTO	Female Name	hab	Generation	pedigree	Ty1/3	Ty2	ty5	Ty6	BWr12
22AU0865 (220407-6)	AVTO2225			F5	AnsalF2-92-7-6	0	0	0	1	2
22AU0865-9		220407-6	SD	F6	AnsalF2-92-7-6-9	0	0	0	1	2
22AU0865-6		220407-6	SD	F6	AnsalF2-92-7-6-6	0	0	0	2	2
22AU0865-8		220407-6	SD	F6	AnsalF2-92-7-6-8	0	0	0	0	2
22AU0865-15		220407-6	D	F6	AnsalF2-92-7-6-15	0	0	0	2	2
22AU0865-12		220407-6	D	F6	AnsalF2-92-7-6-12	0	0	0	0	2

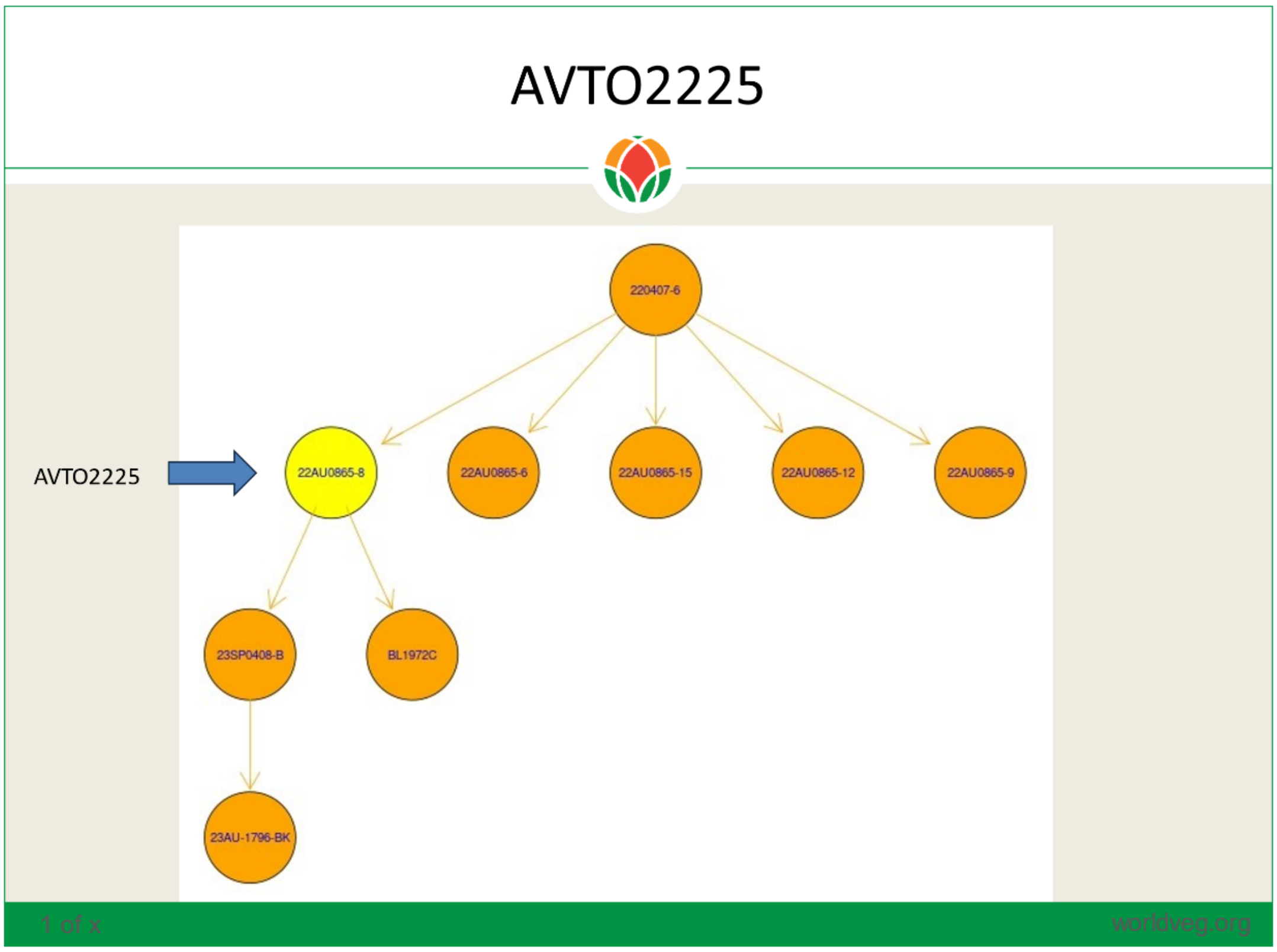

Figure S12

Ty-type resistance genotype information (top panel) and breeding pedigree of AVTO2225 (bottom panel) provided by the World Vegetable Center (WVC) in Tainan (Taiwan). The pedigree of AVTO2225 is BL1972F2-92-7. Since the exact *Ty* allele status in the AVTO2225 plants was not know, they were genotyped for *Ty-1*, *Ty-2* and *Ty-6* resistance alleles (see Table S1).

Table S2. Recoding the T1 and T2 plant codes of the AVTO2225 and AVTO2226 lines obtained from the World Vegetable Center in Tainan (Taiwan) to the numbering used in this study.

T1 code	AVTO2225	T2 code	AVTO2226
T1-7	1	T2-19	1
T1-9	2	T2-24	2
T1-21	3	T2-20	3
T1-19	4	T2-39	4
T1-8	5	T2-40	5
T1-4	6	T2-2	6
T1-5	7	T2-42	7
T1-17	8	T2-23	8
T1-24	9	T2-15	9
T1-3	10	T2-14	10
T1-22	11	T2-4	11
T1-2	12	T2-22	12
T1-1	13	T2-25	13
T1-40	14	T2-24	14
T1-39	15	T2-56	15
T1-20	16	T2-18	16
T1-23	17	T2-1	17
T1-42	18	T2-13	18
T1-34	19	T2-27	19
T1-28	20	T2-11	20

Table S3. Fruit and seed numbers obtained from assisted self-crosses of AVTO2225 and AVTO2226 parents.

AVTO2225	Seed total	Fruit total	AVTO2226	Seed total	Fruit total
1	133	3	1	96	5
2	45	2	2	58	5
3	57	2	3	0	0
4	67	3	4	0	0
5	89	3	5	0	0
6	103	4	6	0	0
7	57	2	7	24	3
8	78	4	8	0	0
9	95	4	9	39	5
10	59	3	10	0	0
11	0	0	11	53	4
12	76	2	12	0	0
13	102	3	13	87	6
14	208	4	14	0	0
15	87	2	15	43	4
16	89	2	16	0	0
17	92	2	17	0	0
18	187	4	18	65	7
19	76	2	19	0	0
20	153	3	20	0	0

Table S4. DNA primers for PCR used in this study

Primer name	Primer sequence (5'-3')	Size (bp)	Reference
Primer used for detecting the *Ty-1* and *Ty-6* CAPS and *Ty-2* SCAR markers			
F-*Ty-1*	CAATCCGGGTAGCGATCAAT	288	8
R-*Ty-1*	TCAATTTCTTCAATCAAC		
F-*Ty-2*	TGGCTCATCCTGAAGCTGATAGC	562	9
R-*Ty-2*	TATGGTTTACAGGCTAG		
F-*Ty-6*	CATAAAGTTCCGGCGAGTGT	500	4
R-*Ty-6*	TCCATTCCAAACCAAGTGAAG	200	
Primer used for qPCR analysis of TYLCTHV DNA levels			
F-TYLCTHV	TAGTTCCCCGGTGCGTAAATCCAT	962	10
R-TYLCTHV	CATCCCCAACCAGGTCAGCACAT		
Primer used for amplifying POLD1 DNA sequences			
F-*POLD1*-1	ATGAACAGCGGCAACAGCAA	1180	This study
R-*POLD1*-1	ACCTGAGCTATTGCCAGCTGG		
F-*POLD1*-2	AATTTGATTGCCTGTATAGCGAT	520	This study
R-*POLD1*-2	CGATCTGATTCGCGAAGTGGAT		
F-*POLD1*-3	CGGATATTATTATTGGC	833	This study
R-*POLD1*-3	CCAGCTGCCGTGCCTGGAAAT		
F-*POLD1*-4	TAGCAGCAGCGTGACCAGC	725	This study
R-*POLD1*-4	TGGATCGCTGGAACTTT		
Primer used for qPCR analysis of *Ty-1*, *Ty-2* (*NBS-LRR*) and *BTF3* expression			
F-qPCR *Ty-1*	ATGCAGCCAGGCTTTCCTTA	112	11
R-qPCR *Ty-1*	CAACCATTTTCATCCCGACA		
F-qPCR *Ty-2*	CCCTGCCAGCACTAGGACAGCTTTG	101	12
R-qPCR *Ty-2*	AATTCGGGACAATTTTGAATTAGGAAC		
F-qPCR BTF3	GTTGGGTACAGATCGGA	197	This study
R-qPCR BTF3	GTTTATTTTACATT		
Primer used for *AC1*, *AC2*, *AC4* and *AV2* amplification			
F-*AC1*	ATGGCTCCTCCTAATA	1086	This study
R-*AC1*	TCAACTCTCCGTCGTC		
F-*AC2*	ATGCAGTCTTCATCACA	405	This study
R-*AC2*	TTAAAGACTCGCAAGAAA		
F-*AC4*	ATGAAGATGGGACTCCTCA	301	This study
R-*AC4*	TCAGTATAGTGAGGGCCGA		
F-*AV2*	ATGGTCCCCTATTTAAGACTT	482	This study
R-*AV2*	CTATACATTCTGTAGAAC		

F = forward primer, R = reverse primer

References for Supplementary Material

1. Pérez De Castro, J. M. Blanca, M. J. Díez, F. Nuez Viñals, Identification of a CAPS marker tightly linked to the tomato yellow leaf curl disease resistance geneTy-1 in tomato. Eur. J. Plant Pathol. 117, 347–356 (2007).
2. M. G. Verlaan, et al., Chromosomal rearrangements between tomato and Solanum chilense hamper mapping and breeding of the TYLCV resistance gene Ty-1. Plant J. 68, 1093-1103 (2011).
3. B. E. Garcia, et al., Co-dominant SCAR marker for detection of the begomovirus-resistance Ty-2 locus derived from *Solanum habrochaites* in tomato germplasm. Tomato Genet. Coop. Rep. 57, 21–24 (2007).
4. U. Gill, et al., Ty-6, a major begomovirus resistance gene on chromosome 10, is effective against Tomato yellow leaf curl virus and Tomato mottle virus. Theor. Appl. Genet. 132, 1543–1554 (2019).
5. S. Sato, et al., The tomato genome sequence provides insights into fleshy fruit evolution. Nature 485, 635–641 (2012).
6. X. Su, et al., A high-continuity and annotated tomato reference genome. BMC Genomics 22, 898 (2021).
7. X. Shen, et al., The tomato gene Ty-6, encoding DNA polymerase delta subunit 1, confers broad resistance to Geminiviruses. Theor. Appl. Genet. 138, 22 (2025).
8. A. Pérez de Castro, et al., Identification of a CAPS marker tightly linked to the Tomato yellow leaf curl disease resistance gene Ty-1 in tomato. Eur J Plant Pathol 117, 347–356 (2007).
9. B. E. Garcia, et al., Co-dominant SCAR marker for detection of the begomovirus-resistance Ty-2 locus derived from Solanum habrochaites in tomato germplasm. Tomato Genet. Coop. Rep. 57, 21–24 (2007).
10. H.H. Chang, D. Gustian, C.J. Chang, F.J. Jan, Virus-virus interactions alter the mechanical transmissibility and host range of begomoviruses. Front Plant Sci. 14,1092998 (2023)
11. S. Koeda, A. Kitawaki, Breakdown of Ty-1-based esistance to Tomato Yellow Leaf Curl Virus in tomato plants at high temperatures. Phytopathology 114, 294-303 (2024)
12. S. Koeda, et al., Ty-2 and Ty-3a conferred resistance are insufficient against Tomato Yellow Leaf Curl Kanchanaburi Virus from Southeast Asia in single or mixed infections. Plant Disease 104, 3221-3229
13. M. Mubin, et al., The hypersensitive response induced by the V2 protein of a monopartite begomovirus is countered by the C2 protein. Mol Plant Pathol. 11, 245-254 (2010)
14. A. Roy, et al., Viral suppressor AC4 encoded by tomato leaf curl Palampur virus highjacks SA signaling to promote pathogenesis. Plant Physiol Biochem. 229, 110789 (2025)

Figure S7   Figure legend can be found at the end of this file at page 38

a

```
Reference     ATGGCTCCTCCTAATAAATTCAGAATAAATGCCAAGAACTA-TTTCCTGACATACCCACA
AVTO2225-R-7  ATGGGTCCTCCTAATAAATTCAGAATAAATGCCAAGAACTA-TTTCCTGACATACCCACA
AVTO2225-R-9  ATGGCTCTTCCTAATAAATTCAGAATAAATGCGAAGAACTA-GTTCCTGACATATCCACA
AVTO2225-R-11 ATGCCTCCTCCTAATAAATTCAGAATAAATGCCAAGAACTACCTTCCTGACATACCCACA
              ***  **.************************ ********  ***********.*****

              TTGTTCTCTCACTAAAGAGGAAGCACTTTCCCAAATTCAAGCACTAGAAACACCAACTAA
              TTGTTCTCTCACTAAAGAGGAAGCAC-TTCCCAAATTCAAGCACTAGAAACACCAACTAA
              TTGTTCTCTCACTAAAGAGGAAGCACTTTCCCAAATTCAAGCACTAGAAACACCAACTAA
              TTGTTCTCTCACTAAAGAGGAAGCACTTTCCCAAATTCAAGCACTAGAAACACGAACTAA
              ************************** ************************** ******

              TAAA-TTATTCATCAGAATCTGCAGGGAACTCCATGAAGATGGGACTCCTCACCTGCATG
              TAAATTTATTCATCAGTATCTGCAGGGAACTCCATGAAGATGGGACTCCGCACCTGCATG
              TAAA-TTATTCATCAGAATCT--AGGGAACTCCATG-AGATGGGACTCCTCACCTGCATG
              TAAA-T--TTCATCAGAATCTGCAGCGAACTCCATGAAGATGGGACTCCTCACCTG-ATG
              **** *  ******** ****  ** ********** ************ ****** ***

              TCCTCATCCAATTCGAAGGAAAATTCCAATGTAAAAATCAAAGATTCTTCGATCTCACAT
              TCCTCATCCAGTTCGAAGGAAAA-TCCAATGTAAAAATCAAAGATTCTTCGATCTCACAT
              TCCTCCTCCAATTCGAAGGAAAATTCCAATFTAAAATTCAAAGATTCTTCGATCTCTAAT
              TCCTCATCCAATTCGAAGGAAAATTCCAATGTAAAAATCAAAGATTCTTCGATC-CACAT
              ***** ****.************ ****** ***** ***************** *  **

              CCCCAACCAGGTCAGCACATTTCCATCCGAACATTCAGGGAGCTAAAAGCTCAACAGATG
              CCCCAACCAGGTCAGCACATTTCGATCCGAACATTGAGGGAGCTAAAAGCTCAACAGGTG
              CCCCAACCAGGTCAGCACATTTCCATCCGAACATTCAGGGAGCTAAAAG-TCAACAGA--
              CCCCAACCAGGTCAGCACATTTCCATCCGAACATTCAGGGAGCTAAAAGCTCAACAGATG
              *********************** *********** ************* *******.

              TTAAAACATACATGGAAAAAGACGGAGACGTGCTTGATCATGGAATTTTCCAGATCGATG
              TTAAAACATACATGG---AAGATGGAGACGTGCTTG-TCATGGAATTTT----ATCGATG
              GTTAAACATACATGGAAAAAGACGGAGACTGTTTGA-TCATGGATTTTTCCACATCGATG
              TTAAAACATACA-GGAAAAAGACGGAGACGCTTCT----ATCTGGTTTTCCAGATCGATC
               * ********* **   ****.******   ..     **  . ****    ******

              GAAGATCGGCTAGAGGAGGTTGCCAATCTGCCAACGACGCATATGCCGAGGCAATCAATT
              GAAGATCGGCTAGAGGAGGTTGCCAATCTGCCAACGACGCATATGCCGAGGCAATCAATT
              GAAGATCGGCTAGAGGAGGGTGCCAATCTGCCA--CGACCATATGCCGAGGCAATCAGTT
              GAA------CTAGAGGAGGTTGCCAATCTGCCAACGA--CATATGCCGAGGCATTCAATT
              ***      ********** *************   .  ************** ***.**

              CAGGGTCCAAAGCTTCGGCCCTCACTATACTGAGGGAAAAAGCCCCTAAAGATTTTGTTT
              CAGGGTCCAAAGCTTCGGCCCTCACTATACTGAGGGAAAAAGCCCCTAAAGATTGTTAAT
              CAGCGTCCAAAGCTTCGGCCCTCACTATACTGAGGGAAAAAGCCCCTAAAGATTTTGTTT
              CAGGGTCCAAAGCTTCGGCCCTCACTATACTGAGGGAAAAAGCCCCTAAAGATTCCCTTA
              *** ************************************************** .

              TGCAATTTCATAATTTAAATTCTAATTTAGATAGGATTTTTACTCCTCCAATGGAGGAAT
              TGCAATTTCATAATTTAAATTCTAGTTTAGATAGGATTTTTACTCCTCCAATGGAGGAAT
              TGCAATTTCATAATATAAATTCTAATTTAGATACGA--TTTACGCCTCGAATGGAGGAAT
              TGCAATTTC---AATTTAATTCTAATTTAA--TAGATTTTTACTCCTCCAATGGAGGAAT
              *********   *  * *******.****.    **  ***** **** ***********

              ATATTTCTCCA-----TTTTCTTCTTCTTCTTTTAATCAAGTTCCAGAAGAACTTAAAGA
              ACATCTCTCCA-----TTTTCTTCTTCTTCTTTTAATCAAGTTCCAGAAGAACTTAAAGA
              ATATTTCTCCA-----TTTTCTTCTTCTTCTTATAA-CAAGTTCCAGAAGAACTTAAAGA
              ATATTTCTCCATAATTTCCACTTCTTCTTATTTTTATCAAGTTCCAGAAGAACTTAAAGA
              *.**.******     *.. ********* ** * * ***********************
```

```
Reference        ATGGGCTTGTAATAATGTTCTCAGTGCCGCTGCGCGGCCATTGAGACCCATTGGAATAGT
AVTO2225-R-7     ATGGGCTTGTAATAATGTTCTCAGTGCCGCTGCGCGGCCAATGAGACCTATTGGAATTGT
AVTO2225-R-9     ATGGGCTTGTAATTATGTTCTCAGTGCCGCTGCGCGGCCGTTGAGACCCATTGGAATAGT
AVTO2225-R-11    ATGGGCTTGTAATAATG-TCTCAGTGCCGCTGCGCGGCCATTGAGTCCTGTTGGAATAGT
                 ************* *** *********************. **** **..******* **

                 AATTGAGGGTGATAGCAGAACGGGGAAGACGATGTGGGCTAGGTCATTGGACCACATAA
                 AATTGAGGGTGATAGCAGACGG--------GATGTGGGCTAGGTCATTGGACCACATAA
                 AATTGTGGGTGATAGCAGAACGGCGAAGACGATGTGGGCT-GGTCATTGGACCACATAA
                 AATTGAGGGTGAAAGC-GAACGGGGAAGACGATGTGGGCTAGGTCATTGGACCACATAA
                 ***** ****** *** **  *        ********** ******************

                 TTATCTGTGTGGCCATTTAGATTTAAGTCCAAAAGTGTACA-ATAATAATGTGTGGTACA
                 TTATCTGTGTGGCCGTTTAGATTTAAGTCCAAAAGTGTACA-ATAA--ATGTGTGGTACA
                 TTATCTGTGTGGCCATTTAGA-TTAAGTCCAAAAGTGTACAAATAATAATGTGTGGTACA
                 TTATCTGTGTGGCCATTTAGATTTAAGTCCAAAAATGTACA-ATAA-----TATGTGCTA
                 **************.****** ************.****** ****     *.**   .*

                 ACGTCATCGATGACGTCGATCCGCACTATCTAAAACACTTTAAAGAATTCATGGGGGCCC
                 ACGTCATCGATGACGTCGATCCG------CTAAAACACTTTAAAGAGTTCATGGGGGCCC
                 ACGTCATCGA-GACGTCGATCCGCACTATCTAAAACACTTTAAAGAATTCATGGGGGCCC
                 CCGTCATCGATGACGTCGAAAAGCACT---TAAAACACCTTAAAGAATTCATGGGGGCCC
                  ********* ********   *       ********.*******.*************

                 AAAGGGACTGGCAATCAAACACAAAGTACGGAAAGCCAGTTCAAATTAAAGGCGGAATTC
                 AAAGGGACTGGCAATCAAACACAAAGTACGGTAAGCCAGTTCAAATTAAAGGCGGAATTC
                 AAAGCGACTGGCAATCAAACACAAGGTACGGAAAGCCAGTTCAAATTAAAGGCGGAATTC
                 AAAGGGACTGGCAATCAAACACATAGTACGGAAAGCCAGTTCAAATTAAAGGCGGAATTC
                 **** ****************** .****** ****************************

                 CCACTATCTTTCTCTGCAATCCTGGAC-CCAACTCCAGTTATAAAGAGTACTTGGAAGAA
                 CCACTATCTTTCTCTGCAACCCTGGAC-CCAACTCCAGTTATAAGGAGTACTTGGAAGAA
                 CCGCTATCTTTCTCTGCAATCCTGGACGCCAACTCCAGTTATAAAGAGTACTTGGAAGAA
                 CCACTAGGTTTCTCTGCAATCCTGGAC-CCAACTCCAGTTATCAATAGTACTTGGAAGAA
                 **.***  ***********.******* ************** *. **************

                 GAAAAGAACTCCGCACTTAGAAACTGGGCTATAAGAAATGCGATCTTCGTCACCCTCAAA
                 GAAAAGAACTCCGCACTTAGAAACTGGGCTATAAGAAATGCGATCTTCGTCACCCTC-AA
                 GAAAACAACTCCGCACTTAGAAACTGGGCTATAAGAAATGCGATCTTCGTCACCCTCAAA
                 GAAAAGAACTCCGCAC-TAGAAACTGGGCTATAAGAAATGCGATAATCGTCACCCTCAAA
                 ***** ********** ***************************  *********** **

                 AGCCCACTCTACTCAGGTTCCAATCAAGGTGCAACACCGAATAGCCAAGAGGGCAACCAG
                 AGCCCACTCTACTCAGGTTCCAATCAAGGTGCAACACCGAATAGCGAAGAGGGCAACCAG
                 AGCCCACTCTACTCAGGTTCCAATCAAGGTGCAACACCGAATAGCCAAGAGGGCAACCAG
                 AGACCACTGTACTCAGGTTCCAATCAAGGTGCAATTCCGAATAGCCAAGAGGCCAACCAG
                 ** ***** *************************. ********* ****** *******

                 ACGACGGAGAGTTGA
                 ACGACGGAGAGTTGA
                 ACGAGGGAGAGTTGA
                 ACGACGGAGAGTTGA
                 **** **********
```

b

```
Reference        ATGCGATCTTCGTCACCCTCAAAAGCCCACTCTACTCAGGTTCCAATCAAGGTGCAACAC
AVTO2225-R-7     ATGCGATC-TCGTCACCCTCAAAAGCCCAC-CTACTCAGGTTCCAATCAAGGTGCAACA-
AVTO2225-R-9     ATGCGATCTTC-TCACCCTCAAAAGCCCACTCTACTCAGGTTCCAATC-AGGTGCAACAC
AVTO2225-R-11    ATGCGATCTTCGTCACCCTCAATAGCCCACTCTACTCAGGTTCCAATCAAGGTGCAACAC
                 ******** ** ********** ******* ***************** **********

                 CGAATAGCCAAGAGGGCAACCAGACGACGGAGAGTTGATCTACCTTGTGGATGTTCGTAC
                 -GAATAG-CAAGAGGGCAACCAGACGACGGAGAGTTGATCTACCTTGTGGATGTTCGTAC
                 --AATAGCCAAGAGGGCAACCAGACGACGGAGAGTTGATCTACCTTGTGGATGTTCGTAC
                 CGAATAGCCAAGAGGGCAACCAGACGACGGAGAGTTGATCTACCTTGTGGGTGTTCGTAC
                   ***** ******************************************.*********

                 TTCGTCGCAATAGGATGTC-ACAATAATGGATTTACGCACCGGGGAACTACTCACTGCAA
                 TTCGTCGCAAGAGGATGTC-AC-ATAATGGATTTACGCACC-GGTAACTACTCACTGCAA
                 TTCGTCGCAATAGGATGTC-ACCATAATGGATTTACGGCCG-GGGAACTACTCACTGCAA
                 CTCGTCGCAATACGATGTCTGCAACAATGGATTTACGCACC-GGGGAAC-CTCACTGCAA
                 .********* * ****** .* *.************   *   ** .* .  **********

                 CTCAATTAGAGAGTGGCGTGTATATCTGGACGCTCAAAAATCCCCTATATTTCAAGATAA
                 CTCAATTAGAGAGTGACGTGTATATCTGGACGCTCAAAAATCCCCTATATTTCAAGATAA
                 CTCAATTAGAGTGTGGCGTGTATATCTGGACGCT-AAAAATCCCCTATATTTCAAGATAA
                 CTCAATTAGAGAGTGGCGTG-ATATCTGGACGCTCGAAAATCCCCTATATTACAAGATAA
                 *********** ***.**** ************* .*************** ********

                 CCAAGCACCTAGAGAGCCCATTCCAGAGGAACCACGACATAATCACGTTACAAATCCAGT
                 CCAAGCTCCTAGAGAGCCCATTCCAAGGGAACCACGACATAATCACGTTAC-AATCCAGT
                 CCAA-CACCTAGAGAGCCCATTGCAGAGGAACCACGAGATAATCACGTTACAATTCCAGT
                 CCAG--CACCAGAGAGCCCA-TCCAGAGGAACCACGACATTATCACGTTACAAATCCAGT
                 ***.     *.********** * **..********** ** ********** * ******

                 TCAACCACAACCTGAGGAAAGCGTTGGGGATACACAAATGTTTTCTAGTCTGCAAAATCT
                 TCAACCACAACCTGAGGAAAGCGTTGGGGATACACAAATGTTTTCTAGTCTGCAAAATCT
                 TCAACCACAACCTGAGGAAAGCGTTGGGGATAGACAAATGTTTTCTAGTCTGCAAAATC-
                 TCAACCACAACCTGAGGAAAGCGTTGGGTATACAC-AATGTTTTCTAGTCTGCAAAATCT
                 **************************** *** ** **********************

                 GGACTCATTTACATCCTCAGACCTCGCGTTTCTTAACAGTATTTAA
                 GGACTCATTTACATCCTCGGACCTCGCGTTTCTTAACAGTATTTAA
                 GGACTCATTTACAGCCTCAGACCTCGCG-TTCTTAACAGTATTTAA
                 GGACTCATTTACATCCTCAGACC-CGCGTTTCTTAACAGTATTAA-
                 ************* ****.**** **** ************** *
```

C

```
Reference      A--TGGCTCCTCCTAATAAATTCAGAATAAATGCCAAGAACTATTTCCTGACATACCCAC
AVTO2226-2     A--TGGCTCCTCCTAATAAATTCAGAATAAATGCCAAGAACTATTTCCTGACATACCCAC
AVTO2226-3     T--GGGCTCCTCCTAATAAATTCAGAATAAATGCCAAGAACTATTTCCTGACATACCCAC
AVTO2226-4     -----ACTCCTCCTAATAAATTCAGAATAAATGCCAAGAACTATTTCCTGACATACCCAC
AVTO2226-5     G--TGGCTCCTCCTAATAAATTCAGAATAAATGCCAAGAACTATTTCCTGACATACCCAC
AVTO2226-6     -----GCTCCTCCTAATAAATTCAGAATAAATGCCAAGAACTATTTCCTGACATACCCAC
AVTO2226-7     ----AGCTCCTCCTAATAAATTCAGAATAAATGCCAAGAACTATTTCCTGACATACCCAC
AVTO2226-8     ATTTTGCTCCTCCTAATAAATTTTGAATAAATGCCAAGAACTATTTCCTGACATACCCAC
AVTO2226-9     ---AGGCTCCTCCTAATAAATTCAGAATAAATGCCAAGAACTATTTCCTGACATACCCAC
AVTO2226-10    A-TTGGCTCCTCCTAATAAATTGAGAATAAATGCCAAGAACTATTTCCTGACATACCCAC
AVTO2226-11    ----ACGGCTCCCTAATAAATTCAGAAT-AATGCCAAGAACTATTTCCTGACATACCCAC
AVTO2226-12    ---ATGGCCTCCCTAATAAATTCAGAAT-AATGCCAAGAACTATTTCCTGACATACCCAC
                       *..***********  **** ******************************

               ATTGTTCTCTCACTAAAGAGGAAGCACTTTCCCAAATTCAAGCACTAGAAACACCAACTA
               ATTGTTCTCTCACTAAAGAGGAAGCACTTTCCCAAATTCAAGCACTAGAAACACCAACTA
               ATTGTTCTCTCACTAAAGTGGAAGCACTTTCCCAAATTCAAGCACTAGAAACACCAACTA
               ATTGTTCTCTCACTAAAGAGGACCCACTTTCCCAAATTCAAGCACTAGAAACACCAACTA
               ATTGTTCTCTCACTAAAGAGGAAGCACTTTCCCAGATTCAAGCACTAGAAACACCAACTA
               ATTGTTCTCTCAC--AAGAGGAAGCACTTTCCCAAATTCAAGCACTAGAAACACCAACTA
               ATTGTTCTCTCACTAAAGAGGAAGCACTTTCCCAAATTCAAGCACTAGAAACA-CAACTA
               ATTGTTCTCTCACTAAAGAGGAAGCACTTTCCCAAATTCAAGCACTAGAAACACCAACTA
               ATTGTTCTCTCACTAAAGAGGAAGCACTTTCCCAAATTCAAGCACTAGAAACACCAACTA
               ATTGTTCTCTCACGTAAGAGGAAGCACTTTCCCAAATTCAAGCACTAGAAACACCAACTA
               ATTGTTCTCTCACTAAAGAGGAAGCAC---------------------------------
               ATTGTTCTCTCACTAAAGAGGAAGCAC---------------------------------
               *************  *** ***  ***

               ATAAATTATTCATCA---GAATCTGCAG-GGAACTCCATGAAGATGG-GACTCCTCACCT
               ATAAATTATTCATCA---GAATCTGCAG-GGAACTCCA-GAAGATGG-GACTCCTCACCT
               ATAAATTATTCACCA---GAATCTGCAG-GGAACTCCATGAAGATGG-GACTCCTCACCT
               ATAAATTATTCATCA---GAATCTGCAG-GGAACTCCATGAAGATGG-GACTCCTCACCT
               ATAAATTATTCATCA---GAATCTGCAG-GGAACTCCATGAAGATGG-GACTCCTCACCT
               ATAAATTATTCATCA---GAATCTGCAG-GGAACTCCATGAAGATGG-GACTCCTCACCT
               ATAAATTATTCATCA---GAATCTGCAGCGCAACTCCATGAAGATGG-GACTCCTCACCT
               ATAAATTATTCATCA---GAATCTCCAG-GGAACTCCATGAAGATAACCACTCCTCACCT
               ATAAATTATTCATCATTTAAATCTGCAG-GGAACTCCATGAAGATGG-GACTCCTCACCT
               ATAAATTATTCATCA---GAATCTGCAG-GGAACTCCATGAAGATGG-GACTCCTCACC-
               ------TATTCATCA---GAATCTGCAG-GGAA-TCCATGAAGATGG-GACTCCTCACCT
               ------TATTCATCA---GAATCTGCAG-GGAA-TCCATGAAGATGG-GACTCCTCACCT
                     ******.**    .***** *** * ** **** ******..  **********

             GCATGTCCTCATCCAA--TTCGAAGGAAAATTCCAATGTAAA---AATCAAAGATTCTTC
             GCATGTCCTCATCCAA--TTCGAAGGAAAATTCCAATGTAAA---AATCAAAGA-TCTTC
             GCATGTCCTCATCCAA--TTCGAAGGAAAATTCCAATGAAAA---AATCAAAGATTCTTC
             GCATGTCCTCATCCAA--TTCGAAGGAAAATTCCAATGTTAA---AATCAAAGATTCTTC
             GCATGTCCTCATCCAA--TTCGAAGGAAAACTCCAATGTAAA---AATCAAAGATTCTTC
             GCATGTCCTCATCCAA--TTCGAAGGAAAATTCCAATGTAAA---AATCAAAGATTCTTC
             GCATGTCCTCATCCAA--TTCGAAGGAAAATTCCAAT---AA---AATCAAAGATTCTTC
             GCATGTCCTCATCCAA--TTCGAAGGAAAATTCCAATGTAAA---AATCAAAGATTCTTC
             GCATGTCCTCATCCAA--TTCGAAGGAAAATTCCAATGTAAA---AATCAAAGATTCTTC
             -----TCCTCATCCAATCTTCGAAGGAAAATTCCAATGTAGGGCCAATCAAAGATTCTTC
             GCATGTCCTCATCCAA--TTCGAAGCCAAATTCCAATGTAAA---AATCAAAGATTCTTC
             GCATGTCCTCATCCAA--TTCGAAGCCAAATTCCAATGTAAA---AATCAAAGATTCTTC
                  ***********  *******  ***.******    ..   ********* *****
```

```
Reference      GATCTCACATC-CCCAACCAGGTCAGCACATTTCCATCCG--AACATTCAGGGAGCTAAA
AVTO2226-2     GATCTCACATC-CCCAACCAGGTCAGCACATTTCCATCCG--AACATTCAGGGAGCTAAA
AVTO2226-3     GATCTCACATC-CCCAACCAGGTCAGCACATTTCCATCCG--AACATTCAGGGAGCTAAA
AVTO2226-4     GATCTCACATC-CCCAACCAGGTCAGCACATTTCCATCCG--AACATTCAGGGAGCTAAA
AVTO2226-5     GATCTCACATC-CCCAACCAGGTCAGCACATTTCCATCAG--AACATTCAGGGAGCTAAA
AVTO2226-6     GATCTCACATC-CCCAACCAGGTCAGCACATTTCCATCCGAAAACATTCAGGGAGCTAAA
AVTO2226-7     GATCTCACATC-CCCAACCAGGTCAGCACATTTCCATCAA--AACATTCAGGGAGCTAAA
AVTO2226-8     GATCTCACATC-CCCAACCAGGTCAGCAC--TTCCATCCG--AACATTCAGGGAGCTAAA
AVTO2226-9     GATCTCACATTAACCAACCAGGTCAGCACATTTCCATCCG--AACATTCAGGGAGCT-AA
AVTO2226-10    GATCTCACATC-CCCAACCAGGTCAGCACATTTCCATC------CATTCAGGGAGCTAAA
AVTO2226-11    GATCTCACATC-CCCAACCAGGTCAGCACATTTCCATCCG--AACATTCAGGGAGCTAAA
AVTO2226-12    GATCTCACATC-CCCAACCATGTCAGCACATTTCCATCCG--AACATTCAGGGAGCTAAA
               **********.  ******* ********  *******      ************* **

               AGC--TCAACAGA--TGTTAAAACATACATGGAAAAAGA--CGGAGACGTGCTTGATCAT
               AGC--TCAACAGA--TGTTAAAACATACATGGAAAAAGA--CGGAGACGTGCTTGATCAT
               AGC--TCAACAGA--TGTTAAAACATACATGGAAAAAGC--CGGAGACGTGCTTGATCAT
               AGC--TCAACAGA--TGTTAAAACATACATGGAAAAAGA--CGGAGACGTGCTTGATCAT
               AGC--TCAACAGA--TGTTAAAACATACATGGAAAAAGA--CGGAGACGTACTTGATCAT
               AGC--TCAACAGA--T-----AACATACATGGAAAAAGA----GAGACGTGCTTGATCAT
               AGC--TCAACAGA--TGTTAAAACATACATGGAAAAAGA--CGGAGA--TGCTTGATCAT
               AGC--TCAACAGA--TGTTAAAACATACATGGAAAAAGA--CGG---CGTGCTTGATCAT
               AGC--TCAACAGATTTGTTAAAACATACATGGAACAGGA--CGGAGACGCGCTTGATCA-
               AGCAATTAACAGA--TGTTAAAACATACATGGAAAAAGA--CGGAGACGTGCTTGATCAT
               AGC--TCAACAGA--TGTTAAAACATACATGGAAAAAGAGGCCGAGACGTGCTTGATCAT
               AGC--TCAACAGA--TGTTAAAACATACA-GGAAAAAGAGGCCGAGACGTGCTTGATCAT
               ***  *.******  *     ******** **** *.*     *     ..********

               GGAATTT-TCCA---GATCGATGGAAGATCGGCT--AGAGGAGGTTGCCAATCTGCC---
               GGAATTT-TCCA---GATCGAT--AAGATCGGCT--AGAGGAGGTTGCCAATCTGCC---
               GTAATTT-TCCA---GATCGTTGGAAGATCGGCT--AGAGGAGGTTGCCAATCTGCT---
               GGAATTT-TCCA---GATCGATGGAAGATCGGTTAAAGAGGAGGTTGCCAATCTGCTTAA
               GGAATTT-TCCA---GATCGATGGAAGATCGGCT--AGAGGAGGTTGCCAA---GCC---
               GGAATCCGTCCA---GATCGATGGAAGATCGGCT--AGAGGAGGTTGCCAATCTGCC---
               GGAATTT-TCCA---GATCGATGGAAGATCGGCT--AGAGGAGGTTGCCAATGTGCCC--
               GGAATTT-TCCACCGGAACGATGGAAGATCGGCT--AGAGGAGGTTGCCAATCTGCC---
               ------------------------------------------------------------
               GGAATTT-TCCA---GATCGATGGAAGATCGGCT--AGAGGAGGTTGCCAATCTGCCA--
               GGAATTT-TCCA--GCATCGATGGAAGATCGGCT--AGAGGAGGTTGCCAATCTGCCCC-
               GGAATTT-TCCA--GCATCGATGGAAGATCGGCT--AGAGGAGGTTGCCAATCTGCCCC-

               AACGACGCAT--ATG--CCGAGGCAATCAATTCAGGGTCCAAAGCTTCGGCCCTCAC-TA
               AACGACGCAT--ATG--CCGAGGCAATCAATTCAGGGTCCAAAGCTTCGGCCCTCAC-TA
               AACGACGCAT--ATG--CCGAGGCCATCAATTCAGGGTCCAAAGCTTCGGCCCTCAC-TA
               AACGACGCAT--GGG--CCGAGGCAATCAATTCAGGGTCCAAAGCTTCGGCCCTCAC-TA
               AACGACGCAT--ATG--CCGAGGCAATCAATTCAGGGTCCAAAGCTTCGGCCCTCAC-TA
               AACGACGCAT--ATGCCCCGAGGCAATCAATTCAGGGTCCAAAGCTTCGGCCCTCAC-TA
               GACGACGCAT--GCT--GTCAGGCAATCAATTCAGGGTCCAAAGCTTCGGCCCTCAC-TA
               AACGACGCAT--ATG--CCGAGGCAATCAATTCAGGGTCCAAAGCTTCGGCCCTCAC-TA
               -------------------AAGGCAATCAATTCAGGGTCCAAAGCTTCGGCCCTCAC-TA
               GCCGACGCAT--ATG--CCGAGGC------TTCAGGGTCCAAAGCTTCGGCCCTCAC-TA
               AACGACGCATAAATG--CCGAGGCAATCAATTCAGGGTCCAAAGCTTCGGCCCTCACTTA
               AACGACGCATAAATG--CCGAGGCAATCAATTCAGGGTCC-AAGCTTCGGCCCTCACTTA
                                    ****      ********* **************** **
```

```
Reference      TACTGAGGGAAAAAGCCCCTA--AAGATTTTGTTTTGCAATTTCATAATTTAAATTCTAA
AVTO2226-2     TACTGAGGGAAAAAGCCCCTA--AAGATTTTGTTTTGCAATTTCATAATTTAAATTCTAA
AVTO2226-3     CACTGAGGGAAAAAGCCCCTA--AAGA---TGTTTTGCAATTTCATAATTTAAATTCTAA
AVTO2226-4     TACTGAGGGAAAAA-CCCCTA--AAGATTTTGTTTTGC-ATTTCATAATTTAAATTCTAA
AVTO2226-5     TACTGA--GAAAAAGCCCCTA--AAGATTTTGTTTTGCAATTTCATAATTTAAATTCTAA
AVTO2226-6     TACTGAGGGAAAAAGCCCCTA--AAGA-------TTGCAATTTCATAATTTAAATTCTAA
AVTO2226-7     TACTGAGGGAAAAAGCCCCTA--AAGATTTTGTTTTGCAATTTCATAATTTAAATTCTAA
AVTO2226-8     TACTGAGGGAAAAAGCCCCTA--AAGATTTTGTTTTGCAATTTCATAATTTAAATTCTAA
AVTO2226-9     TACTGAGGGAAAAAGCCCCTA--AAGATTTTGTTTTGCAATTTCATAATTTAAATTCTAA
AVTO2226-10    TACTGAGGGAAAAAGCCGCTATTAAGATTTTGTTTTGCAATTTCATAATTTAAATTCTAA
AVTO2226-11    AAGACTGAGGGAAAGCCCCTA--AAGATTTTGTTTTGCAATTTCATAATTTAAATTCTAA
AVTO2226-12    AAGACTGAGGGAAAGCGCCTA--AAGATTTTGTTTTGCAATTTCATAATTTAAATTCTAA
                *      *..*** *   ***   ****         **** *********************

               TTTAGATAGG--ATTTTTACTCCTCCAATGGAGGAATATATTTCTCCATTTTCTTCTTCT
               TTTAGATAGG--ATTTTTGCTCCTCCAATGGAGGAATATATTTCTCCATTTTCTTCTTCT
               TTTAGATAGG--ATTTTTACTCCTCCAATGGAGGAATATATTTCTCCATTTTCTTCTTCT
               TTTAGATAGG--ATTTTACTCCTCCATTTGGAGGAATATATTTCTCCATTTTC-TCTTCT
               TTTAGATAGC--GATTTT----CTCCAATGGAGGAATATATTTCTCCATTTTCTTCTACT
               TTTAGATAGG--ATTTTTACTCCTCCAATCGAGGAATATATTTCTCCATTTTCTTCTTCT
               TTTAGATAGG--ATTTTTACTCCTCCAATTGAGGAATAT-TTTCTCCATTTTCTTCTTCT
               TTTAGATAGG--ATTTTTACTCCTCCAATGG-----------------------------
               TTTAGATAGG--ATTTTTACTCCTCGAATGGAGGAATATATTTCTCCATTTTCTTCTTCT
               TTTAGATAGG--ACCGGTTACTCCTCAATGGAGGAATATATTTCTCCATTTTCTTCTTCT
               TTTAGATAGGAAATTTTTACTCCTCCAATGGAGGAATACCTTTCTCCATTTTCTTCTTCT
               TTTAGATAGGAAATTTTTACTCCTCCAATGGAGGAATACCTTTCTCCATTTTCTTCTTCT
               *********    . .        ...    * *

               TCTTTTAATCAAGTT-CCAGAAGAACTTA----AAGAATGGGCTTGTAATAATGTTCTCA
               TCTTTTAATCAAGTT-CCAGACGAACTTA----AAGAATGGGCTTGTAATAATGTTCTCA
               TCTTTTAATCAAGTT-CCAGAAGAACTTA----AAGAATGGGCTTGTAATAATGTTCTCA
               TCTTTTAATCAAGTT-CCAGAAGAACTTA----AAGAATGGGCTTGTAATAATGTTCTCA
               TCTTTTAATCAAGTT-CCAGAAGAACGTA----AAGAATGGGCTTG------TGTTCTCA
               TCTTTTAATCAAGTT-CCAGAAGAACTTA----AAGAATGGGCTTGTAATAATGTTCTCA
               TCTTTTAATCAAGTT-CCAGAAGAACTTA----AAGAATGGGCTTGTAATAATGTTCTCA
               ----------------------------------------------------------CA
               TCTTTTAATCAAGTT-CCAGAAGAACTTA----AAGAATGGGCTTGTAATAATGTTCTCA
               TCTTTTAATCAAGTT-CCAGAAGAACTTACCGGAGAATCGCGCTTGTAATAATGTTCTCA
               TCTTTTAATCAAGTTAACAGAAGAACTTA----ACGCATGGGCTTGTAATAATGTTCTCA
               TCTTTTAATCAAGTTAACAGAAGAACTTA----ACGCATGGGCTTGTAATAATGTTCTCA
                                                                         **

               GTGCCGCTGCGCGGCCATTGAGACCCATTGGAATAGTAATTG--AGGGTGATAGCAGAAC
               GTGCCGCTGCGCGGCCATTGAGACCCATTGGAATAGTAATTG--AGGGTGA-AGCAGAAC
               GTGCGGCTGCGCGGCCATTGAGACCCATTGGAATAGTAATTG--AGGGTGATAGCAGAAC
               GTG-CGCTGCGCGGCCATTGAGACCCATTGGAATAGTAATTG--AGGGTGATAGCAGAAC
               GTGCCGCTGCGCGGCCATTGAGACCCATTGGAATAGTAATTG--AGGGTGATAGCAGAAC
               GTGCCGCTGCGCGGCCATTGAGACCCATTGGAATAGTAATTG--GTGA----AGCAGAAC
               GTGCCGCTGCGCGGCCATTGAGACCCATTGGAATAGTAATTC--AGGGTGATAGCA-AAC
               GTGCCGCTGCGCGGCCATTGAGACCCAT--------------------------------
               GTGCCGCTGCGCGGCCATTGAGACCCATTGGAATACT-------AGGGTGATAGCAGATC
               GTGCCGCTGCGCGGCCATTGAGACCCATTGGAATAGTAATTG--AGGGTGATAGCAGAAC
               GTGCCGCTGCGC-GCCATTGAGACCCATTGGAATAGTAATTG--AGGGTGATAGCAGAAC
               GTGCCGCTGCGC-GCCATTGAGACCCATTGGAATAGTAATTGAAAAGGTGATAGCAGAAC
               ***  ******* ***************
```

```
Reference        GGGGAAGACGATGTGGGCTAGGTCATTGGGACCACATAATTATCTGTGTGGCCAT-----
AVTO2226-2       GGGGAAGACGATGTGGGCTAGGTCATTGGGACTACATAATTATCTGTGTGGCCAT-----
AVTO2226-3       GGGGAAGACGATGTGGGCTAGGTCATTGGG--CACCACATTATCTGTGTGGCCAT-----
AVTO2226-4       GGGGAAGAC-ATGTGGGCTAGGTCATTGGGACCACATAATTATCTGTGTGGCCAT-----
AVTO2226-5       GGGTAAGACGATGTGGGCTAGGTCATTGGGACCACATAATTATCTGTGTGGCCAT-----
AVTO2226-6       GGGGAAGACGATGTGGGCTAGGTCATTGGGACCACATAATTATCTGTGTGGCCAT-----
AVTO2226-7       GGGGAAGACGATGTGGGCTAGGTCATTGGGACCACATAATTATCTGTGTGGCCAT-----
AVTO2226-8       ------------------------------------TAATTATCTGTGTGGCCAT-----
AVTO2226-9       GGGGAAGACGATGTGGGCTAGGTCATTGGGACCACATAATTATCTGTGTGGCCATGGCCC
AVTO2226-10      GGGGAAGACGATGTGGGCTAGGTCATTGGGACCACATAATTCTGTGTG-----AT-----
AVTO2226-11      GGGGAAGACGATGTGGGCTAGGTCATTCGGACCACATAATTATCTGTGTGGCCAT-----
AVTO2226-12      GGGGAAGACGATGTGGGCTAGGTCATTCGGACCACATAATTATCTGTGTGGCCAT-----
                                                       *** * ****     **

                 TTAGA-TTTAAGTCCA--AAAGTGTACAATAA-TAATGTGTGGTACAACGTCATCGATGA
                 TTAGA-TTTAAGTCCA--AAAGTGTACAATAA-TAATGTGTGGTACAACGTCATCGATGA
                 TT-GA-TTTAAGTCCA--AAAGTGTACAATAA-TAATGTGTGGTACAACGTCATCGATGA
                 TTAGA-TTTAAGTCCA--AAAGTGTACAATAA-TAATGTGTGGTACAACGTCATCGATGA
                 TTAGA-TTTAAGTCCA--AAAGTGTACAATAA-TAATGTGTGGTACAACGTCATCGATGA
                 TTA---------------------------------------------------------
                 TTAGATTTTTTGTCCA--AAAGTGTACAATAA-TAATGTGTGGTACAACGTCATCGATGA
                 TTAG---TTAAGTCCA--AAAGTGTACAATAACCGGTGTGTGGTACAACGTCATCGATGA
                 TTAGA-TTTAAGTCCA--AAAGTGTACAATAA-TAATGTGTGGTACAACGTCATCGATGA
                 TTAGA-TTTAAGTCCACCGGAGTGTACAATAA-TAATGTGTGGTACAACGTCATCGATCA
                 --AGA-TTTAAGTCCA--AAAGTGTACAATAA-TAATG----------------------
                 --AGA-TTTTAGTCCA--AAAGTGTACAATAA-TAATG----------------------


                 CGTCGATCCGCACTATCTAAAACACTTTAAA-GAATTCATGGGGGCCCA-AAGGGACTGG
                 TGTCGATCCGC-CTATCTAAAACACTTTAAA-GAATTCATGGGGGCCCA-AACGGACTGG
                 CGTCGATCCGCACTATCTAAAACACTTTAAA-GAATTCATGGGGGCCCC-AAGCGTACGG
                 CGTCGATCCGCACTATCTAAAACACTTTAAA-GAATTCATGGGGGCCCA-AAGGGAC---
                 CGTCGATCCGCACTATCTAAAACACTTTAAA-GAATTCATGGGGGCCCA-AAGGGACTGG
                 -------------TATCTAAAACACTTTAAA-GAATTCATGGGGGCCCA-AAGGGACTGG
                 -GTCGATCCGCACTATCTAAAACACTTTAAA-GAATTCATGGGGGTCCA-AAGGGACT-G
                 CGTCGATCCGCACTATCTAAAACACTTTAAA-GAATTCATGGGGGCCCA-AAGGGACTGG
                 CGTCGATCCGCACTATCTAAAACACTTTAAA-GAATTCATGGGGGCCCA-AAGGGACTGG
                 CGTCG--CCGCACTATCTAAAACACTTTAAATTAATTCATGGGGGCCCACGAGGGACTGG
                 ------------------------------------------------------------
                 ------------------------------------------------------------


                 CAATCAAACACAAAGTACGGAAAGCCAGTTCAAATTAAAGGCGGAATTCCCACTATCTTT
                 CAATGAAACACAAAGTAC-GAAAGCCAGTTCAAATTAAAGGCGGAATTCCCACTAGCTTT
                 CAATCAAACACAAAGTACGGAAAGCCAGTTCAAATTAAAGGCGGAATTCCCACTATCTTT
                 ---TCAAACACAAAGTACGGAAAGCCAGTTCAAATTAAAGGCGGAATTCCCACTATCTTT
                 CAATCAAACACA------------------------------------------------
                 CAATCAAACACAAAGTACGGAAAGCCAGTTCAAATTAAAGGCGGAATTCCCACTATCTTT
                 CAATCAAACACAAAGTACGGAAAGCCAGTTCAAATTAAAGGCGGAATTGCCACTATCTTT
                 CAATCAAACACAAAGTACGGAAAGCCAGTTCAAATTAAAGGCGGAATTCCCACTATCTTT
                 CAATCAAACACAAAGTACGGAAAGCCAGTTCAAATTAAAGGCGGAATTCCCACTATCTTT
                 CAATCAAACACAAAGTACG--AAGCCAGTTCAAATTAAAGGCGGAATTCCCACTATCTTT
                 -------------------------------------------TTCCCGCTATCTTT
                 -------------------------------------------TTCCCGCTA--TTT
```

```
Reference       CTCTGC-AATCCTGGACCC--AACTCCAGTTATAAAGAGTACTTG---GAAGAAGAA---
AVTO2226-2      CACTGC-AATCCTGGACCC--AACTCCA-TTATAAAGAGTACTTG---GAAGAAGAA---
AVTO2226-3      CACTGC-AATCCTGGA-CC--AACTCCAGTTATAAAGAGTACTTG---GAAGAAGAA---
AVTO2226-4      CTCTGC-AATCCTGGACCTTAAACTCCAGTTATAAAGAGTACTTG---GAAGAAGAA---
AVTO2226-5      ------------------------------------------------------------
AVTO2226-6      CTCTGC-AATCCTGGACCC--AACTCCAGTTATAAAGAGTACTTGGCCGGAGAAGAA---
AVTO2226-7      CTCTGC-AATCCTGGACCC--AACTCCAGTTATAAAGAGTACTTG---GAA-ATTAA---
AVTO2226-8      CTCTGC-AATCCTGGACCC--AACTCCAGTTATAAAGAGTACTTG---GATTAAGAA---
AVTO2226-9      CTCTGC-AATCCTGGACCC--AACTCCAGTTATAAAGAGTACTTG---GAAGAAGAAAAG
AVTO2226-10     CTCTGCTTATCCTGGACCC--AACTCCAGTTATAAAGAGTACTTG---GAAGAAGAA---
AVTO2226-11     CTCTGC-AATCCAGGACCC--AACTCCAGTTATAAAGAGTACTTG----GAGAAGAA---
AVTO2226-12     CTCTGC-AATCCAGGACCC--AACTCCAGTTATAAAG---ACTTG----GAGAAGAA---

                --AAGAACT-CCGCACTTA-----GA-AACTGGGCTATAAG-AAATGCGATCTTCGTCAC
                --AAGAACT-CCGCACTTA-----GA-AACTGGGCTATAAG-AAATGCGATCTTCGTCAC
                --AAGAACT-CCGGACTTA-----GATAAGTGGGCTATAAG-AAATGCGATCTTC-TCAC
                --AAGAACT-CCGCACTTA-----GA-AACTGGGCTATAAG-AAATGCGATCTTCGTCAC
                --------------------------------------------------------TCAC
                --AAGAACT-CCGCACTTA-----GA-AACTGGGCTATAAG-AAATGCGATCTTCGTCAC
                --AAGAACT-CCGCACTTA-----GA-AACTGGGCTATAAG-AAATGCGATCTTCA-AAC
                --AAGAACT-CCGCACTTA-----GA-AACTGGGCTATAAG-AAATGCGATCTTCGTCAC
                TTAAGGCCT-CCGCACTTA-----GA-AACTGGGCTATAAG-AA----------------
                --AAGAACT-CCGCACTT---------AACTGGGCTATAAG-AAA---GATCTTCGTCAC
                --AAGAACT-CGGCACTTAGCCCGGA-AACTGGGCTATAAGAAAATGCGATCTTCGTCAC
                --AAGAACTCCGGCACTTAGCCCGGA-AACTGGGCTATAAGAAAATGCGATCTTCGTCAC

                CCT--CAAAAGCCCACTCTACTCAGGTTCCAATCAAGGTGCAACA----CCG-AATAGCC
                CCT--CAAAAGCCCACTCTACTCAGGTTCCAATCAAGGTGCAACA----CCG-AATAG--
                CCT--CAAAAGCCCACTCTACTCAGGTTCCAATCAAGGTGTAACACCACCCG-AATAGCC
                CCT--CAAAAGCCCACTCTACTCAGGTTCCAATC------CAACA----CCG-AATAGCC
                CCT--CAAAAGCCCACTCTACTCAGGTTCCAATCAAGGTGCAACA----CCG-AATAGCC
                CCT--CAAAAGCCCACTCTACTCAGGTTCCAATCAAGGTGCAACA----CCG-AATAGCC
                CCT--CAAAGCCCACTGGGACTCAGGTTCCAATCAAGGTGCAACA----CCGTTATAGCC
                CCT--CAAAAGCCCACTCTACTCAGGTTCCAATCAAGGTGCAACA----CCG-AATAGCC
                -----------------------------------------------------------C
                TTTAACAAAAGCCCACTCTACTCAGGTTCCAATCAAGGTGCAACA----CCG-AATAGCC
                CCT---CAAAGCCCACT---CTCAGGTTCCAAACAAGGTGCAACA----CCG-AATAGCC
                CCT---CAAAGCCCACT---CTCAGGTTCCAAACAAGGTGCAACA----CCG-AATAGCC

                ---------AAGAGGGCAACCAGACGACGGAGAGTTGA
                ---------AAGCGGGCAACCGGACGACGGAGAGTTGA
                ---------AAGAGGGCAACCAG-CGACGGAGAGTTGA
                ---------AAGAGGGCAACCAGACGACGGAGAGTTGA
                ---------AAGAGGGCAACCAGACGACGGAGAGTTGA
                ---------AA-----CAACCAGACGACGGAGAGTTGA
                ---------AAGAGCGCAACCAGACGACGGAGAGTTGA
                ---------AAGAGGGCAACCAGACGACGGAGAGTTGA
                ---------AAGAGGGCAACCAGACGACGGAGAGTTGA
                ---------AAGAGGGCAACCAGACGACGGAGAGTTGA
                TAAAGGGGAAGAGGGCGACCAGACGACGGAGAGTTGA
                TAAAGGGGAAGAGGGCGACCAGACGACGGAGAGTTGA
                         **     *.***.* **************
```

d

```
Reference      AT---GCGATCTTCGTCACCCTCAAAAGCCCACTCTACTCAGGTTCCAATCAAGGTGCAA
AVTO2226-2     AT---GCGA----TGTCACCCTCAAAAGCCCACTCTACTCAGGTTCCAATCAAGGTGCAA
AVTO2226-3     A----GCGATCTTCGTCACCCTCAAAAGCCCACTCTACTCAGGTTCCCATCAAGGTGCAA
AVTO2226-4     T----GCGATCTTCGTCACCCTCAAAAGCCCACTCTACTCAGGTTCCAATCAAGGTGCAA
AVTO2226-5     AT---GCGA----TGTCACCCTCAAAAGCCCACTCTACTCAGGTTCCAATCAAGGTGCAA
AVTO2226-6     AT---GCGA----TGTCACCCTCAAAAGCCCACTCTACTCAGATTCCAATCTAGGTGCAA
AVTO2226-7     AT---GCGATCTTCGTCACCCTCAAAAGCCCA--CTACTCAGGTTCCAATCAAGGTGCAA
AVTO2226-8     ATCCCCGATCTTCGTCACCCTCAAAAGCCCAGGGTACTCAGGTTCCAATCAAGGTGCAA
AVTO2226-9     A----TCGATCTTCGTCACCCTCAAAAGCCCACTCTACTCAGGTTCCAATCAAGGTGCAA
AVTO2226-10    AG---CCGATCTTCGTCACCCTCAAAAGCCCACTCTACTCAGGTTCCAATCAAGGTGCAA
AVTO2226-11    AT---CCGA-TCTCGTCACCCTCAAAAGCCCACTCTACTCAGGTTCCAATCAAGGTGCAA
AVTO2226-12    AT---CCGATCTTCGTCACCCTCAAAAGCCCACTCTACTCAGGTTCCAATCAAGGTGCAA
                     ***    .******************   *******.**** *** ********

               CACC---GAATAGCCAAGAGGGCAACCAGACGACGGAGAGTTGATCTACCTTGTGGATGT
               CACC---GAATAGCCAAGAGGGCAACCAGACGACGGAGAGTTGATCTACCTTGTGGATGT
               CACC---GAATAGCCAAGAGGGC-ATCAGACGACGGAGAGTTGATCTACCTTGTGGATGC
               CACC---GAATAGCCAAGAGGGCAACCAGACGACGGAGAGTTGATCTACC-TGTGGATG-
               CACC---GAATAGCCAAGAGGGCAACCAGACGACGGAGAGTTGATCT------------T
               CACC---GAATAGCCAAGAGGGCAACC-GACGACGGAGAGTTGATCTACCTTGTGGATGT
               CACC---GAATAGCCAAGAGGGCAACCAGACGACGGAGAGTTGATCTACCTTGTGGATGT
               CACC---GAATAGCCAAGAGGGCAACCAGACGACGGAGAGTTGATCTA------------
               CACCGGGGAATAGCCAAGAGGGCAACCAGACGACGGAGAGTTGATCTACCTTGTGGATGT
               CACC---GAATAGCCAAGAGGGCAACCAGACGACGGAGAGTTGATCTACCTTGTGGATGT
               CACC---GAATAGCCAAGAGGG-AACCAGACGACGGAGAGTTGATCTACCTTGTGGATGT
               CACC---GAATAGCCAAGAGGGCAACCAGACGACGGAGAGTTGATCTACCTTGTGGATGT
               ****   ***************  *.* *******************

               TCGTACTTCGTCGCAATAGGATGTCACAATAATGGATTTACGCACCGGGGAACTACTCAC
               TCGTACTTCGTCGCAATAGGATGTCACAATAATGGATTTACGCACCGGG--ACTACTGAC
               TCGTACTTCGTCGCAATAGGATGTCACAATAATGGATTTACGCACCGGGGAACTACTCAC
               -CGTACTTCGTCGCAATAGGATGTCACAATAATGGATTTACGCA--GGGGAACTACTCAC
               TCGTACTTCGTCGCAATAGGATGTCACAATAATGGATTTACGCACCGGG--ACTACTGAC
               TCGTACTTCGTCGCAATAGGATGTCACAATAATGGATTTACGCACCGGGGAACTACTCAC
               TCGTACTTCGTCGCAATAGGATGTCACAATAATGGATTTACGCACCGGGGAA--------
               ---------GTCGCAATAGGATGTCACAATAACCTGGATTTACGCCGGGGAACTACTCAC
               TCGTACTTCGTCGCAATAGGATGTCACAATAATGGATTTACGCACC--GGAACTACTCAC
               TCGTACTTCGTCGCAATAGGATGTCCGAATAATGGATTTACGCACCGGGGAACTACTCAC
               TCGTACTTCGTCGCAATAGGATGTCACAATAATGGATTTACGCACCGGGGAACTACTCAC
               TCGTACTTCGTCGCAATAGGATGTCACCTGGACTCATTTACATCCTCAGACCTCGCGTTT
                        ****************     .*.  .  * ...     *

               TGCAACTCAATTAGAGAGTGGCGTGTATATCTGGACGCTCAA----AAATCCCCTATATT
               TGAAACTCAATTAGAGAGTGGCGTGTATATCTGGACGCTCAA----AAATCCCCTATATT
               TGCAACTCA-------AGTGGCGTGTATATCTGGGCGCTCAA----AAATCCCCTATATT
               TGCAA--CAATTAGAGAGTGGCGTGTATATCTGGACGCTCAA----AAATCCCCTATATT
               TGAAACTCAATTAGAGAGTGGC--GTATATCTGGACGCTCAA----AAATCCCCTATATT
               GGCAACTCAATTAGAGAGTGGCGTGTATATCTGGACGCTCAA----AAATCCCCTATATT
               -GCAACTCAATTAGA-AGTGGCG-GTATATCTGGACGCTCAA----AAATCCCCTATATT
               TGCAACTCAATTAGAGAGTGGCGTGTATATCTGGACGCTCAA----AAATCCCCTATATT
               TGCAACTCAATTAGA------------------GACGCTCAA-CCCAAATCCCCTATATT
               TGCAACTCAATTAGAGAGTGGCGTGTATATAATCACGTTACA----AATCCAGTTCAACC
               TGCAACTCAATTAGAGAGTCGCGTGTATATCTGGACGCTCACGCTAAAATCCCCTATAGT
               CTTAACAGTATT------TAACACTCAAAGGCGGTAATACGG----TTATCCACAGAATC
                  **                               ..           .*  .   * .
```

```
Reference      TCAAGATAACCAAGCACCTAGAGAGCCCATTCCAGAGGAACCACGACATAATCACGTTAC
AVTO2226-2     TCAACATAACCAAGCACCTAGAGAGCCCATTCCAGAGGAACCACGACATAA---------
AVTO2226-3     TCAAGATAACCAAGCACCTAGAGAGCCCATTCCAGAGGAACCACGACATAA---------
AVTO2226-4     TCAAGATAACCAAGCACCTAGAGAGCCCATTCCAGAGGAACCACGACATAA---------
AVTO2226-5     TCAACATAACCAAGCACCTAGAGAGCCCATTCCAGAGGAACCACGACATAATCACGTTAC
AVTO2226-6     TCAAGATAACCAAGCACCTAGAGAG-CCATTCCAGAGGAACCACG-CATAA---------
AVTO2226-7     TCAAGATAACCAAGCACCTAGAGAGCCCATTCCAGAGGAACCACGACATAA---------
AVTO2226-8     TCAAGATAACCAAGCACCTAGAGAGCCCATTCCAGAGGAACCACGACATAATCACGTTAC
AVTO2226-9     TCAAGATAACCAAGCACCTAGAGAGCCCATTCCAGAGGAACCACGACATAATCACGTTA-
AVTO2226-10    ACAACCTGAGGAAAGCGTTGGGAT---------------------ACACAAATGTTT---
AVTO2226-11    GCGTAGATTTCAACCACAACCTGAGGAAAGCGTTGVGGGAT----ACACAAATGTTTTCT
AVTO2226-12    AGGGGATAACGCAG------GAAAGAACATGTGAGCAAAAGGCCAGCAAAA---------
                 .         *         .*                      ** **

               AAATCCAGTTCAACCACAACCTGAGGAAAGCGTTGGGGATACACAAATGTTTTCTAGTCT
               ------------------------------------------------------------
               ------------------------------------------------------------
               ------------------------------------------------------------
               AA----------------------------------------------------------
               ------------------------------------------------------------
               ------------------------------------------------------------
               ------------------------------------------------------------
               ------------------------------------------------------------
               ------------------------------------------------------------
               AG-------------------------------------------------------TCT
               GG-------------------------------------------------------CCA

               GCAAAATCT-GGACTCATTTACATCCTCAGACCTCGCGTTTCTTAACAGTATTTAA
               --------------------------------------------------------
               --------------------------------------------------------
               --------------------------------------------------------
               --------------------------------------------------------
               --------------------------------------------------------
               --------------------------------------------------------
               --------------------------------------------------------
               --------------------------------------------------------
               --------------------------------------------------------
               GCAAAATCTGGGACTCATTTACATCCTCAGACCTCGCGTTTCTTAACAGTATATAA
               GGAA----------------------------------------------------
```

Figure S7

Comparison of additional *AC1* and *AC2* DNA sequences obtained from TYLCTHV particles isolated from AVTO2225-R and AVTO2226 plants after infection. **a**. Comparison of *AC1* DNA sequences from TYLCTHV particles isolated from AVTO2225-R-7, 9 and 11 plants. **b**. Comparison of *AC2* DNA sequences from TYLCTHV particles isolated from AVTO2225-R-7, 9 and 11 plants. **c**. Comparison of *AC1* DNA sequences from TYLCTHV particles isolated from AVTO2226 plants **d**. Comparison of *AC2* DNA sequences from TYLCTHV particles isolated from AVTO2226 plants. * indicates no nucleotide changes in *AC1* and AC2 DNA sequences from TYLCTHV particles isolated from the plants.
